# Designed and biologically active protein lattices

**DOI:** 10.1038/s41467-021-23966-4

**Published:** 2021-06-17

**Authors:** Shih-Ting Wang, Brian Minevich, Jianfang Liu, Honghu Zhang, Dmytro Nykypanchuk, James Byrnes, Wu Liu, Lev Bershadsky, Qun Liu, Tong Wang, Gang Ren, Oleg Gang

**Affiliations:** 1 Center for Functional Nanomaterials, Brookhaven National Laboratory, Upton, NY USA; 2 Department of Chemical Engineering, Columbia University, New York City, NY USA; 3 The Molecular Foundry, Lawrence Berkeley National Laboratory, Berkeley, CA USA; 4 Energy Sciences Directorate/Photon Science Division, NSLS II, Brookhaven National Laboratory, Upton, NY USA; 5 Biology Department, Brookhaven National Laboratory, Upton, NY USA; 6 Advanced Science Research Center at the Graduate Center of the City University of New York, New York City, NY USA; 7 Department of Applied Physics and Applied Mathematics, Columbia University, New York, NY USA

**Keywords:** SAXS, Biomaterials, Molecular self-assembly, Organizing materials with DNA, Nanostructures

## Abstract

Versatile methods to organize proteins in space are required to enable complex biomaterials, engineered biomolecular scaffolds, cell-free biology, and hybrid nanoscale systems. Here, we demonstrate how the tailored encapsulation of proteins in DNA-based voxels can be combined with programmable assembly that directs these voxels into biologically functional protein arrays with prescribed and ordered two-dimensional (2D) and three-dimensional (3D) organizations. We apply the presented concept to ferritin, an iron storage protein, and its iron-free analog, apoferritin, in order to form single-layers, double-layers, as well as several types of 3D protein lattices. Our study demonstrates that internal voxel design and inter-voxel encoding can be effectively employed to create protein lattices with designed organization, as confirmed by in situ X-ray scattering and cryo-electron microscopy 3D imaging. The assembled protein arrays maintain structural stability and biological activity in environments relevant for protein functionality. The framework design of the arrays then allows small molecules to access the ferritins and their iron cores and convert them into apoferritin arrays through the release of iron ions. The presented study introduces a platform approach for creating bio-active protein-containing ordered nanomaterials with desired 2D and 3D organizations.

## Abstract (web summary)

Organising proteins in 2D and 3D is needed to develop complex bimolecular materials for a range of applications. Here, the authors report the encapsulation of ferritin and apoferritin in DNA-based voxels with programmed assembly to generate both 2D and 3D protein lattices and demonstrate the retention of protein function.

## Introduction

The assembly of proteins, nature’s most powerful and versatile building blocks, into rationally organized arrays is of substantial interest for enabling protein-containing materials and their cell-free functionality^1–4^. Establishing approaches for creating protein-based well-ordered structures has been a long-standing focus of structural biology for revealing their atomic structures^5^. Although such protein crystals can teach us about the bio-machinery through detailed structural information, crystallization methods are not compatible with creating biologically active protein organizations, nor exploring their function in operando. The need for developing methods to design and form 3D functional protein arrays has become even more apparent for addressing outstanding problems in cellular^6–9^ and tissue engineering^10,11^, fabrication of multi-enzyme systems^12,13^, for proteomic profiling^14^ and synthetic biology applications^15^.

Establishing methodology for building desired organizations of proteins remains challenging due to the varied and transient nature of protein shapes, and chemical and charge heterogeneities of their surfaces. Thus, individual systems typically require unique approaches, such as optimization of conditions in protein crystallography^5^ and protein coacervation^9^, design of protein interfaces^16–18^, and use of protein cages^2,19,20^. It is highly attractive to consider methods for assembling proteins into desired spatially organized systems.

The original idea of using DNA as a structural material, proposed by Seeman^21^ almost four decades ago, strived to address a challenge faced by protein crystallography in generating atomically defined 3D protein organizations. The idea has given rise to the field of DNA and RNA nanotechnology, where sequentially defined polynucleotide polymers can be programmed into different structural states to form designed nucleic acids constructs^22–28^ that can be assembled in ordered 2D^29–31^ and 3D organizations^32–37^, with different degrees of structural fidelity. Structural control offered by DNA-based methods is highly attractive for creating 3D nanoscale organizations of functional inorganic nanoparticles^33,38–44^. Recent efforts demonstrate that designed DNA constructs can be coupled with proteins, allowing for applications in bio-catalysis^45,46^, nanomedicine^47–51^, probing biological structures^52,53^ and processes^6,54,55^, and manipulation of protein functionalities^13,56^. Although it was demonstrated that enzymes can be organized in space^57^ and their 3D ordered cascades^33^ can exhibit an enhanced activity, gaining an engineering control over the organization of biologically functional proteins, which often comprise multiple subunits, and symmetric or asymmetric domains, remains an elusive and highly desirable goal.

The quest for orchestrating the assembly of one or many types of proteins into a bio-active and fully prescribed 3D scaffold has become more evident with a rapid conversion of nanotechnology and synthetic biology towards developing molecularly controlled systems. Several challenges have to be solved in order to establish a broadly applicable strategy for creating bio-active protein arrays, regardless of their shape and surface groups, while maintaining an environment and molecular transport for their operation. They include: (i) “transparency” for molecular transport, (ii) a structural designability; and (iii) a broad protein integration suitability. These problems, as we show below, can be tackled by DNA-based approaches by trading a complex inter-protein interaction for Watson–Crick base-pairing, which can be controlled on multiple length scales.

In this work, we demonstrated that DNA material voxels, polyhedral DNA frames with encapsulated nano-objects, could integrate with proteins and be adapted for assembly of ferritin, a biological functional iron storage protein macromolecule (~450 kDa, 24 subunits) and its coreless, iron-free form, apoferritin, into desired ordered arrangements, including single-layer, double-layer, and different types of 3D lattices. Here, we created functional protein arrays by combining internal voxel design, which tailored placement of protein inside DNA voxels, and inter-voxel encoding, which provided connectivity control. The work showed that assembled proteins remained active in a lattice and their state could be structurally monitored in situ. The detailed probing revealed an excellent correspondence between electron microscopy (EM), EM-based tomography, and small-angle X-ray scattering (SAXS), while SAXS further captured iron ion release kinetics from the ordered 3D ferritin arrays. This demonstrated a feasibility for creating organized protein systems as fully designed protein-based materials with preserved inherent characteristics and biological activity, and as a general methodology for investigating protein conformations and interactions.

**Fig. 1:**
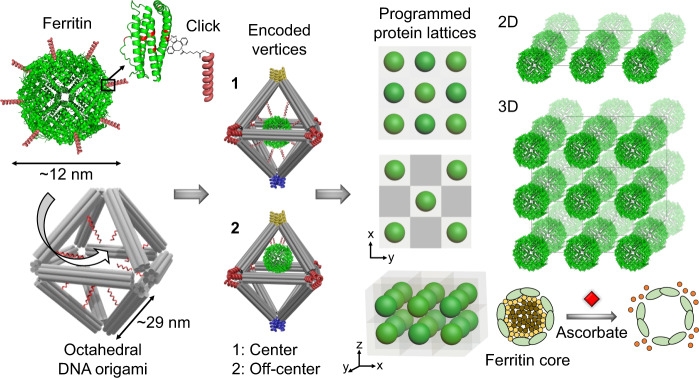
The concept of programmable assembly of bio-active 2D and 3D protein arrays by integrating ferritin with DNA frames (octahedra, Octa) into protein voxels. A high-yield stable frame-encapsulation of ferritin and apoferritin into protein voxels and their assembly into ordered arrays through a stepwise modification of protein (ferritin, PDB ID: 1IER) surfaces (at the lysine residues (red)). A targeted placement of proteins, encoded with DNA sequences, at the frame’s center (1) and shifted toward a vertex (2). Various positions of the encapsulated ferritin (see “Methods” section and Supplementary Fig. 7 for design in detail) inside the frame can be prescribed, as discussed later in the text. The designed 2D single- and double-layered, and 3D ferritin and apoferritin lattices were formed by establishing prescribed vertex-to-vertex Watson–Crick interconnections. The 3D ordered ferritin array could be converted into a coreless apoferritin array with preserved structure using ascorbate to reduce and release iron ions (orange circles) from ferritin.

Depicted in Fig. 1 is the concept of our developed approach for assembly of biologically functional proteins into ordered arrays through programmable DNA frameworks that can host and control the placement of the guest proteins within each voxel. To demonstrate the applicability of the proposed concept, our selected proteins, ferritins, and apoferritins were grafted with single-stranded (ss) DNA through covalent chemistry to facilitate high efficiency (~70%) of encapsulation inside the 3D wire-framed octahedral origami (Octa, Supplementary Figs. 1 and 2)^40,42^ via hybridization with complementary ssDNA at designated sites of Octa. This strategy generates a “protein voxel” with a tailored ferritin or apoferritin placement within. In order to create 2D and 3D protein arrays, we encode the specific voxels’ vertices to facilitate a desired inter-vertex hybridization and to drive the assembly of designed arrays. A combination of the two strategies, a protein voxel and programmable assembly of voxels, offers control over the formation of protein arrays with prescribed lattice symmetries. The open architecture of the individual frames and assembled framework provide a native protein environment and molecular accessibility to proteins (Fig. 1). We demonstrate that the structural stability of ferritin and its biological activity to release iron ions are maintained within the assembled arrays.

## Results

**Fig. 2:**
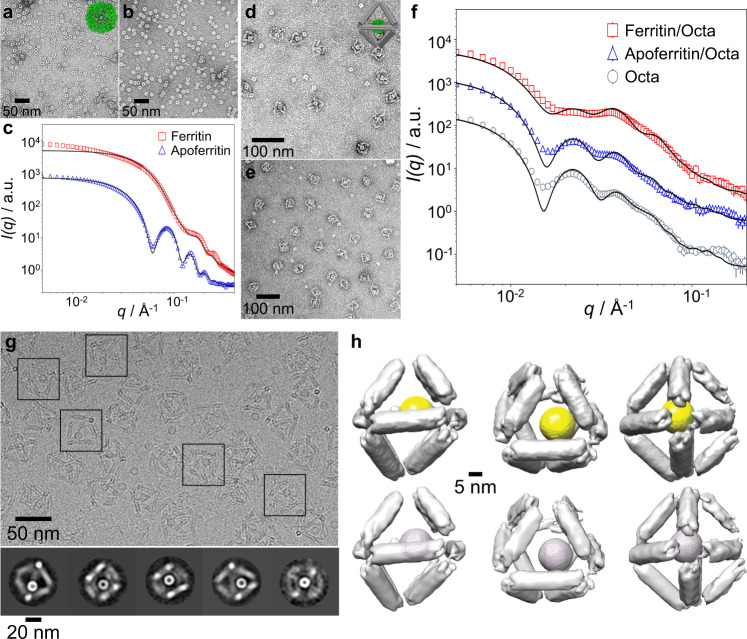
Protein voxels: ferritin and apoferritin placed in the center (position Off0) of Octa frames, and characterized by in situ SAXS, ex situ TEM, and cryo-EM. **a**, **b** Negative-stained TEM imaging (**a** ferritin, **b** apoferritin) and **c** SAXS measurement and analysis of free ferritin and apoferritin in solution (intensity profiles were shifted vertically for clarity). **d**, **e** Negative-stained TEM imaging (**d** ferritin/Octa, **e**: apoferritin/Octa) and **f** SAXS data and analysis of ferritin and apoferritin encapsulated inside Octa (intensity profiles were shifted vertically for clarity). The insets in **a** and **d** show illustrated views of ferritin (PDB ID: 1IER) and ferritin/Octa. **g** Cryo-EM micrograph (top row) and 2D reference-free class average (bottom row) provided representative views of apoferritin/Octa. The black squares enclose the representative structures. **h** Surface-rendered 3D density maps of apoferritin/Octa, viewed from the two-fold (left), three-fold (middle), and four-fold (right) symmetry axes. In the bottom view, the apoferritin model (PDB ID: 2W0O) is docked in the density of apoferritin.

The selected proteins, ferritin and apoferritin from the horse spleen (Sigma), were composed of 24 subunits and shared a shell topology with ferritin containing a distinctive iron core. Each subunit consisted of ~9 primary amines (PDB IDs: 1IER and 2W0O) which enabled surface modification using DNA. We first analyzed, using negative-stained TEM imaging, ferritin, and apoferritin, and both showed average sizes of ~12 nm at dry states (Fig. 2). The native proteins in solution were probed by in situ SAXS, where both proteins exhibited oscillations. Ferritin showed a smaller amplitude and period of oscitations than apoferritin (Fig. 2c and Supplementary Fig. 3), and these observations were in good agreement with our scattering models, accounting for the core-shell protein structure (see “Methods” section). The fitted form factors revealed overall diameters of ~12 nm with 7–8 nm iron core and cavity for ferritin and apoferritin, respectively (Fig. 2c). The close correspondence between TEM and SAXS was also in agreement with crystallographic studies, exhibiting core and shell diameters of about 8 and 12 nm, respectively^58,59^.

To efficiently encapsulate proteins in the frame, we established a two-step chemical conjugation process, in which the primary amines of ferritin or apoferritin were first activated by a bifunctional crosslinker through *N*-hydroxysuccinimde (NHS)-amine reaction, followed by covalent coupling of the dibenzocyclooctyne (DBCO) modified ssDNA using click chemistry (see “Methods” section). Agarose gel electrophoresis and dynamic light scattering (DLS) confirmed the successful protein modification, and UV-vis analyses indicated about 6‒7 ssDNAs were attached per ferritin (Supplementary Figs. 4‒6). The ssDNA-grafted proteins were mixed with Octa frame, which had complementary ssDNA chains extended from select DNA bundles to position guest proteins at prescribed positions inside Octa (Supplementary Fig. 7), followed by heating to 48 °C and slowly cooling to room temperature. For symmetric encapsulation of proteins (Fig. 2 and Supplementary Fig. 7), we positioned them at the center of the Octa frame (termed Off0) using eight internal anchoring ssDNA, located on four meridian bundles that complemented with *l* = 15 nucleotides (nt) of ssDNA on proteins, with remaining ssDNA parts of *m* = 24 nt and *n* = 3 nt on the bundles and the protein, respectively. A protein position inside Octa could be modulated by changing the length and position of the internal anchoring strands (Supplementary Fig. 7). Negative-stained TEM imaging confirmed protein encapsulation inside Octa by the additional globular shape at the Octa center (Fig. 2 and Supplementary Figs. 8,9), and from ~400 imaged single particles, encapsulation yields were estimated as ~79% and ~70% for ferritin and apoferritin, respectively. Although for smaller proteins, the single-molecule fluorescence approach can be used to quantify the encapsulation^60^, the size of ferritin and apoferritin permits a direct quantification of the encapsulation using electron microscopy.

The integrity of Octa frame after protein encapsulation is important for the robust assembly of protein arrays. Neither electrophoresis nor DLS detected significant Octa changes after encapsulation (Supplementary Figs. 10 and 11). Next, we performed in situ SAXS measurements to analyze protein voxels. The form factor of an empty Octa (Fig. 2f and Supplementary Fig. 12) had the first two local maxima at the momentum transfer values of *q*_1_ = 0.022 Å^–1^ and *q*_2_ = 0.038 Å^–1^. Ferritin and apoferritin voxels exhibited an intensity reduction at *q*_1_ and an increase at *q*_2_, suggesting co-localization of Octa and proteins. For quantitative understanding, we applied a SAXS model with rigid Octa constructed from twelve cylinders and protein in the center. Our fitting (see “Methods” section) resulted in a cylinder’s length of 27.8 nm, which agreed with the nominal design of 28.6 nm Octa edge and a protein voxel (Fig. 2f and Supplementary Fig. 13)^33,61^. The deviations from the fitted results are likely due to the possible distortions of the octahedron and some fluctuations of its shape, which is also indicated by cryo-EM measurements, as described below.

To reveal the structural details in 3D and in real space, Octa and protein/Octa were further analyzed by single-particle reconstruction by cryo-EM imaging. Using reference-free 2D class averages (Supplementary Fig. 14), the 3D density maps of Octa with a resolution of ~22.9 Å were reconstructed, which revealed the octahedral frame with edge lengths of ~29 nm, which agreed with both our design and the SAXS results (Supplementary Fig. 13). Note that some distortion at the bundle connecting vertices was observed, likely due to internal stress. For cryo-EM imaging of protein/Octa, we selected apoferritin, of which its electron density was comparable to Octa. Figure 2g, h shows that the 2D class averages of raw particle images of apoferritin/Octa were nearly identical to those of Octa (Supplementary Fig. 14) except for the extra circular density at the frame’s center. Remarkably, apoferritin/Octa distinguished from Octa showing the additional spherical 3D density in the Octa’s cavity and that the crystal structure of apoferritin was docked well into the averaged spherical 3D densities, suggesting that apoferritin was proximate to the designed Octa’s center. A slightly off-centered positioning of apoferritin inside Octa was likely due to unformed DNA-protein linkages or minor distortions of Octa.

**Fig. 3:**
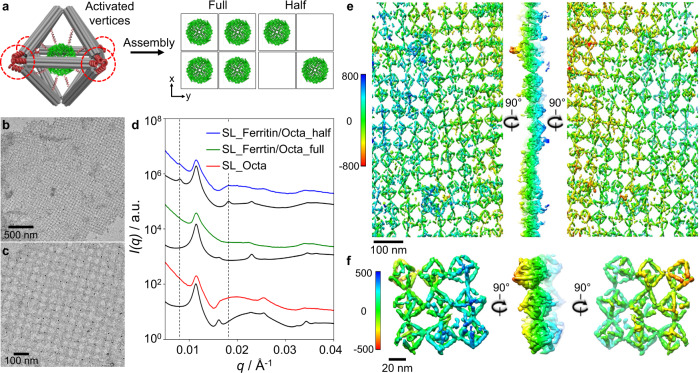
Ferritin assembly in ordered 2D single-layered array. **a** Schematics showing a projected view of 2D single-layered (SL) protein/Octa lattices, where the in-plane vertices were encoded by one set of complementary DNA pairs (i.e., a one-color system). Ferritin was placed at the center of Octa for assembly into a fully integrated lattice, and a half-filled lattice by alternating the ferritin/Octa and empty Octa in the same lattice. **b**, **c** Negative-stained TEM images of SL ferritin/Octa lattices, where **c** shows a magnified image of the lattice. **d** SAXS data and analyses of SL ferritin/Octa lattices, in which the experimental data (colored lines) and the corresponding simulated results (black lines) were presented for Octa and the different types of ferritin/Octa lattices (measured and calculated intensity profiles were shifted vertically for clarity). **e**, **f** Cryo-ET 3D reconstruction of an SL ferritin/Octa lattice, where a series of tilted cryo-EM images were aligned and reconstructed in 3D without averaging using the IMOD and IPET software. The 3D density maps showed an overlay of the encapsulated ferritin with the SL lattice in one structure, indicating the overall conformation of the ferritin/Octa lattice. (**e**: selected lattice area and **f**: the representative views). The ferritin positions in **f** were indicated by the black arrows in Supplementary Fig. 22d. The color bars indicate *Z* height (unit: Å).

**Fig. 4:**
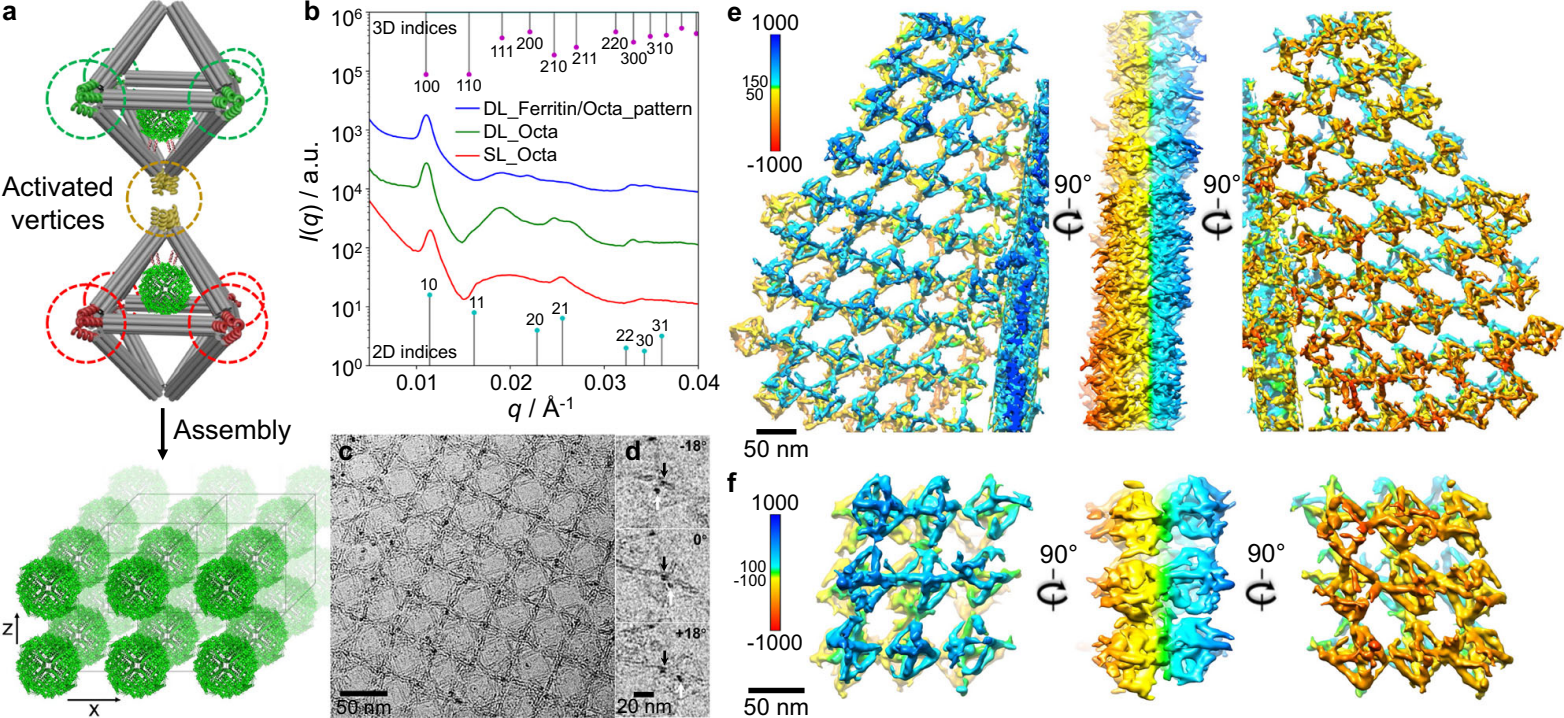
Ferritin assembly in a double-layered lattice. **a** Schematic view showing a 2D double-layered (DL) protein/Octa lattice formed by a three-color vertex encoding system consisted of encoded Octa frames with five activate vertices and three sets of complementary DNA pairs (red, green, and yellow). Protein voxel with off-centered ferritin (Off2) was used as discussed in the text. **b** SAXS profiles (shifted vertically for clarity) of the DL empty Octa and ferritin/Octa lattices show co-localization of ferritin and the Octa lattice by the reduced intensities attributed to the ferritin core. The formation of a DL structure was supported by the emergence of broad peaks at the 3D indices of a simple cubic lattice, indicating a layer in the *z* direction. **c** A cryo-ET image of a DL lattices. **d** Representative cryo-ET images showing the two ferritins in the top and bottom layers at tilted angles (black and white arrows) in a DL lattice, which agreed with our design. **e**, **f** Cryo-ET 3D reconstruction of DL ferritin/Octa lattice without averaging. The 3D density maps showed the encapsulated ferritin overlaid well with the DL lattice (**e** selected lattice area and **f** representative views, see the text and “Methods” section for details). The ferritin positions in **f** were indicated by the black arrows in Supplementary Fig. 32d. The color bars indicate *Z* height (unit: Å).

The structural stability of protein voxels allowed us to further use them as assembly “bricks” for creating targeted protein organizations. First, we constructed single- and double-layered 2D lattices (Figs. 3 and 4), where Octa vertices were encoded with distinctive ssDNA, referred to as “colored” bonds^29,62^. Here, the 2D single-layered lattices were built using two different Octa with each having four complementary encoded equilateral vertices, so-called “one-color” system (Fig. 3a and Supplementary Fig. 15). Furthermore, two lattice types were designed, where a full-filled lattice from protein voxels and a half-filled lattice using alternating protein voxels and empty voxels (Fig. 3a). A one-pot assembly procedure was applied, where a mixed solution of encoded Octa and protein was heated up to 48 °C and then slowly cooled to room temperature to ensure thermodynamically equilibrated structures for a protein encapsulation and lattice formation.

The as-formed single-layered full-filled lattices were visualized by negative-stained TEM imaging (Fig. 3 and Supplementary Figs. 16, 17). In situ SAXS analysis of the 2D empty and protein-filled voxel arrays revealed ~5 diffraction peaks (Fig. 3d and Supplementary Figs. 18, 19), indicating a square lattice with lattice constant *a*_SL-Octa_ = 55 nm for both cases. Co-localization of Octa and proteins was concluded from a reduced scattering intensity in the intermediate *q* range (0.015‒0.030 Å^−1^) and was attributed to the form factor of protein/Octa. Such results were supported by our model that took into account the form factors of protein and Octa and this type of 2D Bravais lattice. In the half-filled design (Fig. 3a), the lattice constant changed from *a*_SL-Octa_ to $\sqrt{2}$*a*_SL-Octa_ (Fig. 3d and Supplementary Figs. 18, 19), as expected.

In order to visualize the 2D lattice and ferritin positioning in real space in 3D, we used cryo-electron tomography (cryo-ET) and 3D reconstruction techniques. As shown in Supplementary Fig. 20, cryo-EM imaging of the lattice solutions embedded in vitreous ice, confirmed the micron-sized lattice domains, and distinguished the single-layered protein- and empty-voxel lattices by the dark spots from the ferritin core. Cryo-ET images were obtained from a series of tilt angles from −51° to +51° at a 3° increment. The representative tilt images (Supplementary Fig. 21) and correspondent 3D density maps (Fig. 3e, f) revealed encapsulated ferritins in the 2D lattice by the additional globular density. High-resolution 3D reconstruction was achieved by the individual particle electron tomography (IPET)^63^ with missing-wedge correction techniques^64^, by focusing on small selected areas of a lattice to avoid image distortion at a larger scale^65^. Here, an area of the ferritin/Octa lattice was selected for serial refinement to obtain a final IPET 3D density map (Fig. 3 and Supplementary Fig. 22). Nearly all 12 DNA bundles of Octa and the encapsulated ferritins (black arrows in Supplementary Fig. 22d) could be resolved at a resolution of ~225 Å. Thus, local orders and a highly preserved connectivity of the single-layered lattice in each direction, with ferritins docked well into the framework were revealed, which agreed with our design and SAXS analysis (Fig. 3d). We note a distribution of orientations among octahedra in the single-layer lattice (Fig. 3e, f), which is likely due to 2D connectivity and flexibility of the inter-voxel motifs. This is further confirmed by comparison with the double layer as we discussed below. Such an orientational distributions can contribute to the reduction of SAXS peaks comparatively to the model (Fig. 3d).

To further demonstrate a versatility of our approach for protein lattice engineering, we created lattices by designing both the protein voxel and inter-voxel assembly to generate specific lattice symmetries. For protein voxels, by changing the ssDNA’s length and position, we shifted the protein from Octa’s center (or Off0) toward a vertex at two positions, defined as Off1 and Off2. As shown in Fig. 4a and Supplementary Fig. 7 and confirmed by TEM and in situ SAXS (Supplementary Figs. 23‒25), proteins were shifted from the Off0 using 4 internal strands from the selected vertex, where the ssDNA on DNA bundles (*m*) was 15 nt for Off1 and 9 nt for Off2; both *n* and *l* remained the same as for Off0. The SAXS analysis indicated that the protein was shifted by 1.5 nm and 3.5 nm for the Off1 and Off2 designs, respectively. We utilized the off-center protein voxel (Off2), for creating a double-layered protein array with an unequal distance between proteins in the *x*–*y* and *z* directions (Fig. 4a). Our design comprised four distinct Octa and three distinct sets of complementary DNA pairs (i.e., a “three-color” system), two of which were utilized for in-plane (*x*–*y*) hybridization of vertices within each layer (Fig. 4a, green and red, Supplementary Fig. 26), and one (yellow) for connecting two layers in the out-of-plane (*z*) direction. Due to the off-centered protein in a voxel, this design encoded a lattice with an unequal distance between proteins in the *x*–*y* and *z* directions.

Negative-stained TEM imaging confirmed the lattice formation with micron-sized domains (Supplementary Figs. 27‒29). In situ SAXS analysis revealed that the double-layered and single-layered lattices shared similar features, with 1st peak was attributed to the in-plane spacing, *a*_DL,Octa_ ~57 nm (Fig. 4b and Supplementary Fig. 30). An indication of out-of-plane structure (*q* = 0.019 and 0.027 Å^−1^), attributed to the (111) and (211) planes was observed, which could only occur when at least two layers in the *z* direction were present. The SAXS analysis showed that proteins did not affect the DNA lattice integrity, while proteins occupied positions prescribed by the Octa framework (Fig. 4b, Supplementary Fig. 30). We further visualized assembled arrays in 3D using cryo-ET, as discussed above (Fig. 4d and Supplementary Figs. 31, 32). The final 3D maps (Fig. 4e, f) were resolved at a resolution of 156 Å (Fig. 4f and Supplementary Fig. 32) and confirmed the double-layered lattice, where the reconstructed array presented the top (blue) and bottom (orange) layers at 90° rotations. Octa connecting in the same plane by four vertices and with another layer via one vertex, as well as ferritin docking inside (black arrows in Supplementary Fig. 32d) agreed with our design and the SAXS results (Fig. 4b). A cryo-ET imaging reveals that a formed double-layer lattice (Fig. 4e, f) exhibits an improved orientational order over a single-layer lattice (Fig. 3e, f), which is attributed to an additional binding in *z* direction that stabilizes the octahedra orientations.

**Fig. 5:**
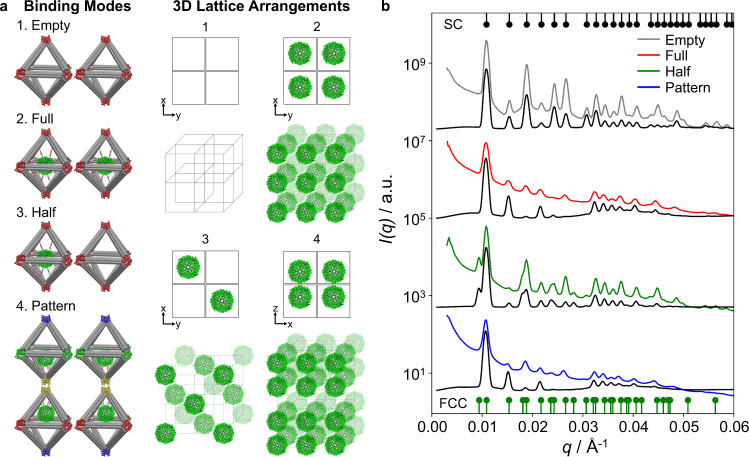
Design of 3D designed ferritin arrays and their in situ SAXS analyses. **a** Schematic view showing the 3D protein/Octa lattices formed by encoded Octa voxel. Depending on the activated vertices and the encoding sequences which facilitated a variety of binding modes, four different types (i.e., empty, full-filled, half-filled, and pattern) of unit cells of the 3D Octa and ferritin/Octa lattices were designed, as presented by the 2D projections and 3D views. **b** Experimental (colored curves) and simulated (black curves) *I*(*q*) profiles (shifted vertically for clarity) of the different types of 3D ferritin/Octa lattices. Gray (empty Octa, control) and red (full-filled ferritin/Octa) curves correspond to a simple cubic (SC) lattice, green (half-filled ferritin/Octa) curve relates to an face-centered cubic (FCC) lattice, and blue (pattern ferritin/Octa) curve corresponds to a tetragonal lattice, see text and “Methods” section for details. The 3D indices of a SC (bottom) and an FCC lattice (top) are indicated, respectively.

In the next step, we extended the presented protein assembly strategy for creating several types of designed 3D protein arrays. We programmed different color-encoded inter-vertex hybridizations of the protein voxels to achieve different 3D protein patterns, where four in-plane vertices bound to four *x*–*y* neighboring Octa and the out-of-plane vertices were engaged in layers in *z* direction. Specifically, a “one-color” system composed of two different Octa and one set of complementary DNA pairs (Fig. 5a and Supplementary Fig. 33), and a “four-color” system included four different Octa with four sets of complementary pairs (Fig. 5a and Supplementary Fig. 34). We designed three types of protein arrays: (i) a “full-filled” array that assembled one-colored protein voxels in a SC lattice (2 in Fig. 5a); (ii) a “half-filled” array that assembled alternating protein and empty one-colored voxels into a face-centered cubic (FCC) lattice (3 in Fig. 5a); and (iii) a “patterned” array that assembled the off-centered (Off2) protein voxels in the 3D lattice of commensurately stacked double-layers using a four-color scheme (4 in Fig. 5a). Similar to 2D lattices one-pot assembly was employed following several days of annealing and cooling.

First, we assembled and probed the one-color and four-color empty Octa 3D lattices (Fig. 5a), which shows a simple cubic (SC) lattice with the constants of 57.6 and 58.6 nm, respectively (Supplementary Figs. 35, 36 and 38)^66^. For the full-filled protein lattice (2 in Fig. 5a), SAXS revealed a high degree of long-range SC order (constant 58.3 nm), as indicated by at least 15 orders of resolution-limited Bragg peaks, and excellent agreement between the experimental and the modeled *I*(*q*) profiles (Fig. 5b and Supplementary Fig. 36). Compared to the 3D apoferritin/Octa and empty Octa lattices, incorporation of ferritin into the 3D lattices reduced intensities at *q* = 0.019, 0.025, and 0.027 Å^–1^, or the (111), (210), and (211) planes (Supplementary Figs. 37 and 38), due to its iron core.

Next, we realized 3D lattices with alternating “half-filled” and “patterned” designs, as shown in 3 and 4, respectively in Fig. 5a. In the half-filled array, every next-neighbor voxel in SC array was empty, thus, a face-centered cubic (FCC) was expected. Correspondingly, SAXS profile showed peak positions at ratios ${q}_{n}/{q}_{1}=1:\sqrt{4/3}:\sqrt{8/3}:\sqrt{11/3}:2$…, indicating FCC lattice with a lattice constant of 115.9 nm. The SAXS modeling supported this conclusion (Fig. 5b and Supplementary Figs. 36‒38).

Finally, we designed a 3D patterned lattice, in which a double-layer formed by protein voxels (Off2) were stacked in the *z* direction, facing each other similarly to the double-layer design. This arrangement would result in a lattice with a primitive tetragonal unit cell which was slightly altered from SC lattice due to only a few nm protein shift (Off2). Our SAXS experiment revealed a successful lattice formation. The detailed modeling, accounting for the ferritin shift, showed that relative peak intensities should be affected, as well as a small peak should appear at the low-*q* region at sufficiently large shifts (Supplementary Fig. 39). We indeed observed an intensity increase at *q* = 0.024 Å^–1^ ((210) plane) and no low-*q* peak appearance, which suggests a protein shift <3 nm in a tetragonal lattice.

**Fig. 6:**
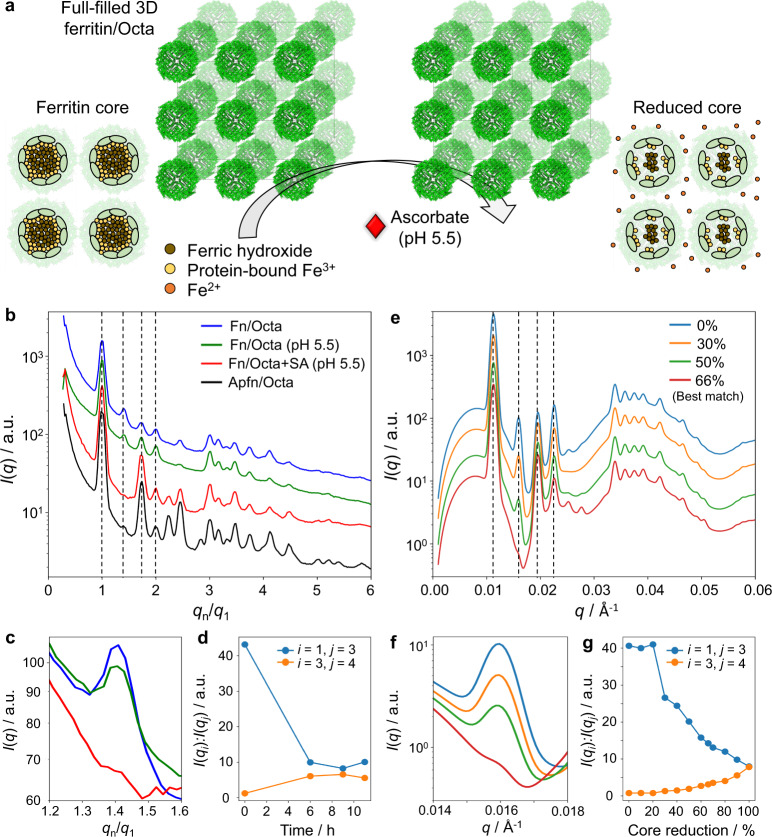
Conversion of ordered 3D (SC) ferritin array into an apoferritin array. **a** Schematic view showing the conversion of a 3D ferritin into apoferritin array by reduction using sodium ascorbate (SA), see text for details. **b** In situ SAXS analysis showing the conversion of 3D ferritin/Octa lattice (Fn/Octa, blue) into an apoferritin/Octa (Apfn/Octa) lattice after 9 h incubation with SA (red) solution containing ammonium acetate (NH_4_Ac, pH 5.5), and controls, Fn/Octa in NH_4_Ac (green) and a native Apfn/Octa lattice (black). Peak positions were normalized by the center of the 1st peak. The peaks that changed their relative intensities during conversion were shown by the dashed lines. Intensity profiles were shifted by a factor two for clarity. **c** The changes in the intensity of the 2nd SAXS peak (corresponded colors in **b**). **d** Analysis of the time-dependent study (Supplementary Fig. 43) showing relative peaks intensities between the 3rd and 4th (orange), and the 1st and 3rd (blue) peaks, indicating that the reduction process completed after 6 h. **e** Conversion of the ferritin array was analyzed by SAXS modeling (route 1 is shown, see text for details; intensities were shifted vertically for clarity by factor two), which indicated about 66% reduction of ferritin array. **f** SAXS modeling and analysis of the 2nd peak in **c** agreed with the experimental results. **g** Estimated degrees of core reduction based on SAXS modeling (see “Methods” section and Supplementary Figs. 44 and 45). Routes 2 and 3 for core dissolution pathways (Supplementary Figs. 46f and 47f), resulted in close values in the range of 60‒75% of reduced cores.

Following the assembly studies, we investigated the biological activity of ferritin in the assembled SC lattice. Sodium ascorbate (SA) was used to reduce ferric hydroxide of the mineral core^67,68^ and facilitated an iron release and conversion of the 3D ferritin array to an apoferritin array (Fig. 6a). To promote this process, an acidic environment was created by the addition of ammonium acetate (NH_4_Ac, 0.2 M, pH 5.5). A Ferrozine assay was first used to assess the release of iron ions from ferritin into solution, in which Fe^3+^ from the ferritin core was reduced by SA. The ferrous ions (Fe^2+^) complexed with Ferrozine were detected by UV-vis absorbance at 562 nm for ferritin but not apoferritin (Supplementary Fig. 40). We note that some formation Ferrozine-Fe^2+^ complex was detected from the ferritin sample in the acidic environment without SA, but a ~3× increase in absorbance was observed with SA (Supplementary Fig. 40). A majority of proteins remained intact under SA/NH_4_Ac reduction, as supported by DLS and TEM (Supplementary Fig. 41). To investigate the core dissolution of encapsulated ferritins, a form factor of single ferritin/Octa was analyzed by in situ SAXS after 5 h treatment with SA. The recovery of the two maxima in a form factor of protein voxels at *q* = 0.022 and 0.038 Å^–1^ indicated a reduced electron density of the iron core of the encapsulated ferritin, while TEM confirmed that proteins remained in Octa (Supplementary Fig. 42). Thus, we concluded that a ferritin voxel could be converted into an apoferritin voxel.

We applied a similar SA/NH_4_Ac reduction conversion approach to a 3D full-filled (SC) lattice (Fig. 6a). SAXS pattern, plotted as *q*_*n*_*/q*_1_, exhibited a change in relative peak intensities (dashed vertical lines in Fig. 6b, Supplementary Fig. 43), including a suppression of 2nd peak and enhancements of 3rd and 4th peaks (Fig. 6c, d), resembling scattering pattern of the apoferritin lattice. These changes stabilized after 6 h, suggesting that the reduction reaction had reached equilibrium (Fig. 6d). Overall, the lattice integrity was preserved, while its slight compaction (4.8% lattice constant decrease) was attributed to the charge interactions between NH_4_^+^ and negatively charged DNA backbone of Octa (Supplementary Fig. 43).

To estimate the degree of core reduction in the 3D ferritin array, SAXS profiles at different reaction times were compared with our models considering the size and electron density of the core-shell proteins (Supplementary Fig. 44). We simulated the biological core dissolution pathway^67,68^ by building three models that accounted for different mechanisms of iron core decrease: from the surface of the iron core (route 1), a uniform reduction of the core (route 2)^69^, and from the center of the core (route 3) (Fig. 6e and Supplementary Fig. 45). SAXS modeling for route 1 (Fig. 6e‒g), which is in excellent agreement with the experimental results (Fig. 6b‒d), estimated an iron decrease in the assembled ferritins by 66%, as indicated by the intensity reduction at *q*_2_ = 0.0159 Å^−1^ and increase at *q*_3_ = 0.0195 Å^−1^ and *q*_4_ = 0.0225 Å^−1^ during the conversion (dashed lines in Fig. 6e).

We further considered other iron release mechanisms, and applied SAXS modeling to their evolutions. In the absence of SA, all three pathways showed an estimated 20‒30% core reduction, likely due to a partial iron release from ferritin in the acidic environment (Supplementary Fig. 40). However, reduction of the ferritin cores was significantly increased to 60‒75% in the presence of SA, and the three modeled pathways yielded close values, which was 75% for route 2 and 61% for route 3 from the SAXS experiment (Fig. 6e and Supplementary Figs. 46, 47). Our SAX modeling of the three pathways also suggested core reduction was maximized at these values (i.e., 2nd peak reached a minimum, see Supplementary Figs. 45‒47 for details), which is consistent with our time-dependent study (Fig. 6d). Thus, the study shows that ferritins organized in a 3D lattice are able to release a significant amount of iron ions.

## Discussion

This work demonstrated an effective approach for the by-design assembly of 2D and 3D protein lattices with preserved biological activity. In specific realization, we encapsulated ferritin, a biologically functional iron storage protein, inside octahedral DNA origamis (Octa), creating protein voxels with tailored internal structure and external bonds. Such voxels can be programmed to assemble into desired lattice symmetries through the specific control of their coordination. The assembly of 2D single-layer, double-layer, and 3D lattices was demonstrated in our study, as confirmed by cryo-EM 3D imaging and in situ scattering methods. In a 3D array, ferritin was densely packed with a local concentration ~150× higher than the free proteins in the same solution. The stability, integrity, and “transparency” for molecular transport of ferritin 3D lattices allow to convert them into apoferritin lattices, which accompanied by a release of micromolar level of iron ions. Our work offers a versatile approach for designing and assembling targeted protein arrays with prospects of integrating diverse functional proteins into operational organized systems. Such an approach is anticipated to open the doors to active and regulated protein-based designed nanomaterials, which present intriguing possibilities for biomaterials, bio-catalysis, nanomedicine, nanotechnology, and cell-free biology.

## Methods

### Preparation of octahedral DNA origamis (Octa)

Octa was folded by mixing 20 nM of M13mp18 scaffold DNA and 100 nM of each staple oligonucleotides in TAE (1×) buffer containing 12.5 mM MgCl_2_ (TAE/MgCl_2_). The mixed solution was cooled from 90 °C to room temperature over 20 h to obtain target Octa. After synthesis, Octa were purified using the Amicon centrifugal filter units (100 kDa, Millipore Sigma) and centrifuged at 400 g and at 4 °C. Purification was repeated 6 times by adding fresh TAE/MgCl_2_ buffer in each cycle. Octa design and sequences are provided in Supplementary Fig. 1 and Supplementary Data 1‒3.

### Protein modification and encapsulation in single Octa

DNA grafting of ferritin and apoferritin was performed first by mixing the proteins (10 µM) with 0.75 mM azido-dPEG_8_-NHS ester in PBS (1×, pH 7.4) buffer and reacted for 3 h at room temperature or overnight (>12 h) at 4 °C. The azide-activated proteins were purified by Amicon centrifugal filter units (50 kDa, Millipore Sigma) and centrifuged at 3000 × *g* and 4 °C for 9 times. Next, 0.35 mM of DBCO-modified ssDNA (TATGAAGTGATGGATGAT/3DBCON/) was added to the azide-activated proteins (10 µM) in PBS and reacted overnight at 4 °C. The DNA grafted proteins were purified by filter units (100 kDa) and centrifuged at 3000 × *g* and 4 °C for 9 times. The protein products were concentrated to 10 µM and stored at 4 °C. UV-vis was used to estimate protein concentration and the number of ssDNA on protein surfaces (about 6‒7 ssDNA per ferritin), see Supplementary Fig. 4. To encapsulate protein in single Octa frames, Octa was mixed with the proteins (typically 40 nM for 30 nM Octa) in the TAE/MgCl_2_ buffer and the mixed solution was heated up to 48 °C and cooled to room temperature over 40‒60 h. Note that a lower starting temperature and shorter cooling time at higher temperatures should be considered depending on the protein stability.

### Negative-stained TEM imaging

In brief, Octa and protein/Octa (5 μL, 5–10 nM) solutions were dropped on a carbon film for 2 min and the residual liquid was removed with a piece of filter paper. After that, the grid was washed with 5 μL of deionized water followed by staining with 5 μL of 2 wt % uranyl acetate for 10 s. The excess liquid was removed with filter papers. TEM imaging was performed on a JEOL 1400 TEM, operating at 120 kV. The yields of protein encapsulation were obtained by direct counts from the TEM images. Among 394 counted apoferritin/Octa, 275 were well encapsulated and 119 were empty, yielding encapsulation of 69.8%; for ferritin/Octa, among 398 counted particles, 313 were well encapsulated and 85 were empty, yielding encapsulation of 78.6%.

### Small-angle X-ray scattering (SAXS)

#### Data acquisition and processing

Solution scattering data of free proteins, single Octa, and single protein/Octa was collected at the Life Sciences X-ray Scattering beamline (LiX, 16-ID) at the National Synchrotron Light Source II (NSLS-II), Brookhaven National Laboratory (BNL). Protein samples (0.5‒20 mg/mL) were prepared in PBS and Octa samples (10‒50 nM) were prepared in the TAE/MgCl_2_ buffer and loaded to the SAXS flow cells. The corresponded buffers were used as a reference and were subtracted from the samples. Scattering data of Octa and protein/Octa lattices were collected at the Complex Materials Scattering (CMS, 11-BM) beamline at the NSLS-II at BNL. The samples (typically 30 nM) were loaded into quartz capillary tubes and probed at the bottom of the capillary where samples precipitated. The 2D scattering images were converted into 1D scattering intensity profiles through azimuthal integration. All 1D data were subtracted to scattering intensity from the pure buffer before analysis.

#### SAXS modeling

SAXS modeling was primarily performed using the ScatterSim software package^61,66^, a python library for simulating 1D curves for the clusters and superlattices built from arbitrary anisotropic nanoscale objects. Detailed instrumentation, operation, and modeling are provided in the Supplementary Information.

### Cryogenic electron microscopy (cryo-EM)

#### Sample preparation and imaging

Ultrathin carbon (<3 nm) grids on lacey carbon support films (Ted Pella, Inc. #01824) were treated for 30 s in Fischione Nanoclean 1070 (70% power) with a mixture of Argon (75%) and Oxygen (25%). Cryo-EM samples were prepared in Vitrobot (Mark IV) at 19 °C (Octa) or 4 °C (apoferritin/Octa) with settings: relative humidity 100%, wait time 180 s, blot time 2 s, and blot force 4. Octa or apoferritin/Octa (3 μL) solution was incubated with the glow-discharged grids, blotted with filter paper, and then plunged into liquid ethane pre-cooled by liquid nitrogen. Prior to imaging, samples were transferred to a Gatan 626 cryo-specimen holder in liquid nitrogen and then inserted into the microscope. The specimen temperature was maintained at −170 °C during data collection. Cryo-EM imaging was performed on a Titan Halo TEM operating at 300 kV. Movies were recorded in the low-dose mode at ×28,500 magnifications on a Gatan K2 (1.15 Å /pix, 10 e^−^/Å^2^/s, 0.2 s/frame, 6 s total) or K3 (1.055 Å/pix, 20 e^−^/Å^2^/s, 0.05 s/frame, 3 s total) camera. All movies were saved in tif-compressed mode with dark gain correction only.

#### Image processing and 3D reconstruction

All data processing and reconstruction were performed in RELION3^70,71^, including motion correction, CTF estimation, particle picking. All image processing and 3D reconstruction were done on a 4-GPU Exxact Linux workstation or a 2-GPU Dell Linux workstation. 3D density map was displayed and manipulated in the UCSF Chimera package (10). We note that Octa using a single-stranded DNA scaffold to connect adjacent edges (six-helix bundle) might potentially contribute to the flexibility of the structure at each vertex. Such flexibility may affect or eliminate the octahedral symmetry and might result in a slightly off-centered position of apoferritin inside Octa, as shown in the reference-free 2D class images (Fig. 2g). Thus, we imposed no symmetry (C1 symmetry) throughout the entire data processing and 3D reconstruction. Detailed analyses and reconstruction procedures are provided in the Supplementary Information.

### Cryo-electron tomography (cryo-ET)

#### Sample preparation and imaging

Cryo-ET samples of single- and double-layered Octa and protein/Octa lattices were prepared on lacey carbon film grids (Cu-200LC, Electron Microscopy Sciences, and Cu-200LN, Pacific Grid-Tech) using the Leica EM GP plunge freezer (Leica, Inc.) at 90% humidity and 4 °C. In brief, lattice solutions (4 μL) were incubated with glow-discharged grids and rapidly plunged into liquid ethane after blotting. Samples were screened by Zeiss Libra 120 Plus TEM (Carl Zeiss SMT GmbH) with in-column energy filter and a 4 k × 4 k Gatan UltraScan 4000 CCD camera, and operating at 120 kV and a low-dose condition. High-resolution tilt series were acquired by a Titan Krios G2 TEM (ThermoFisher Scientific) with a Gatan energy filter (Gatan, Inc.), operating at 300 kV. Micrographs were recorded on a Gatan K3 direct electron detector operated in super-resolution mode at a nominal magnification of 53 K (1.46 Å/pixel) with defocus ~3 μm using SerialEM^72^. Non-tilt micrographs were acquired with 3.0 s exposure time, 0.15 s each frame, at a dose rate of ~8 e^−^/Å^2^/s. Tilt series were collected from −51° to +51° at a 3° increment, starting at +21°, with 1.0 s exposure time, 0.15 s each frame, at a dose rate of ~8 e^−^/Å^2^/s.

#### Image processing and 3D reconstruction

Anisotropic image motion of each frame of image stack in super-resolution mode (0.73 Å/pixel) was corrected by MotionCor2^73^. All tilt series were binned by two times (1.46 Å/pixel) and aligned by IMOD^74^. 3D density maps of the whole micrograph were aligned and reconstructed by IMOD, after the tilt series were binned eight times (11.68 Å/pixel). For high-resolution 3D structure, a tilt series of a focused area with ~336 × 336 nm^2^, i.e. 288 pixel × 11.68 Å/pixel, was windowed and extracted from the tilt series following the IPET reconstruction protocol^65^. All 3D maps were low-pass filtered to 80 Å using ENAN software^75^ and displayed in the UCSF Chimera package^76^. Detailed analyses and reconstruction procedures are provided in the Supplementary Information.

## Supplementary information

Supplementary Information

Supplementary Data 1

Supplementary Data 2

Supplementary Data 3

## Data Availability

The data supporting the findings of this study are available within this article and its Supplementary Information or from the corresponding author upon reasonable request. The Supplementary Information includes materials, supplementary figures, and detailed experimental methods, including gel electrophoresis, dynamic light scattering, ferrozine assay, cryo-EM imaging, single-particle reconstruction, individual particle electron tomography, small-angle X-ray scattering, and modeling.

## Code availability

The scripts used in SAXS analysis and modeling are included in the ScatterSim software package (https://github.com/CFN-softbio/ScatterSim), a Python package available for download through GitHub or from the corresponding author upon reasonable request.
