# Supplementary material for: Designed and biologically active protein lattices
Source: Nat Commun. 2021 Jun 17;12:3702. doi: 10.1038/s41467-021-23966-4 (PMC8211860; doi:10.1038/s41467-021-23966-4)
Supplement: Supplementary file 1 — Supplementary Information [file 41467_2021_23966_MOESM1_ESM.pdf]

Supplementary Information

Designed and Biologically Active Protein Lattices

Shih-Ting Wang[1], Brian Minevich[2], Jianfang Liu[3], Honghu Zhang[1], Dmytro Nykypanchuk[1], James Byrnes[4], Wu Liu[5], Lev Bershadsky[1], Qun Liu[5], Tong Wang[6], Gang Ren[3] and Oleg Gang[1,2,7*]

[1]Center for Functional Nanomaterials, Brookhaven National Laboratory, Brookhaven Avenue, Upton, New York, 11973, United States

[2]Department of Chemical Engineering, Columbia University, New York City, New York, 10027, United States

[3]The Molecular Foundry, Lawrence Berkeley National Laboratory, 1 Cyclotron Road, Berkeley, California, 94720, United States

[4]Energy Sciences Directorate/Photon Science Division, NSLS II, Brookhaven National Laboratory, Upton, New York, 11973, United States

[5]Biology Department, Brookhaven National Laboratory, Bell Avenue, Upton, New York, 11973, United States

[6]Advanced Science Research Center at the Graduate Center of the City University of New York, New York City, New York, 10031, United States

[7]Department of Applied Physics and Applied Mathematics, Columbia University, New York, NY, 10027, United States

**E-mail: og2226@columbia.edu*

Supplementary Figures

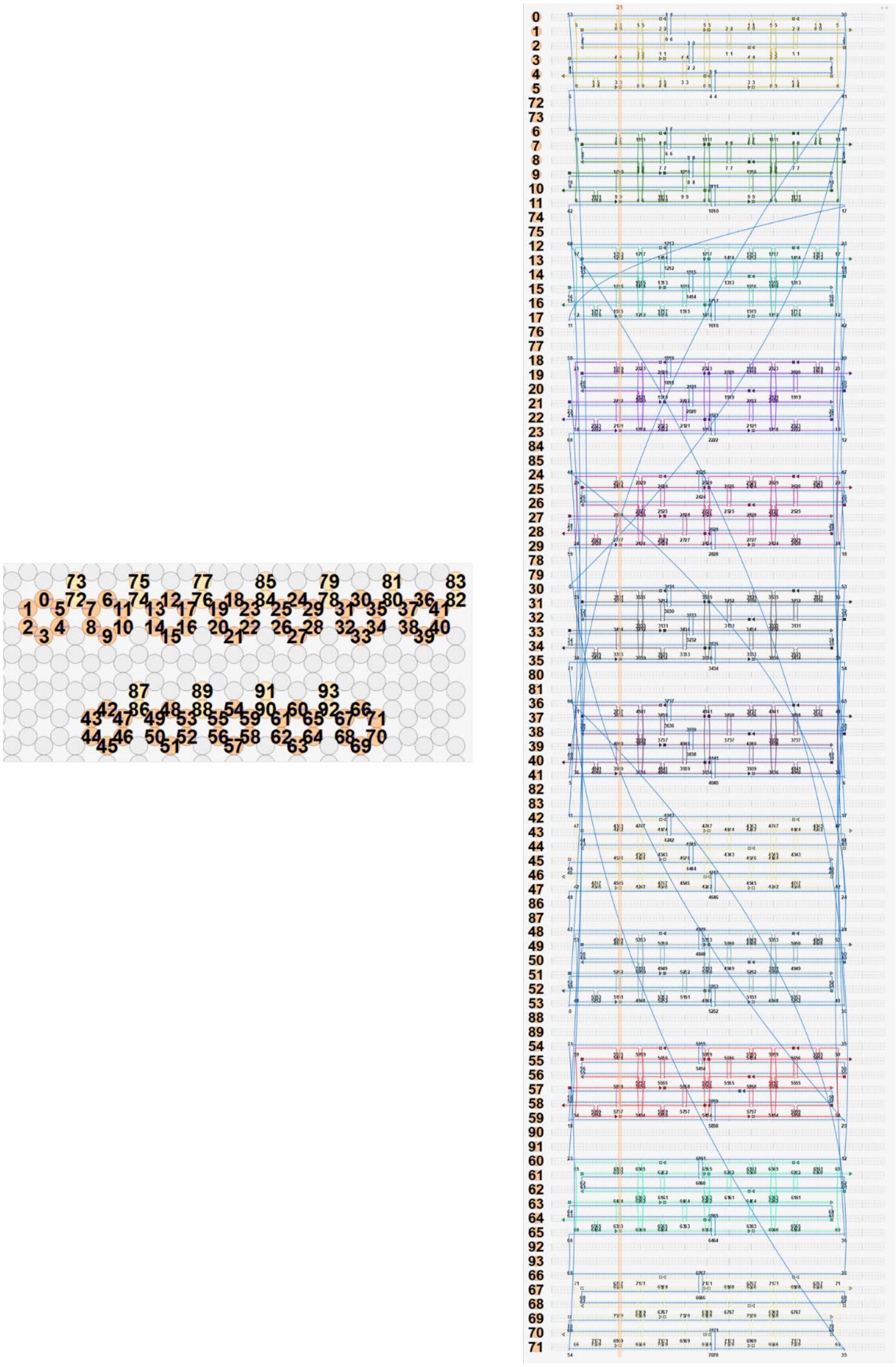

Supplementary Figure 1. Octa design in caDNAno (https://cadnano.org). Octa sequences are provided in Supplementary Data 1. The DNA sequences added to Octa for generating 2D and 3D lattices are provided in Supplementary Data 2 and 3, respectively.

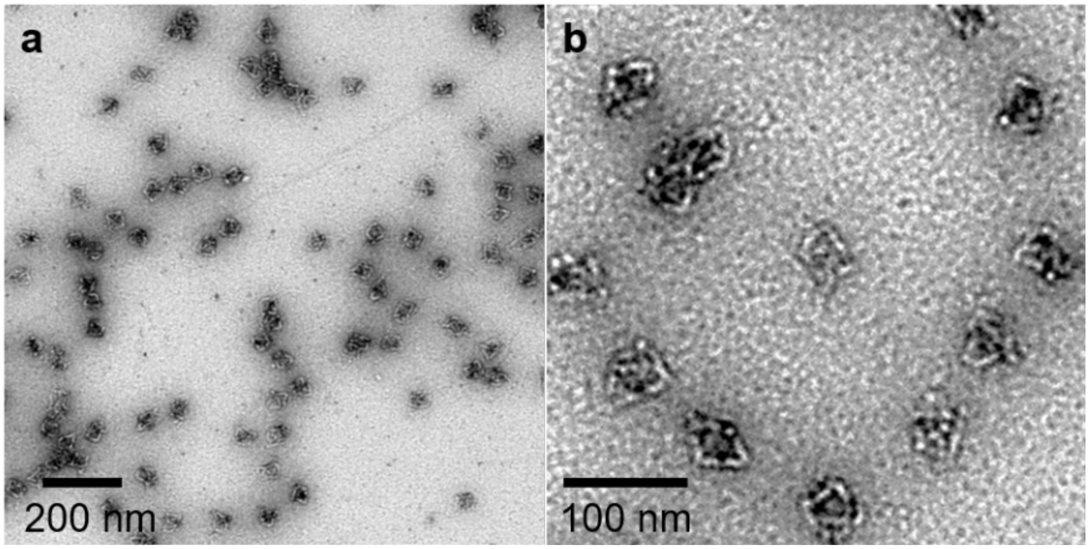

Supplementary Figure 2. Negative-stained TEM images of the octahedral DNA origami (Octa). b is a magnified image of the Octa structure.

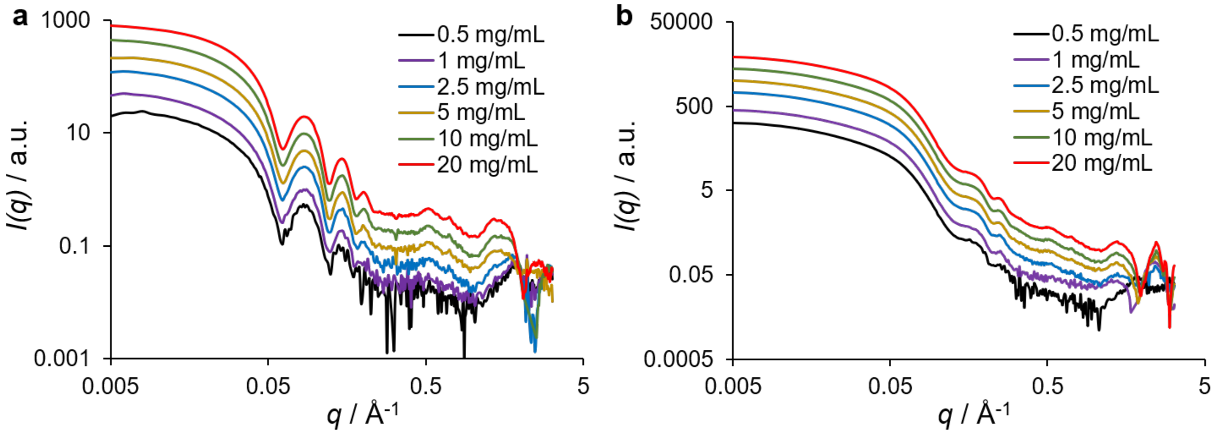

Supplementary Figure 3. SAXS intensity profiles of different concentrations of a, apoferritin and b, ferritin in PBS buffer.

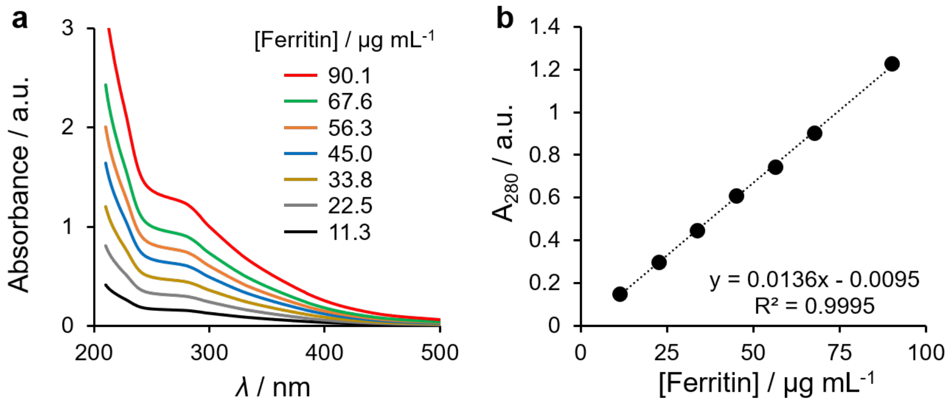

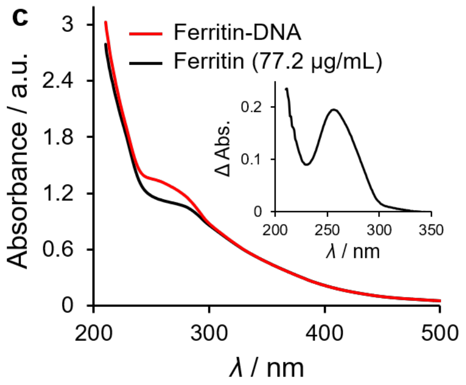

Beer-Lambert Eq.: $A = \varepsilon \cdot l \cdot C$

Parameter	Value
DNA extinction coefficient (ε)	189200 L/(mol · cm)
DNA molecular weight	5617.7 g/mol
Ferritin molecular weight	20.21*24 kDa<br>(PDB ID: 1IER)

Supplementary Figure 4. UV-vis quantification of protein concentration and ssDNA grafting on protein surfaces. In a and b, UV-vis spectra of a range of known ferritin concentrations in PBS were measured where the absorbance at 280 nm (A_{280}) exhibited a linear relation. The standard spectra (example in c) were compared with those of ferritin-DNA and to obtain the absorption of ssDNA at 260 nm by subtraction. Using the Beer-Lambert equation and the known parameters, about 6–7 ssDNA per ferritin was estimated based on three independent measurements.

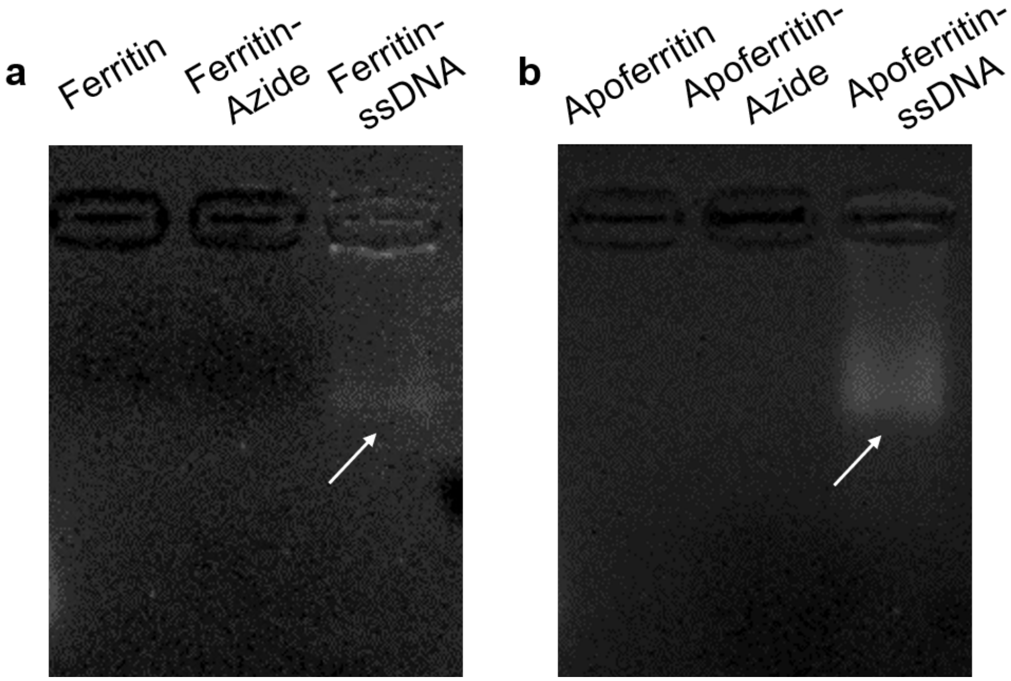

Supplementary Figure 5. AGE analyses of the two-step modification of a, ferritin and b, apoferritin. Protein surfaces were first activated with azide through the NHS-amine reaction and followed by conjugation of the DBCO-modified ssDNA *via* click chemistry. The white arrows pointed out the bands of the ssDNA-modified proteins. AGE was performed with 4 wt% agarose (medium EEO) at 80 V.

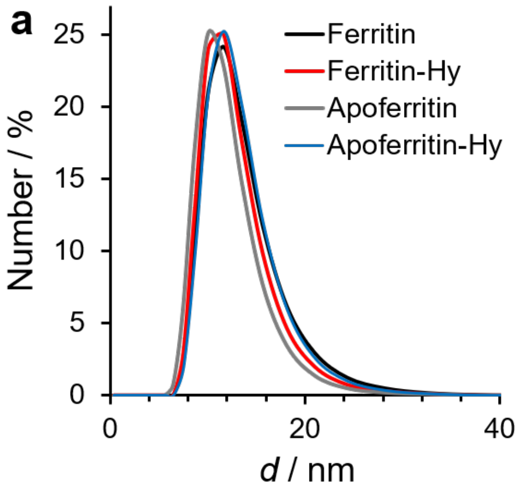

b

Sample	*d* (avg.) / nm
Ferritin	12.7 ± 0.7
Ferritin-Hy	12.2 ± 0.7
Apoferritin	11.5 ± 0.7
Apoferritin-Hy	12.7 ± 0.7

Supplementary Figure 6. a, DLS of ferritin, apoferritin and the ssDNA-modified proteins and b, a table showing the average sizes of the protein samples. The protein samples (100 nM) were diluted in PBS buffer and equilibrated for 120 s prior to measurement.

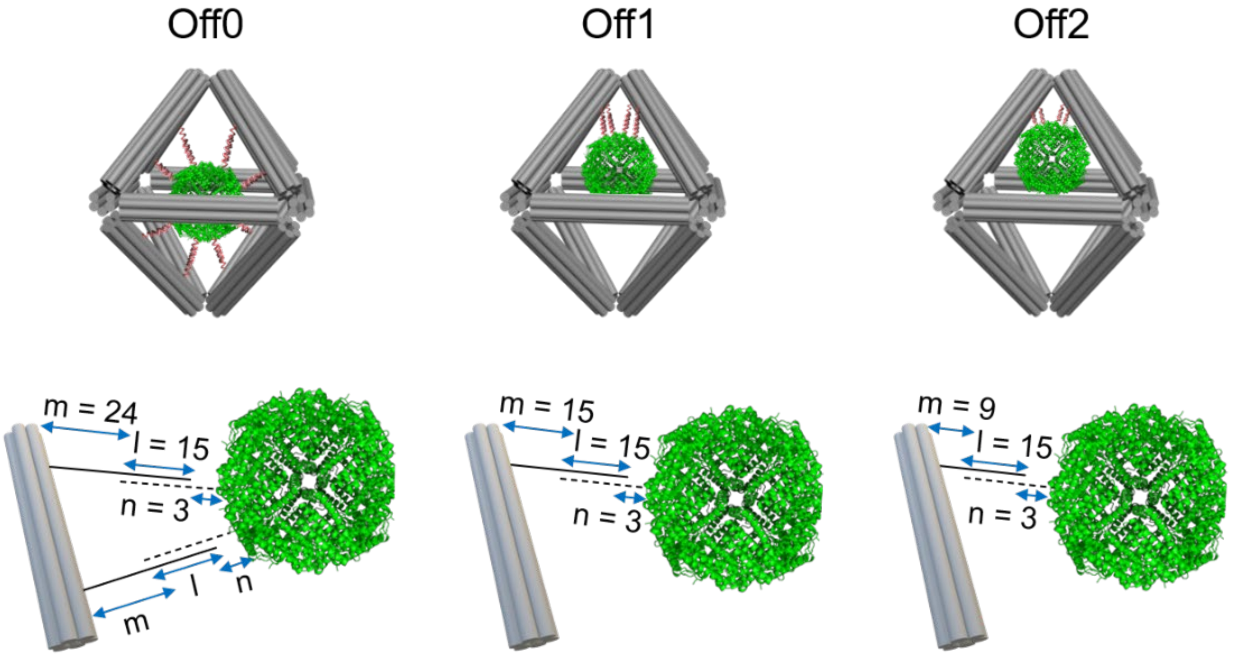

Supplementary Figure 7. Schematic view showing the lengths of DNA linking motifs used for protein encapsulation and to place the proteins (ferritin PDB ID: 1IER) at different positions inside the Octa structure. In the centered design (Off0), eight DNA linkers from the four select in-plane bundles were used; In the off-centered designs (Off1 and Off2), the four ssDNA linkers were extended from the same positions of the four select bundles converged at one vertex, and these strand positions (OC-staples-111 and 115, see Supplementary Data 1) were also used in Off0. *m* is the extended ssDNA from the bundle, *n* is the extended ssDNA from the protein, and *l* is the complementary bases that hybridize between the strands on Octa and protein.

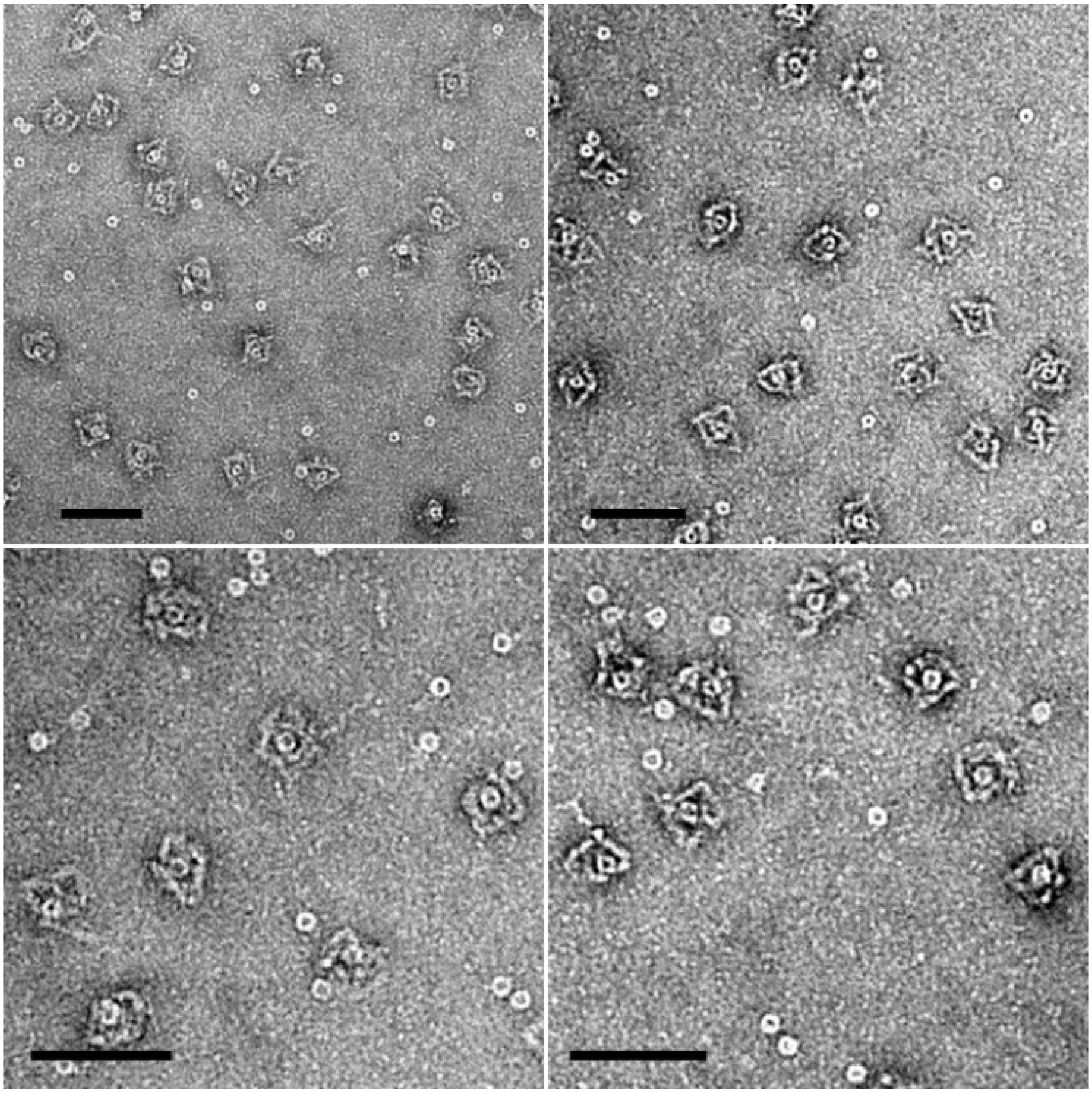

Supplementary Figure 8. Negative-stained TEM images of ferritin encapsulated inside Octa (scale bars: 100 nm).

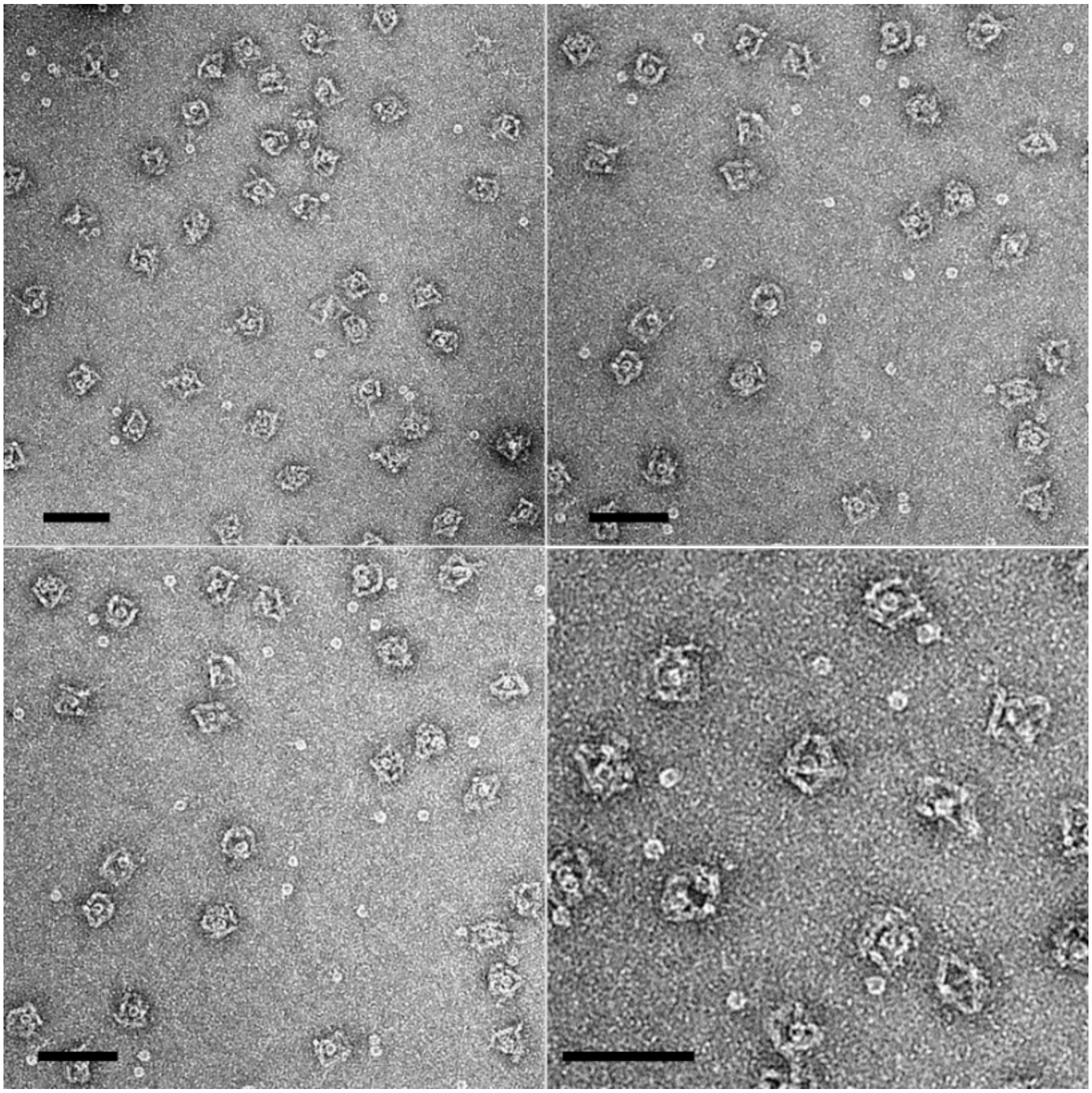

Supplementary Figure 9. Negative-stained TEM images of apoferritin encapsulated inside Octa (scale bars: 100 nm).

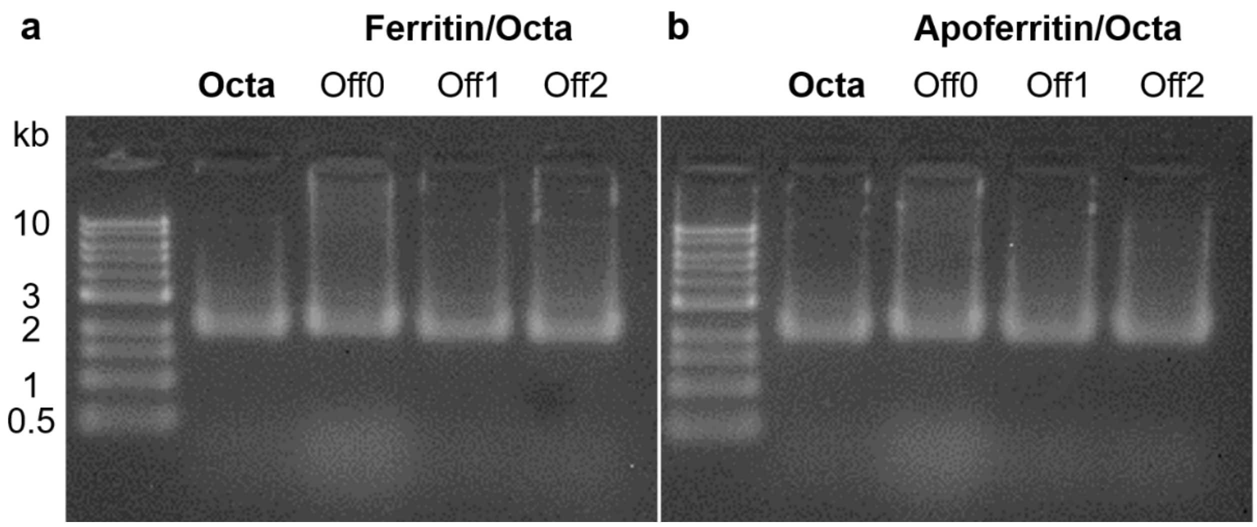

Supplementary Figure 10. AGE of a, ferritin/Octa (10 nM) and b, apoferritin/Octa (10 nM), where the encapsulated proteins were prescribed at different positions (center: Off0 and off-center: Off1 and Off2) inside the Octa structure. The small change of the electrophoretic mobility after protein encapsulation was likely due to the higher net charge and size of Octa compared to the proteins.

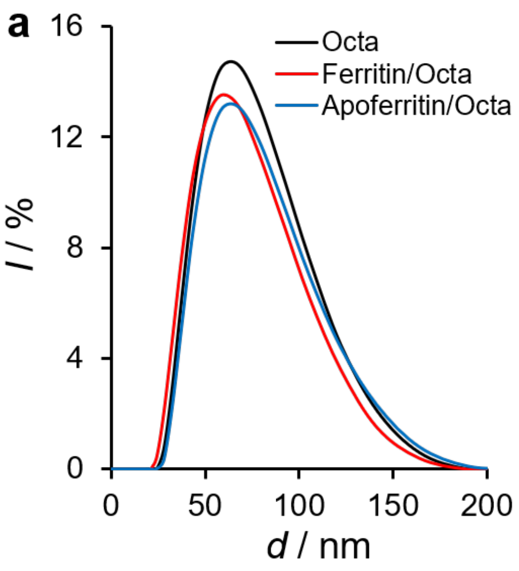

b

Sample	*d* (avg.) / nm
Octa	67.8 ± 5.3
Ferritin/Octa	60.3 ± 0.7
Apoferritin/Octa	75.2 ± 2.4

Supplementary Figure 11. a, DLS of Octa, ferritin/Octa and apoferritin/Octa, and b, a table showing the average sizes of the protein samples. The Octa samples were diluted to 5 nM in TAE (1X) buffer containing 12.5 mM $MgCl_2$ and equilibrated for 120 s prior to measurement.

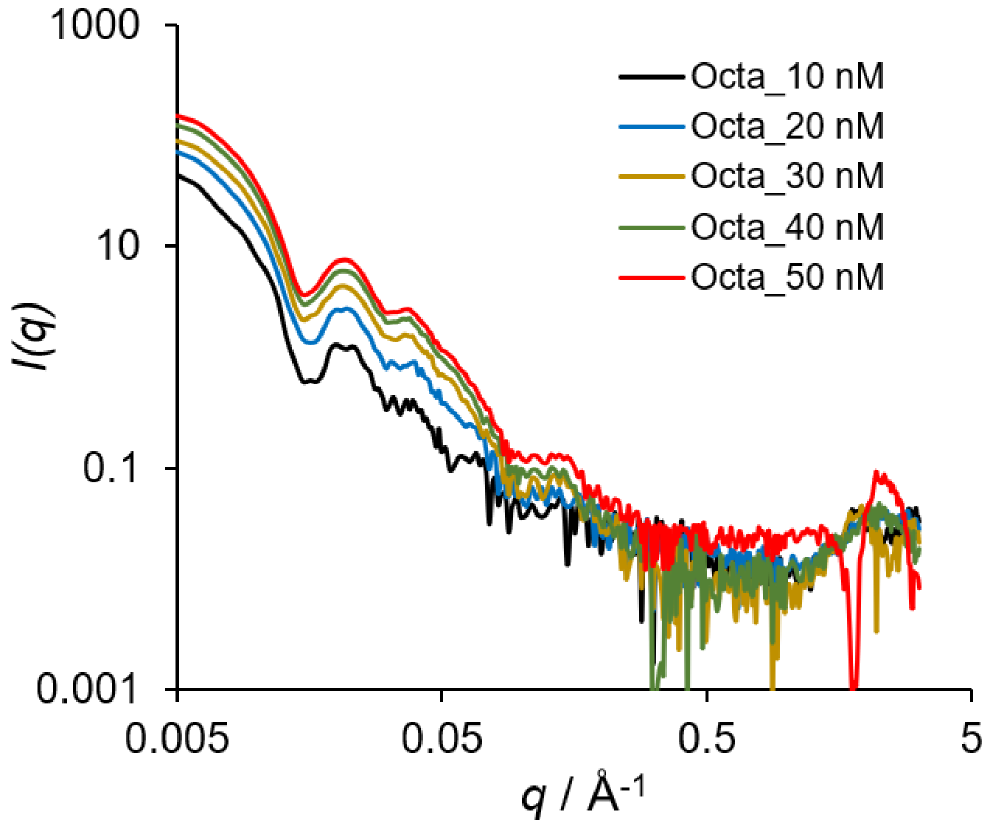

Supplementary Figure 12. SAXS intensity profiles of different concentrations of Octa in TAE (1X) buffer containing 12.5 mM $MgCl_2$.

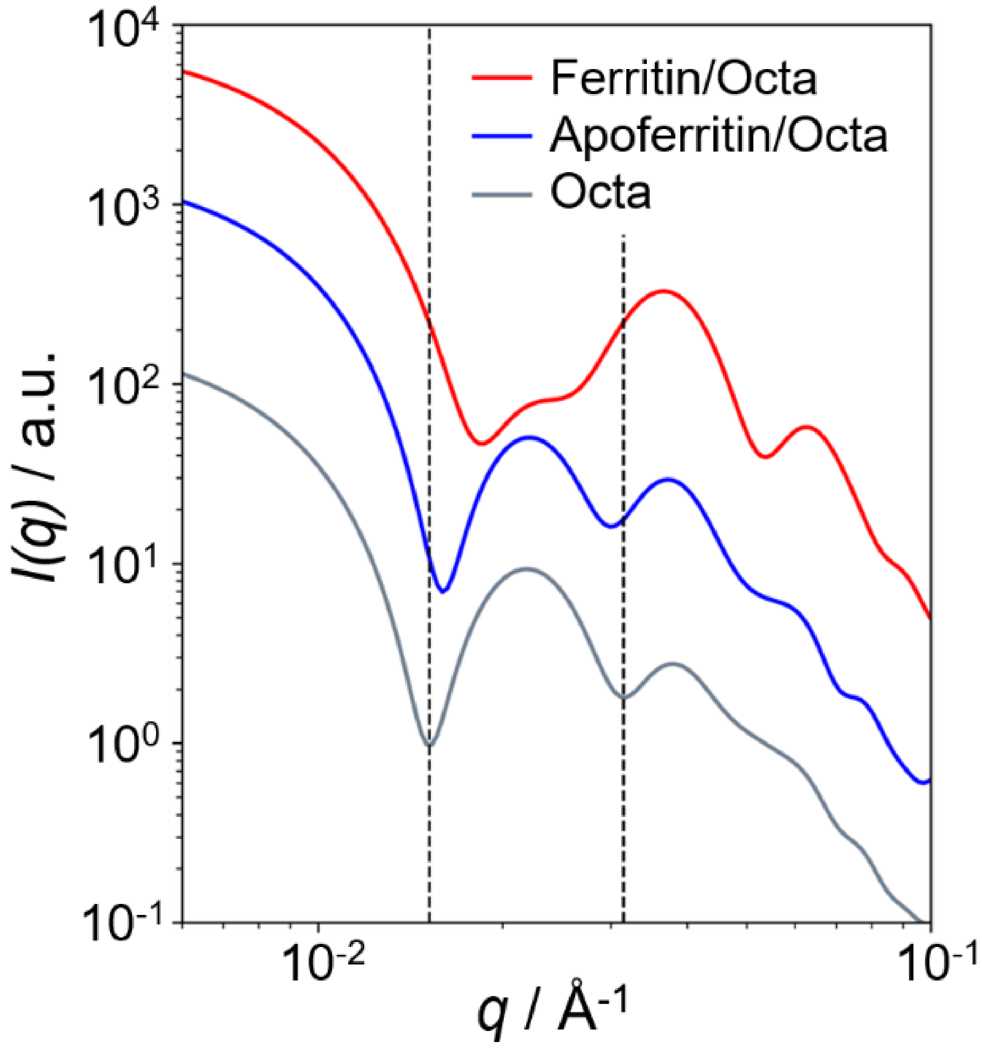

Supplementary Figure 13. SAXS analysis showing the simulated SAXS data of single empty Octa, ferritin/Octa and apoferritin/Octa, in which the scattering profiles showed a similar trend to the results obtained experimentally (Fig. 2f). In these simulated results, proteins were assumed to be 100% encapsulated inside Octa.

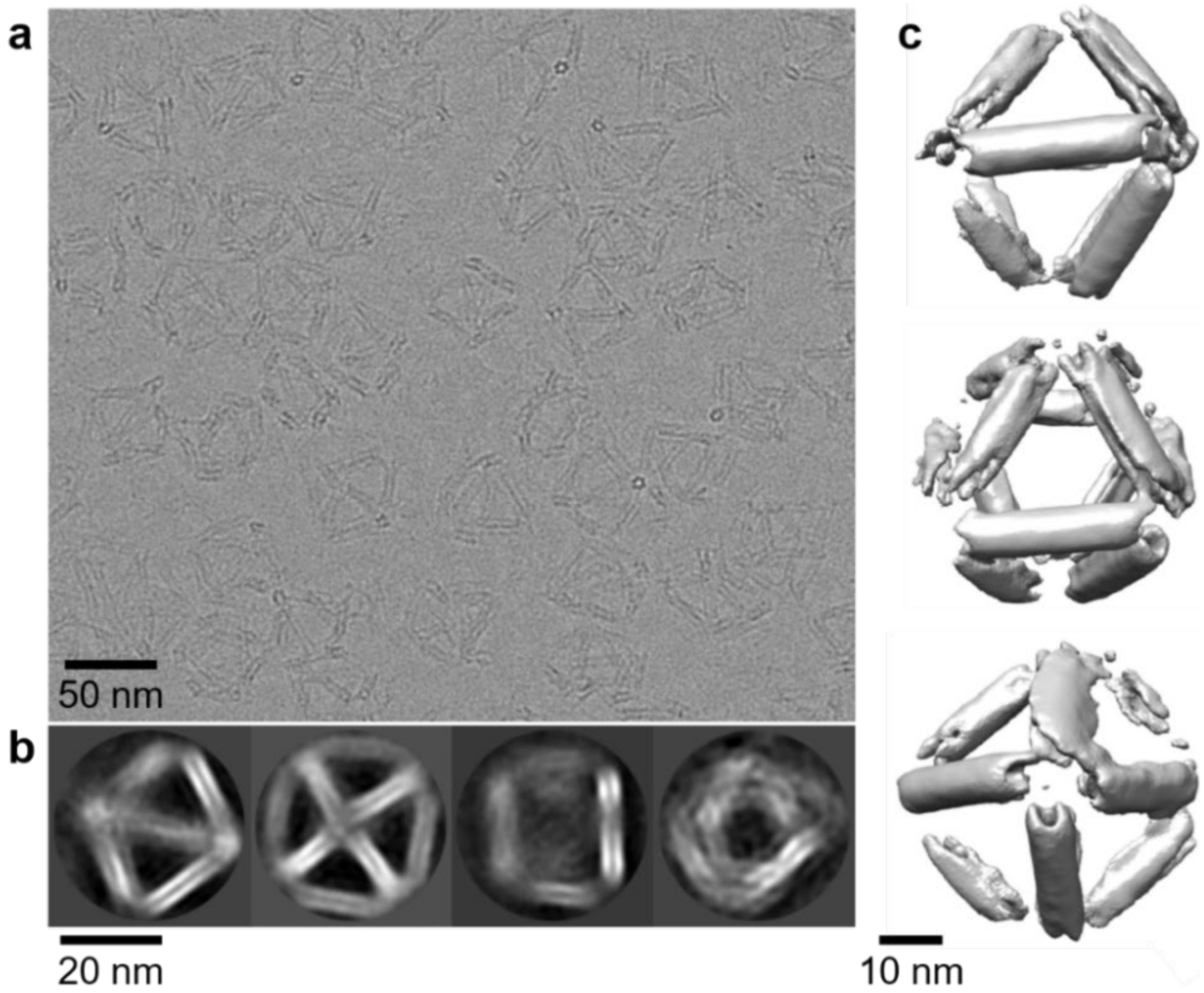

Supplementary Figure 14. Cryo-EM and single particle reconstruction of the structure of empty Octa. a, A cryo-EM micrograph after motion correction. b, Reference-free 2D class averages showing the representative views of Octa. c, 3D density maps of Octa viewed from the two-fold (left), three-fold (middle) and four-fold (right).

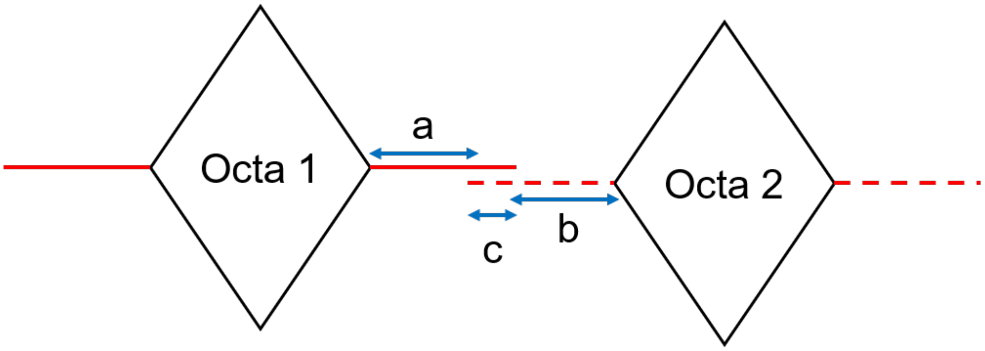

Supplementary Figure 15. Schematic view showing the inter-vertex linking motifs used for generating the 2D single-layered Octa and protein/Octa lattices in Fig. 3. The red solid lines and dashed lines extended from two Octa represent the in-plane complementary DNA pair in the one-colored system. a (=22) is a poly T part from Octa 1 vertex, b (=22) is the poly T part from the Octa 2 vertex, and c (=8) is composed of complementary bases that hybridize between strands.

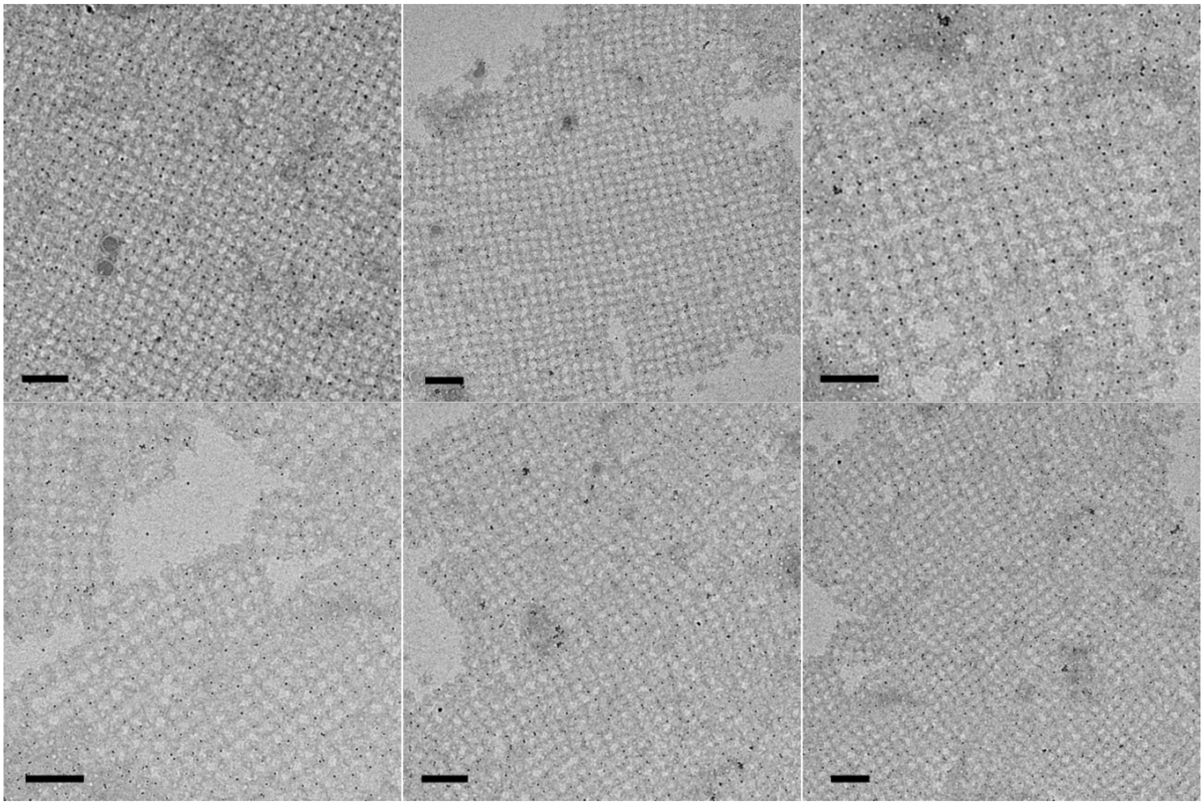

Supplementary Figure 16. Negative-stained TEM images of the 2D single-layered ferritin/Octa lattices. The globular structures (black) are ferritins (scale bars: 200 nm).

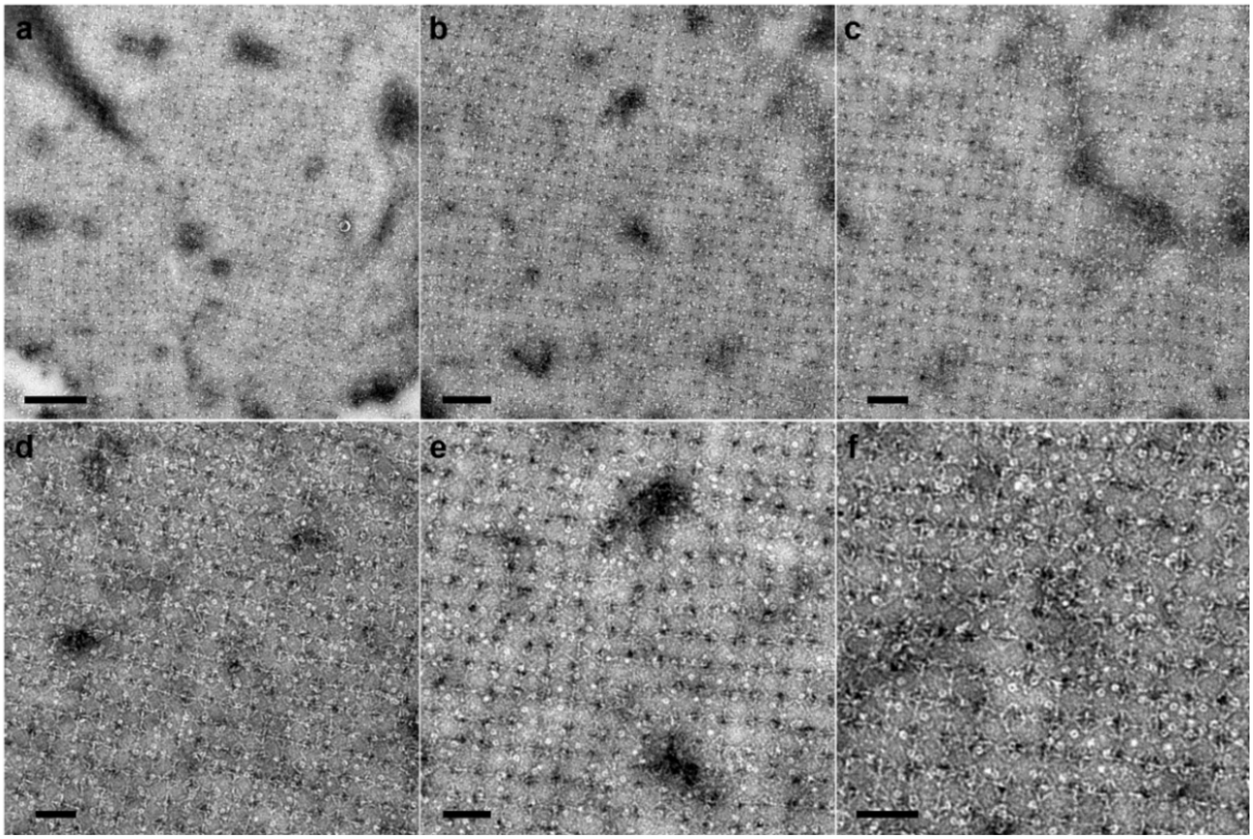

Supplementary Figure 17. Negative-stained TEM images of the 2D single-layered apoferritin/Octa lattices (scale bars: a-c, 200 nm and d-f, 100 nm).

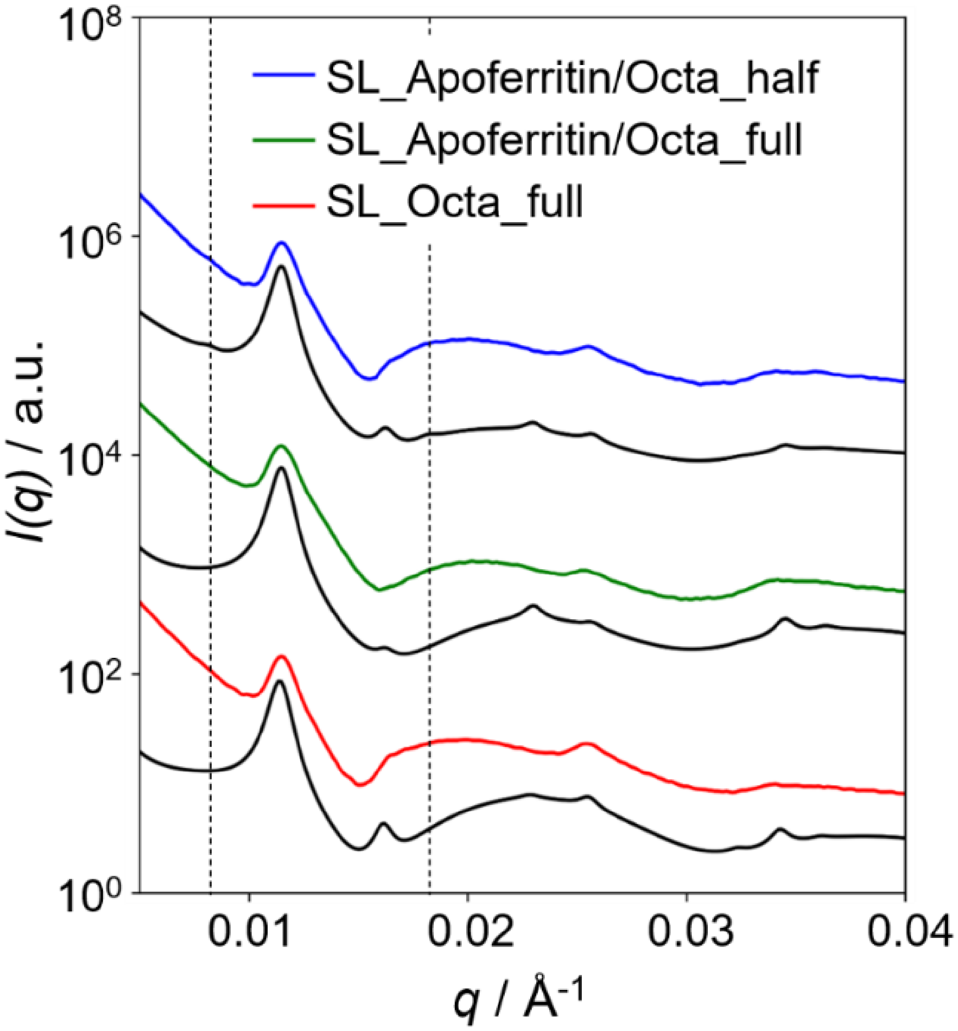

Supplementary Figure 18. SAXS analyses showing experimental data (colored lines) and simulated results (black lines) of the 2D single-layered empty Octa, full-filled apoferritin/Octa, and half-filled apoferritin/Octa.

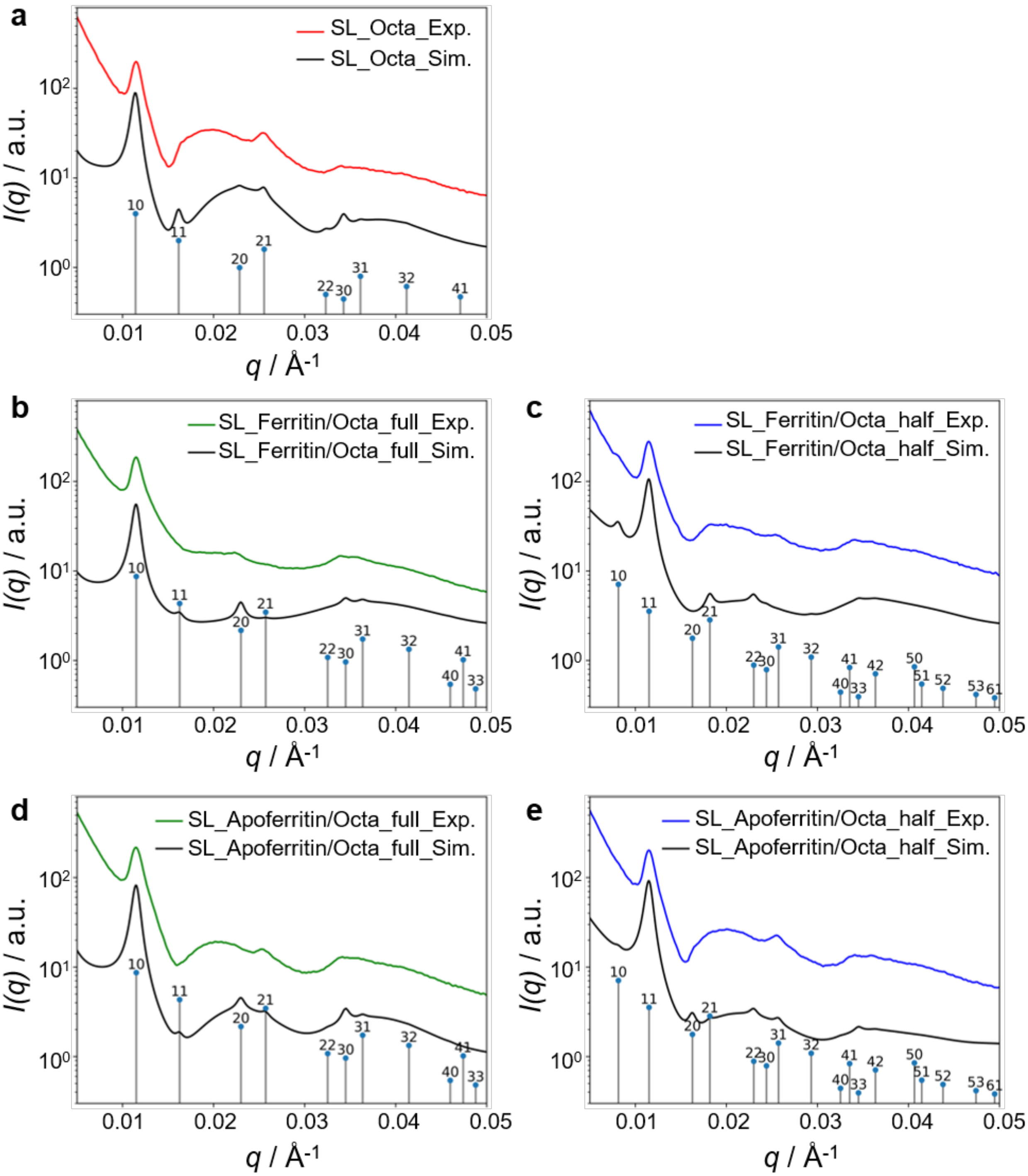

Supplementary Figure 19. SAXS analyses showing the experimental data (colored lines) and simulated results (black lines) of the 2D single-layered a, empty Octa, b, full-filled ferritin/Octa, c half-filled ferritin/Octa, d, full-filled apoferritin/Octa and e, half-filled apoferritin/Octa. For each simulated curve, indices of the corresponding scattering peaks are shown.

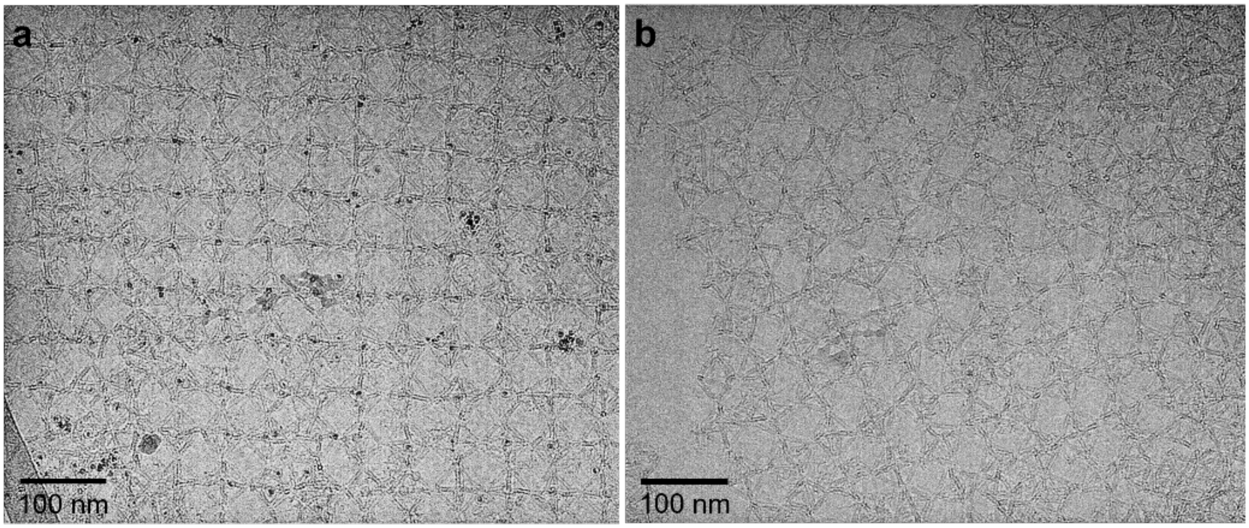

Supplementary Figure 20. Cryo-EM images of 2D single-layered a, ferritin/Octa lattices and b, empty Octa.

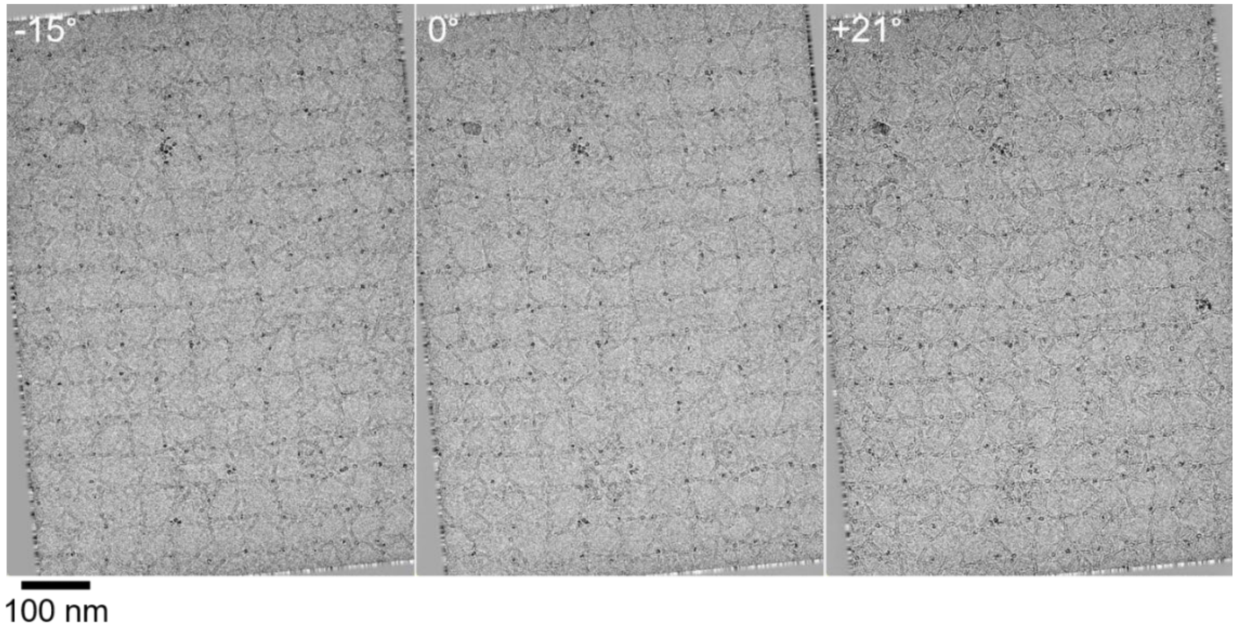

Supplementary Figure 21. Representative images of Cryo-ET tilt series of the single-layered 2D ferritin/Octa lattice. The single-layered lattice structure and the integration of ferritin into the structure were confirmed at the selected tilted angles.

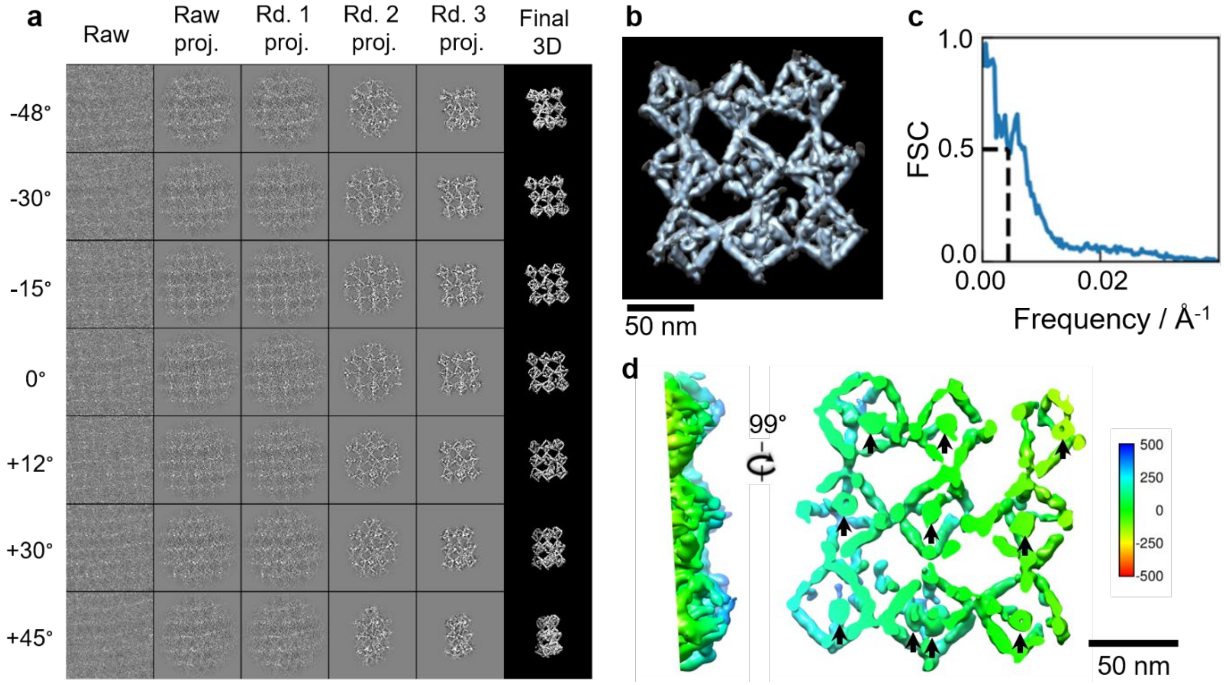

Supplementary Figure 22. IPET 3D reconstruction processes of the single-layered ferritin/Octa lattice. a, Seven representative tilt images of the select area of a ferritin/Octa lattice. Nine single ferritin/Octa in the select area are displayed in the first column from the left. Using IPET, the tilt mages are aligned to a common center *via* iterative refinements. The projections of raw, intermediate and final 3D reconstructions at the corresponding tilt angles are displayed in the next five columns. b, The final 3D density map. c, FSC analyses showing the resolution of the final 3D density map, which was estimated to be ~225 Å. d, A colormap for Fig. 3f showing the overlay of the encapsulated ferritin with the single-layered lattice in one structure. The black arrows indicate the protein positions in each Octa in the lattice. The color bars indicate Z height (unit: Å).

a

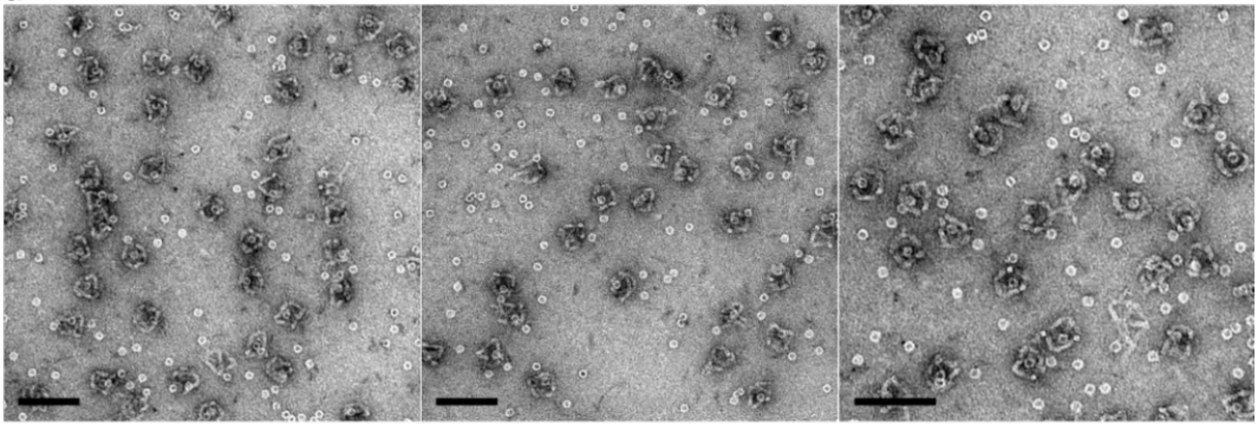

b

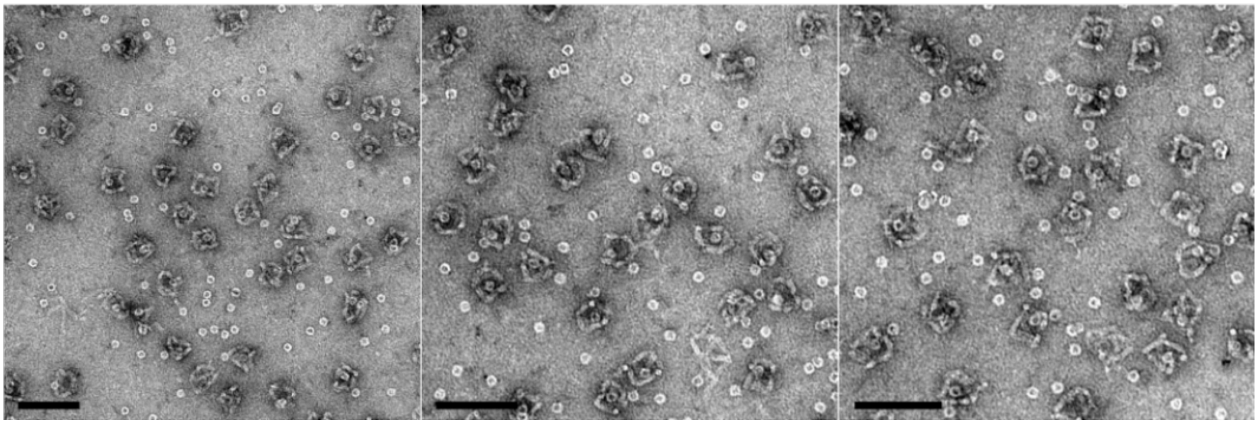

Supplementary Figure 23. Negative-stained TEM images of ferritin encapsulation at the off-centered positions inside Octa (a, ferritin/Octa_Off1; b, apoferritin/Octa_Off2). Scale bars: 100 nm.

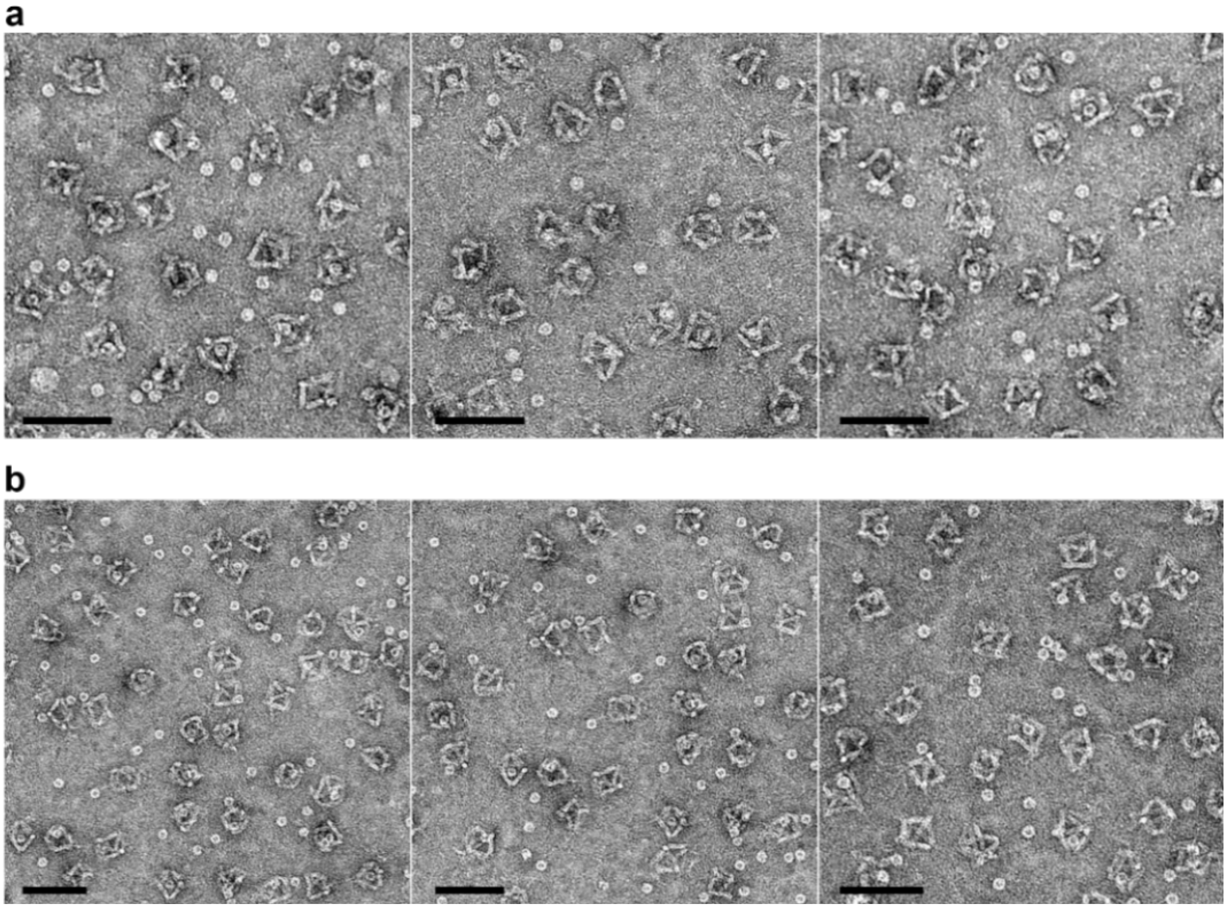

Supplementary Figure 24. Negative-stained TEM images of apoferritin encapsulation at the off-centered positions inside Octa (a, apoferritin/Octa_Off1; b, apoferritin/Octa_Off2). Scale bars: 100 nm.

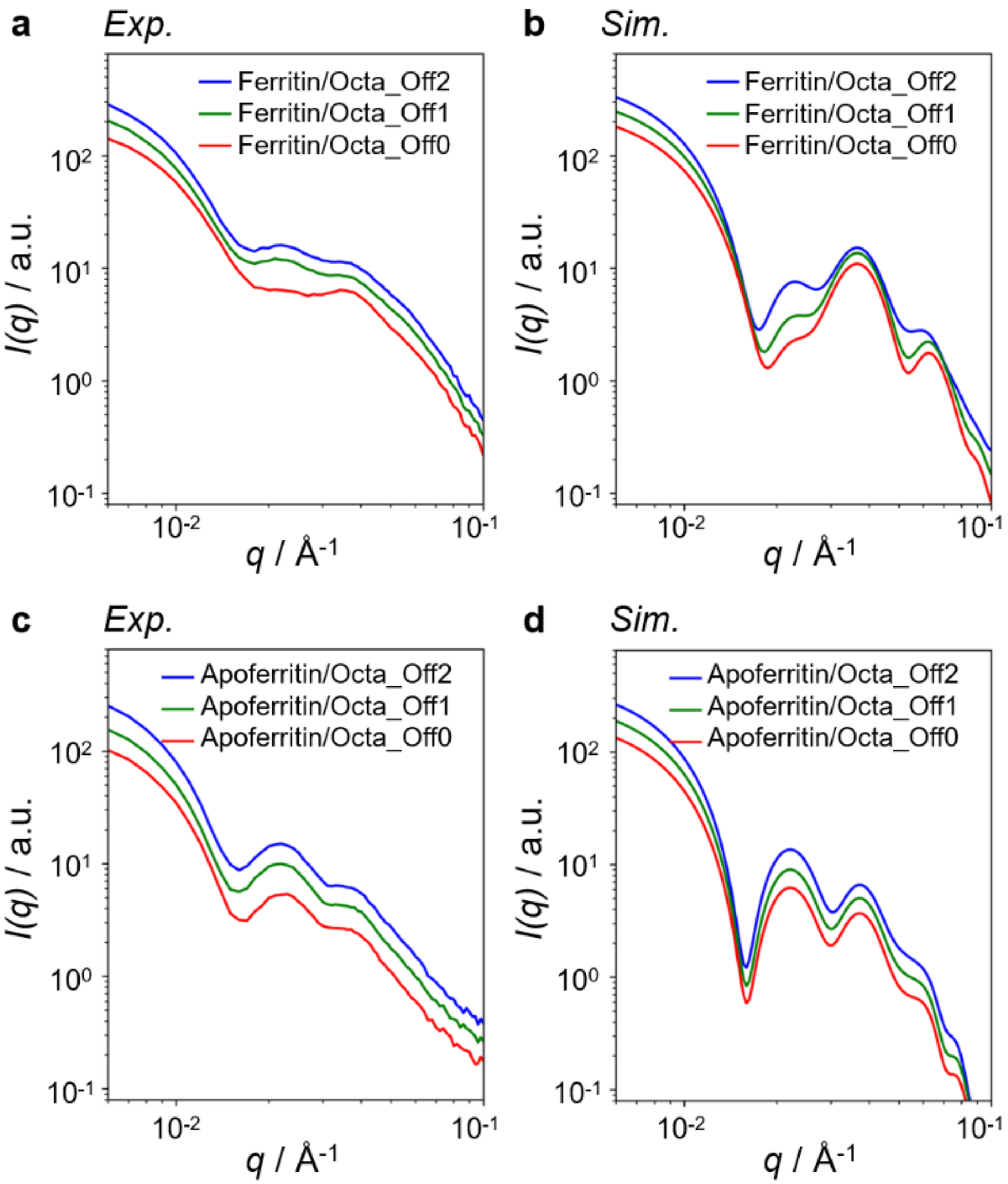

Supplementary Figure 25. SAXS analyses of a, b, single ferritin/Octa and c, d, single apoferritin/Octa, where the proteins were positioned at the center (Off0) or off-center (Off1 and Off2, Supplementary Fig. 7) inside Octa. a, c show the experimental data, and b,d are the simulated results. In these simulated results, proteins were assumed to be 100% encapsulated inside Octa. The plots in each penal are vertically shifted for display purpose.

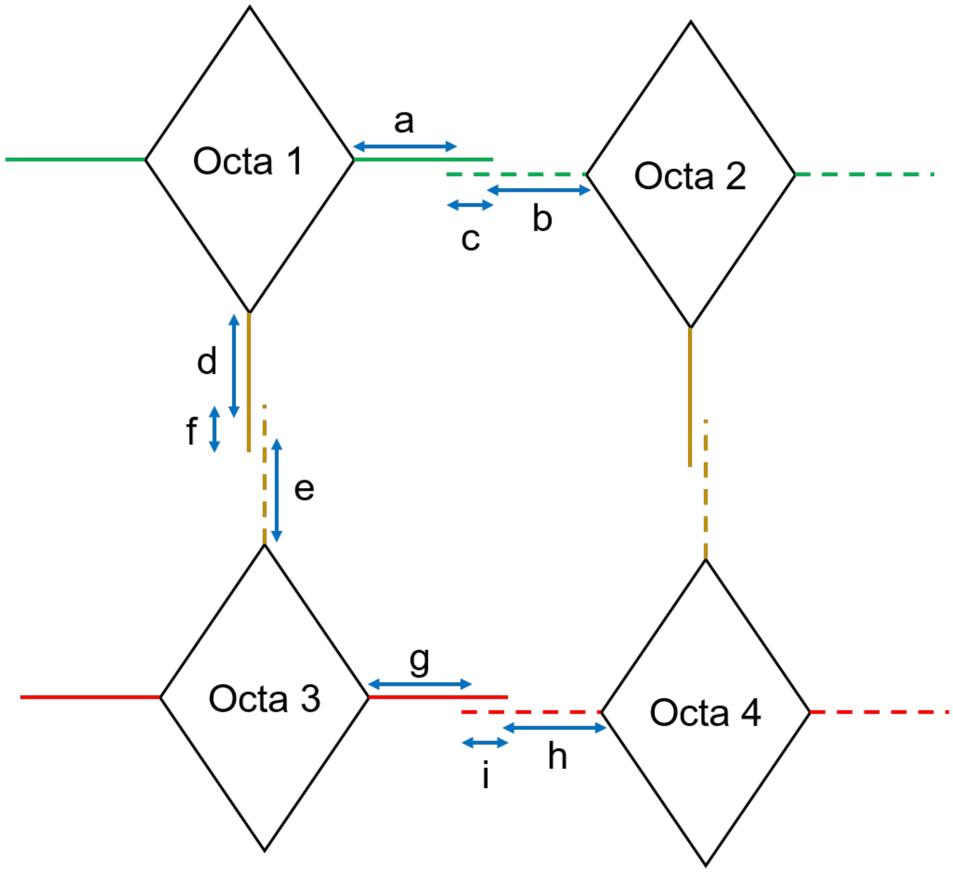

Supplementary Figure 26. Schematic view showing the inter-vertex linking motifs used for generating the 2D double-layered Octa and protein/Octa lattices in Fig. 4. The solid lines and dashed lines with matching colors extended from two Octa represent three sets (two in-plane and one out-of-plane) of the complementary DNA pairs in the three-colored vertex encoding system. a, b, d, e, g, h (=22) are the poly T parts from Octa vertices, and c, f and i (=8 or 12) are composed of different complementary bases that hybridize between strands.

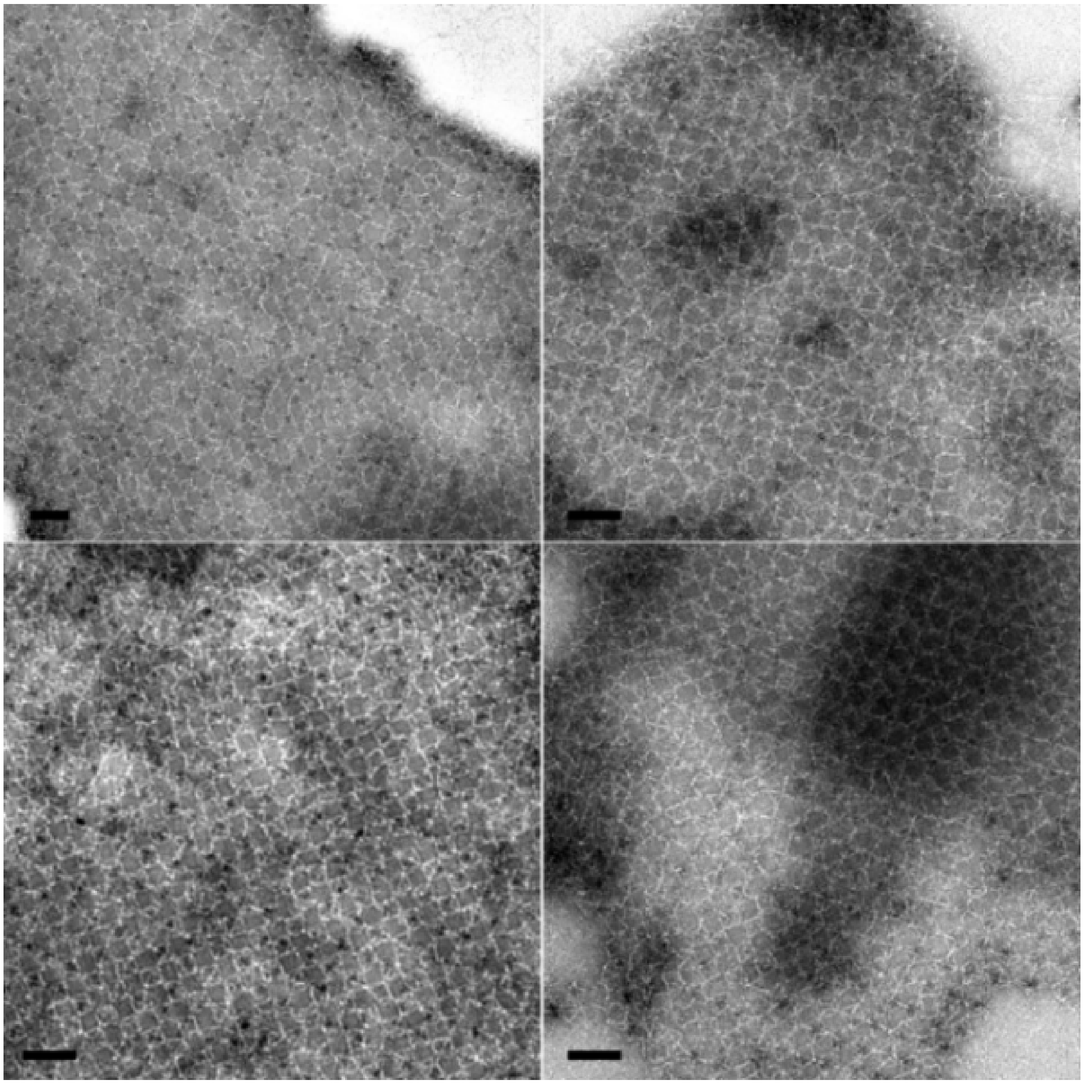

Supplementary Figure 27. Negative-stained TEM images of 2D double-layered Octa lattice (scale bars: 100 nm).

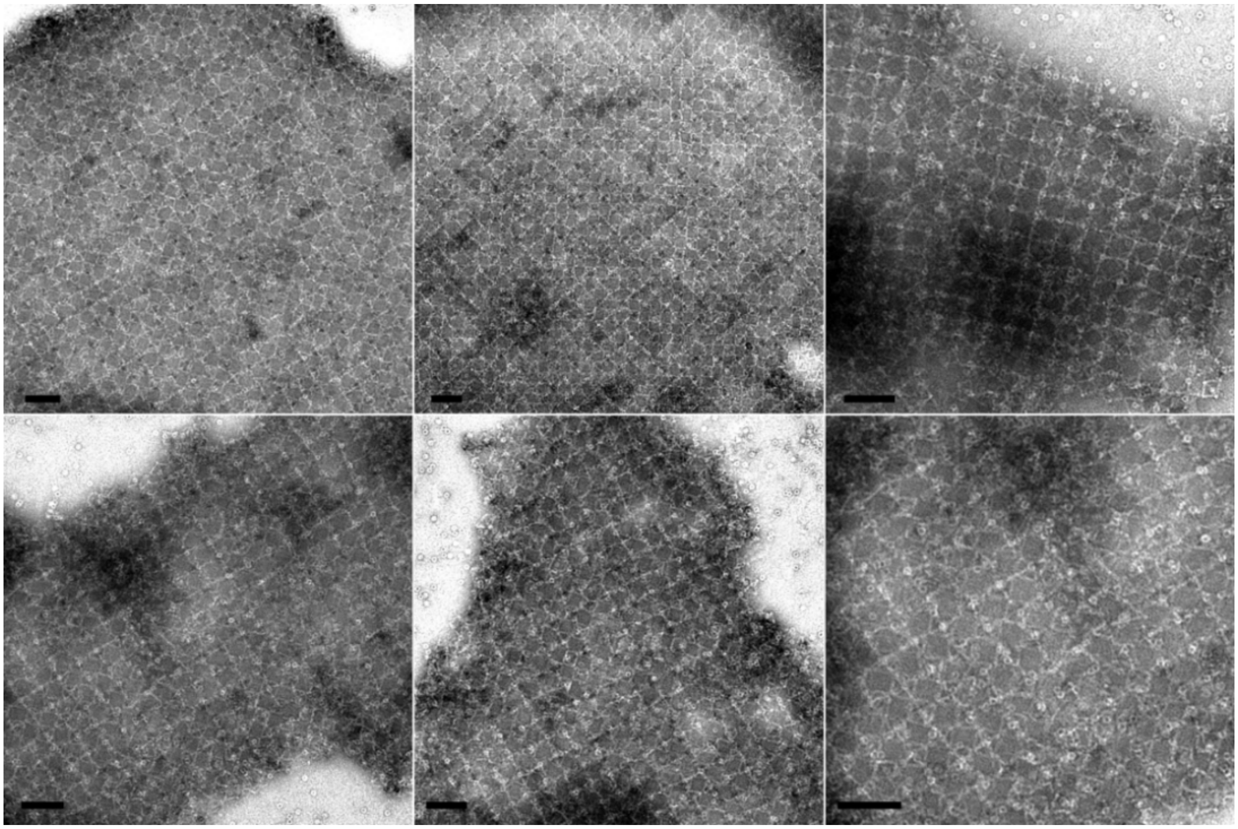

Supplementary Figure 28. Negative-stained TEM images of 2D double-layered ferritin/Octa lattice (scale bars: 100 nm).

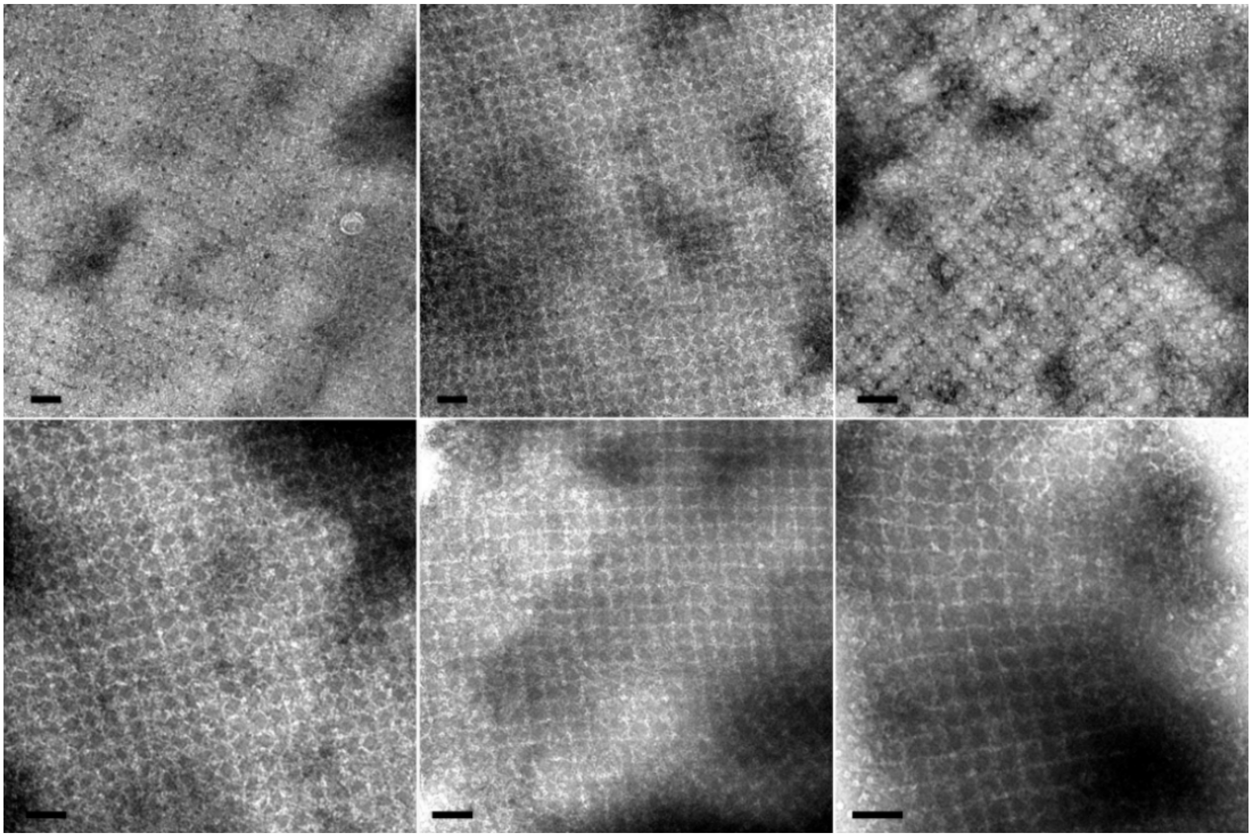

Supplementary Figure 29. Negative-stained TEM images of the 2D double-layered apoferritin/Octa lattice (scale bars: 100 nm).

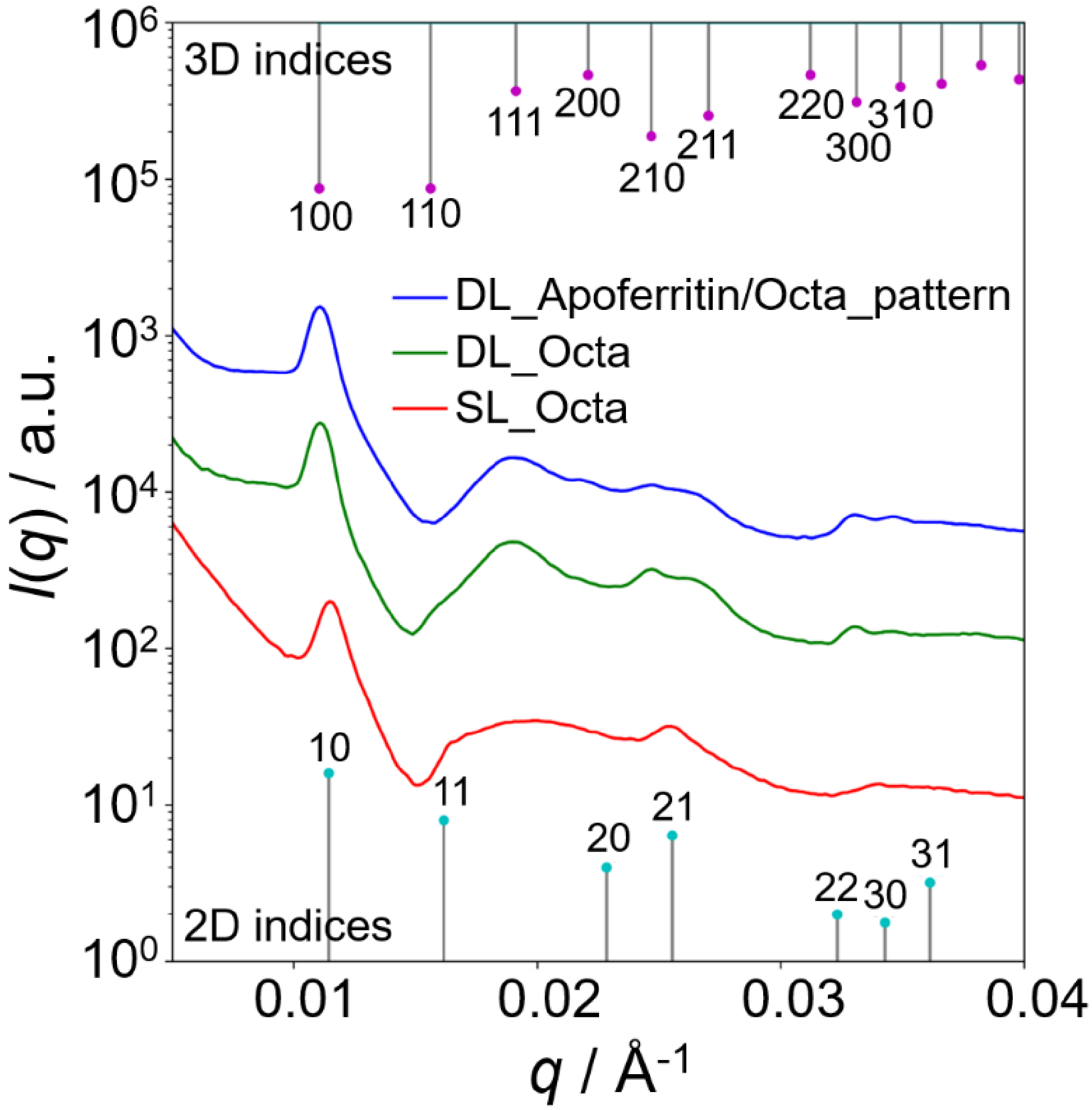

Supplementary Figure 30. SAXS profiles of the 2D double-layered empty Octa and apoferritin/Octa lattices show co-localization of apoferritin and the Octa lattice. Compared to the double-layered ferritin/Octa sample in Fig. 4b, the smaller change in the scattering intensity of apoferritin/Octa relative to an empty Octa lattice was understood by the close electron densities of hollow protein shell and Octa. Compared to a single-layered lattice, formation of a double-layered structure was supported by the emergence of broad peaks at the correspondent 3D indices of a simple cubic lattice, suggesting the presence of an additional layer in the *z* direction.

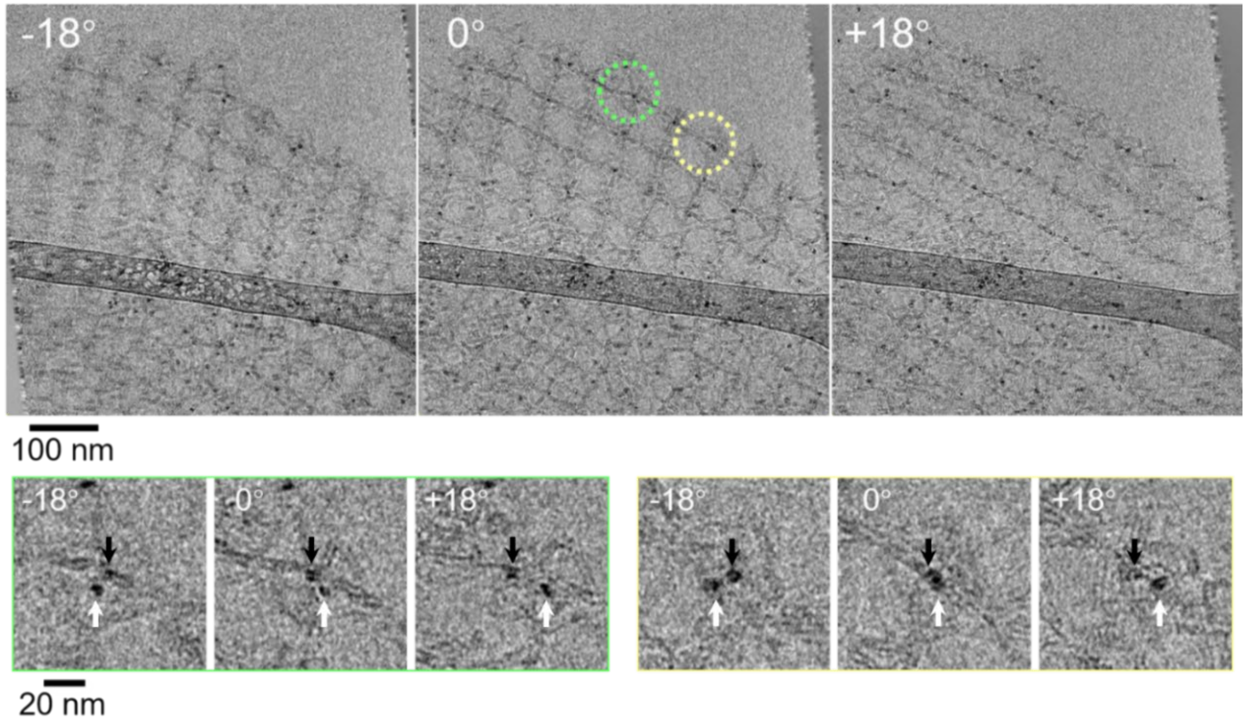

Supplementary Figure 31. Representative tilt images of Cryo-ET of the 2D double-layered ferritin/Octa lattice. The presence of two ferritins at the top and bottom layers was confirmed at tilted angles, which agreed with our design. Magnified images at the circled locations were shown at the bottom and were used in Fig. 4d. The two ferritins at the top and bottom layers were shown at tilted angles and pointed out by the black and white arrows.

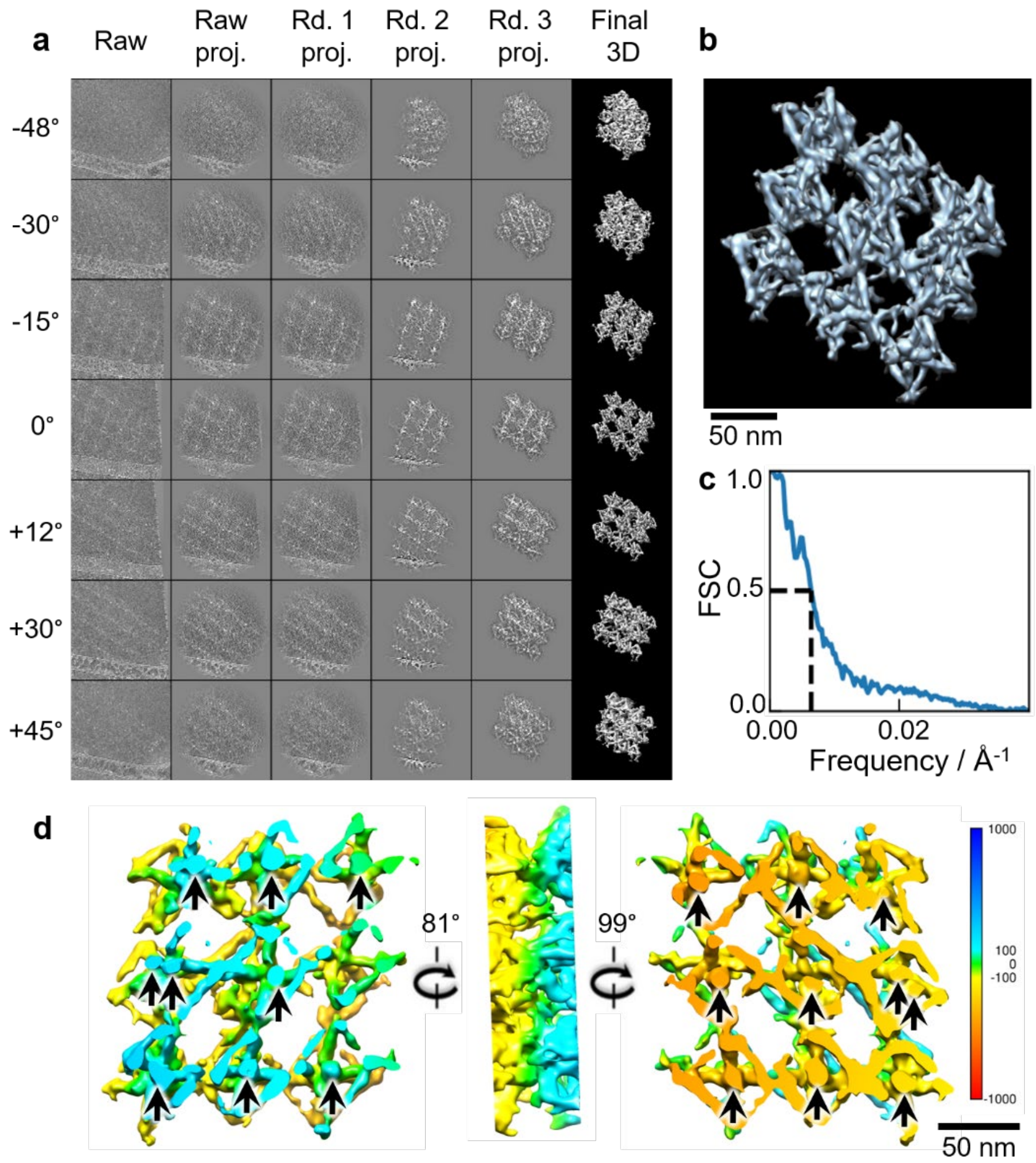

Supplementary Figure 32. IPET 3D reconstruction processes of the double-layered ferritin/Octa lattice. a, Seven representative tilt images of the select area of the ferritin/Octa lattice. The eighteen single ferritin/Octa from the top and bottom layers in the select area are displayed in the first column from the left. Using IPET, the tilt mages are aligned to a common center *via* iterative refinements. The projections of raw, intermediate and final 3D reconstructions at the corresponding tilt angles are displayed in the next five columns. b, The final 3D density map. c, FSC analyses showing the resolution of the final 3D density map, which was estimated as 156 Å. d, A colormap for Fig. 4f showing the overlay of the encapsulated ferritin with the double-layered lattice in one structure. The black arrows indicate the protein positions in each Octa in the lattice. The color bars indicate Z height (unit: Å).

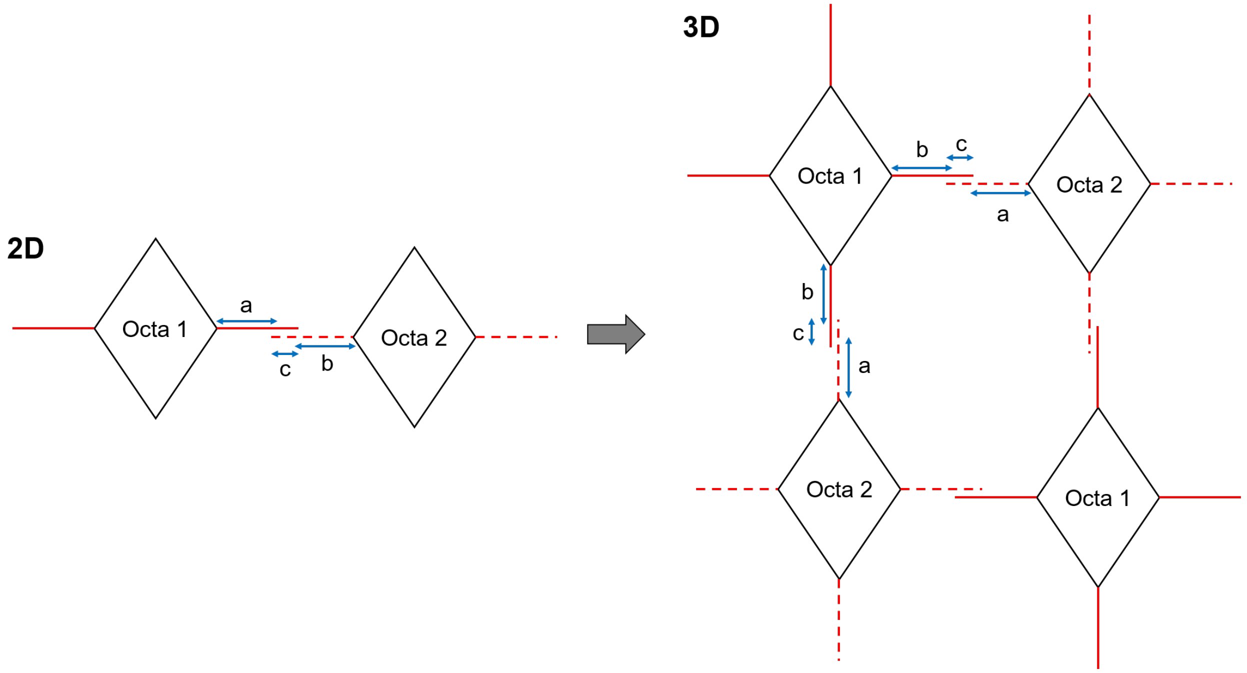

Supplementary Figure 33. Schematic view showing the inter-vertex linking motifs used for the 3D assembly of the Octa lattices and protein/Octa lattices in Fig. 5. The one-colored 3D system is extended from the 2D system in Fig. 3 and Supplementary Fig. 15, in which the red solid lines and dashed lines represented the one set of complementary DNA pairs used in this system. a and b (=22) are the poly T parts from the Octa vertices, and c (=8) is composed of complementary bases that hybridize between strands.

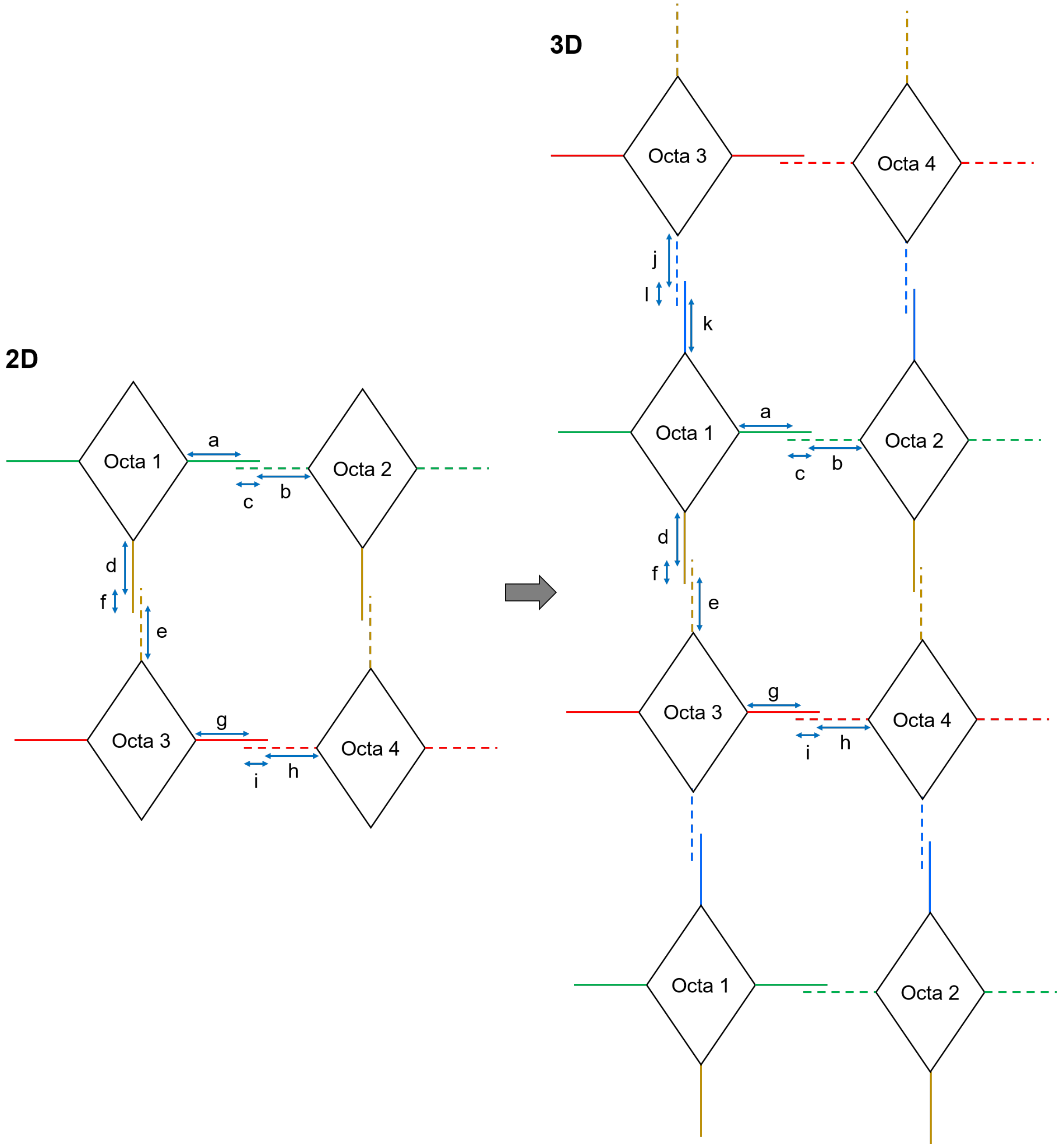

Supplementary Figure 34. Schematic view showing the inter-vertex linking motifs used for the 3D assembly of the Octa lattices and protein/Octa lattices in Fig. 5. The four-colored 3D system is extended from the 2D system in Fig. 4 and Supplementary Fig. 26, in which the solid lines and dashed lines with matching colors extended from the Octa vertices represented the four sets of complementary DNA pairs used in this system. a, b, d, e, g, h, i, k (=22) are the poly T parts from the Octa vertices, and c, e, i and l (=8 or 12) are composed of different complementary bases that hybridize between strands.

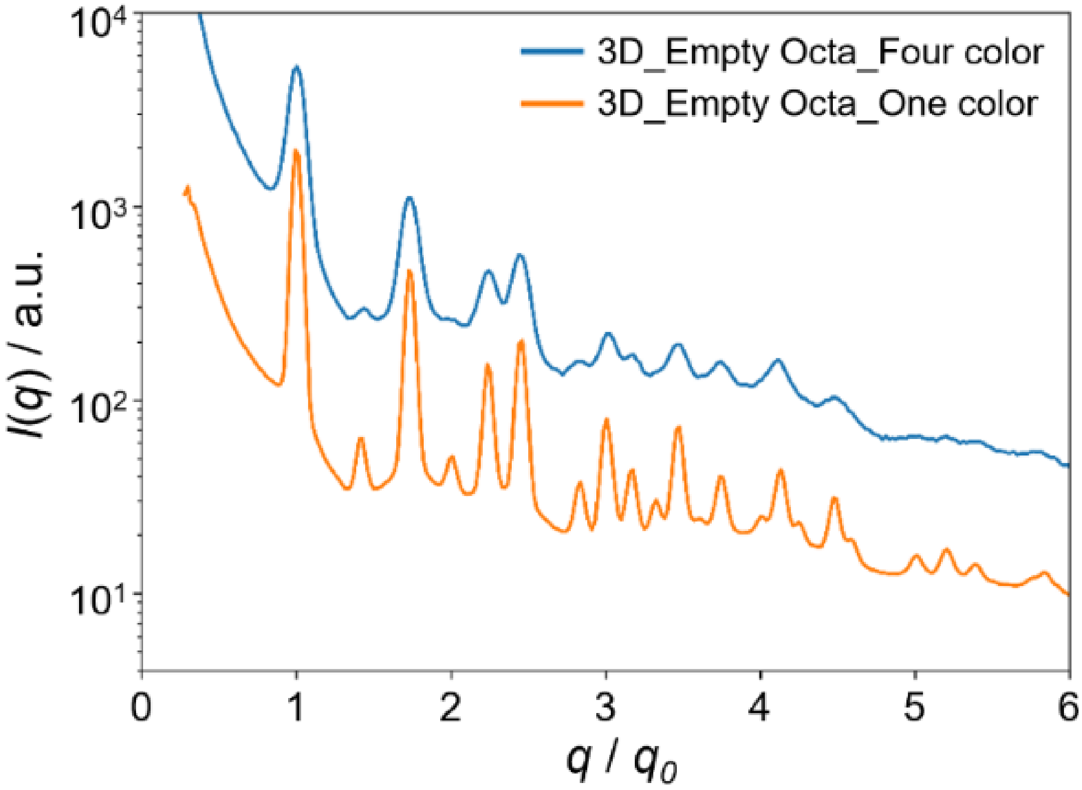

Supplementary Figure 35. Experimental SAXS data of the 3D empty Octa lattice designed with one color (orange curve) and with four colors (blue curve).

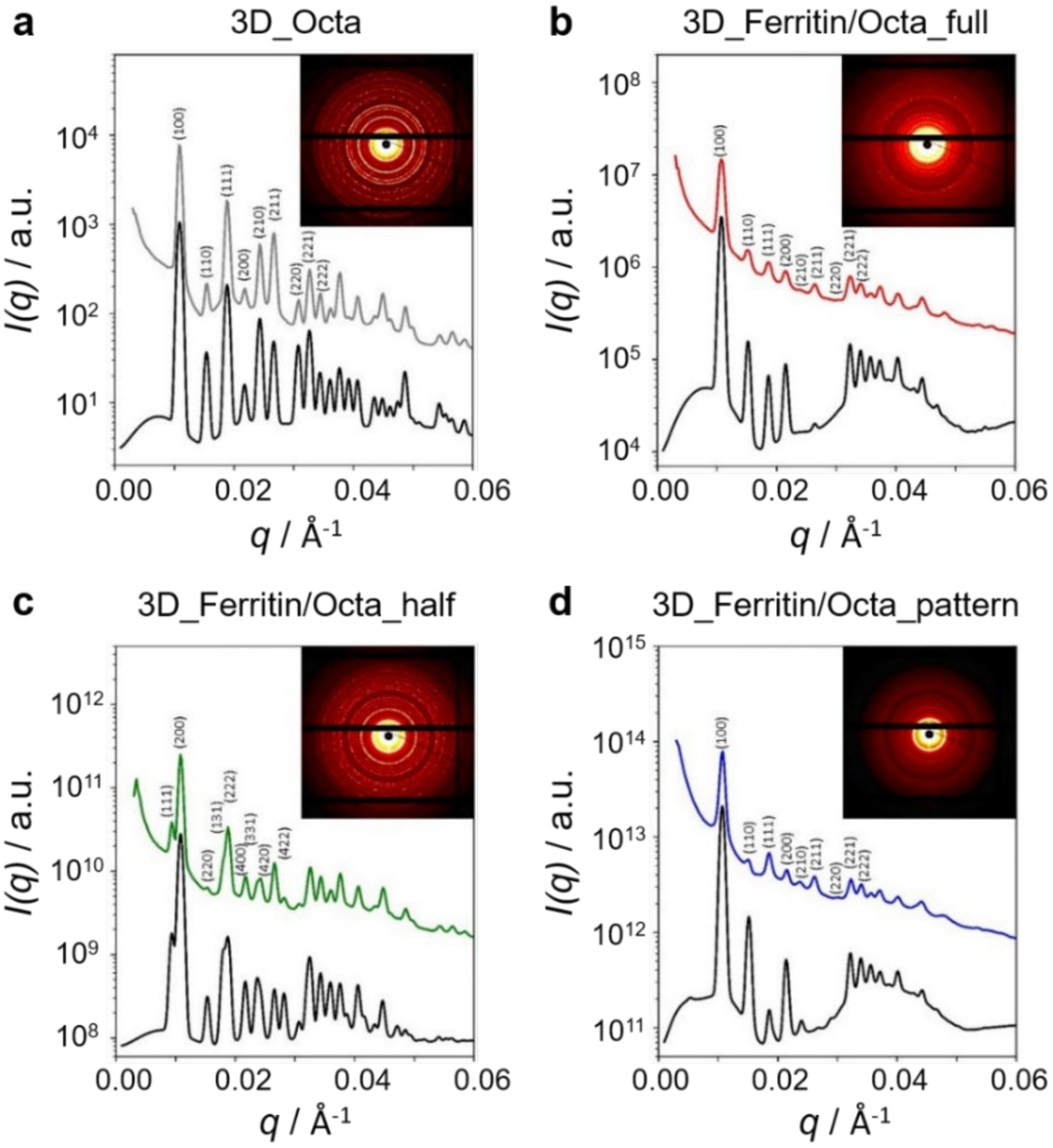

Supplementary Figure 36. Experimental (colored curves) and simulated (black curves) $I(q)$ profiles of the vertex driven Octa frameworks for ferritin assembly shown individually. a, Empty Octa and b, full-filled ferritin/Octa (b) relate to a simple cubit (SC) lattice, where ferritin attenuated the intensity by the stronger scattering at the iron core; c, half-filled ferritin/Octa relates to a face-centered cubic (FCC) lattice; and d, pattern ferritin/Octa relates to a SC lattice with a primitive tetragonal unit cell. Bragg reflections are indicated and the 2D SAXS patterns are shown (upper right inset) for each sample.

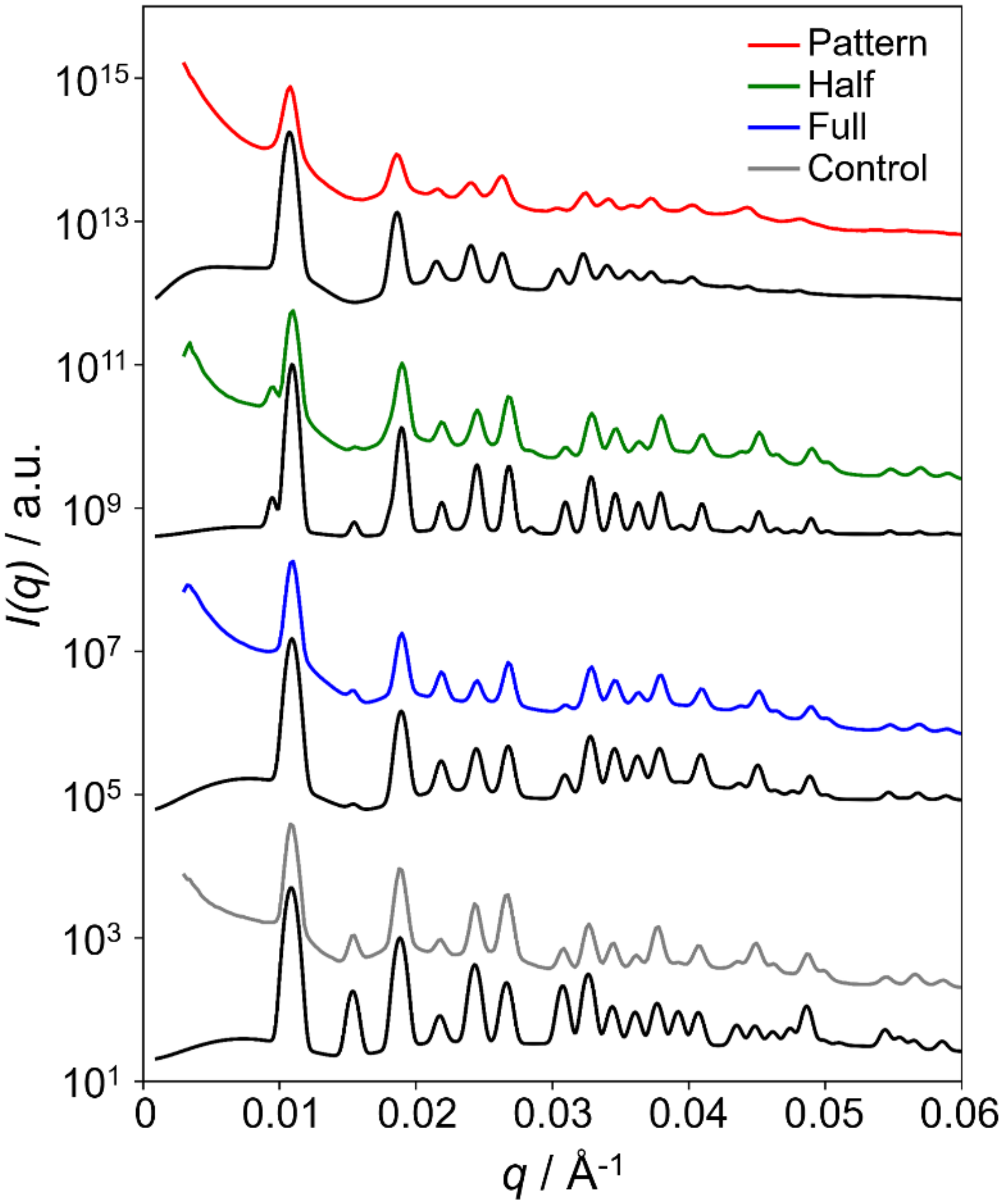

Supplementary Figure 37. SAXS analysis of 3D apoferritin/Octa lattices formed by encoded Octa frameworks. Experimental (colored curves) and simulated (black curves) $I(q)$ profiles of the different types of 3D apoferritin/Octa lattices, in which grey (empty Octa) and blue (full-filled apoferritin/Octa) relate to an SC lattice, green (half-filled apoferritin/Octa) relates to an FCC lattice; and red (pattern apoferritin/Octa) relates to a primitive tetragonal unit cell.

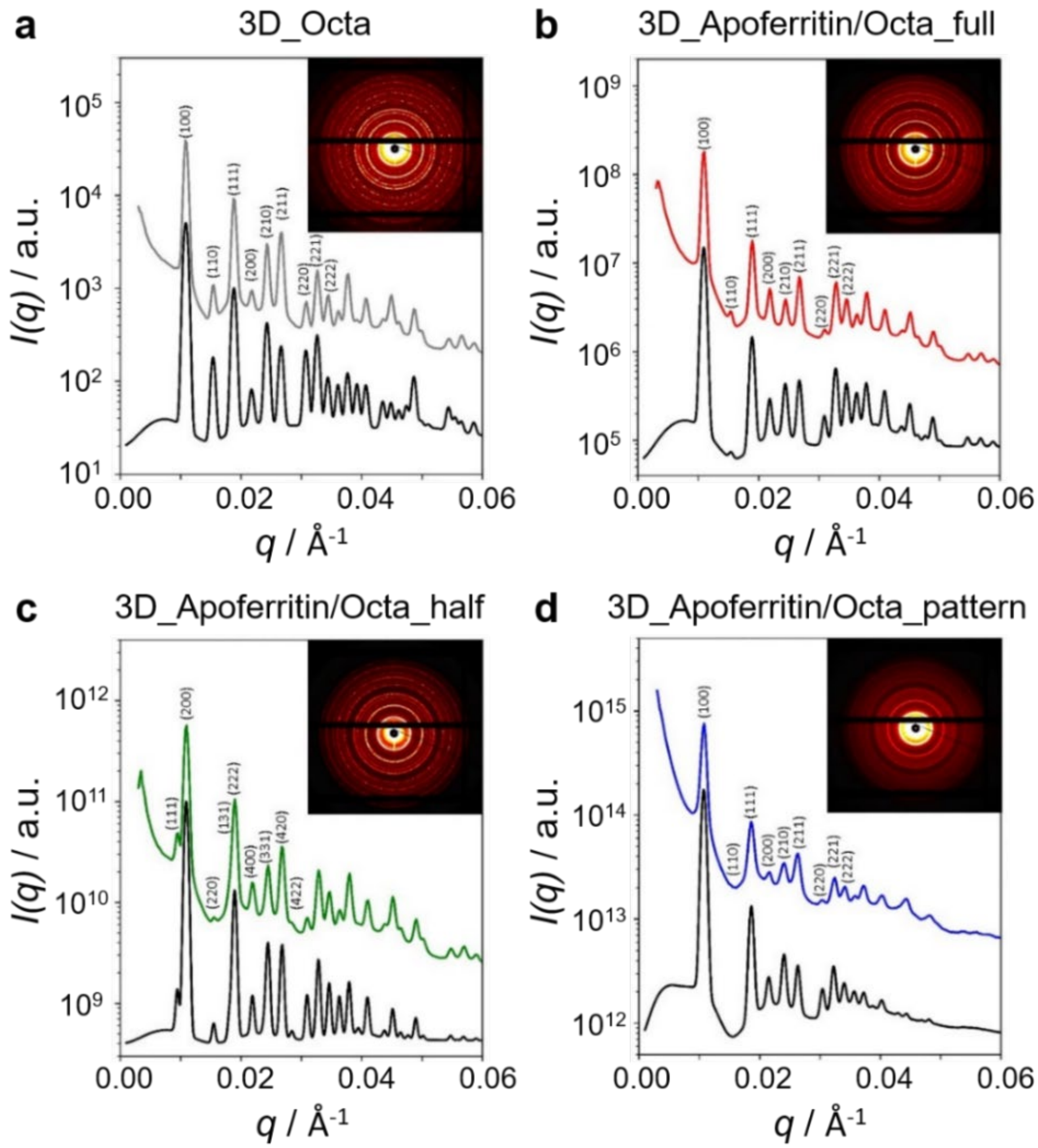

Supplementary Figure 38. Experimental (colored curves) and simulated (black curves) $I(q)$ profiles of the vertex driven Octa frameworks for apoferritin assembly shown individually. a, Empty Octa and b, full-filled apoferritin/Octa relate to a simple cubic (SC) lattice, where ferritin attenuated the intensity by the stronger scattering at the iron core; c, half-filled apoferritin/Octa relates to a face-centered cubic (FCC) lattice; and d, pattern apoferritin/Octa relates to a SC lattice with a primitive tetragonal unit cell. Bragg reflections are indicated and the 2D SAXS patterns are shown (upper right insets) for each sample.

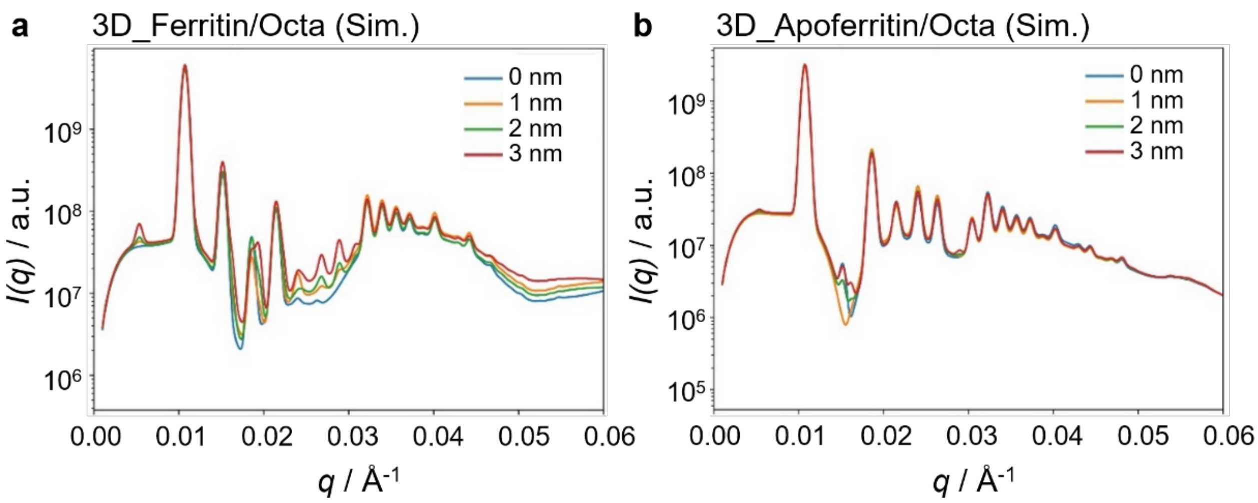

Supplementary Figure 39. Modelling showing the change of SAXS profiles by shifting (by a shown distance for correspondingly colored curves) the protein position from the center of Octa in a 3D lattice: a, shift of ferritin and b, shift of apoferritin.

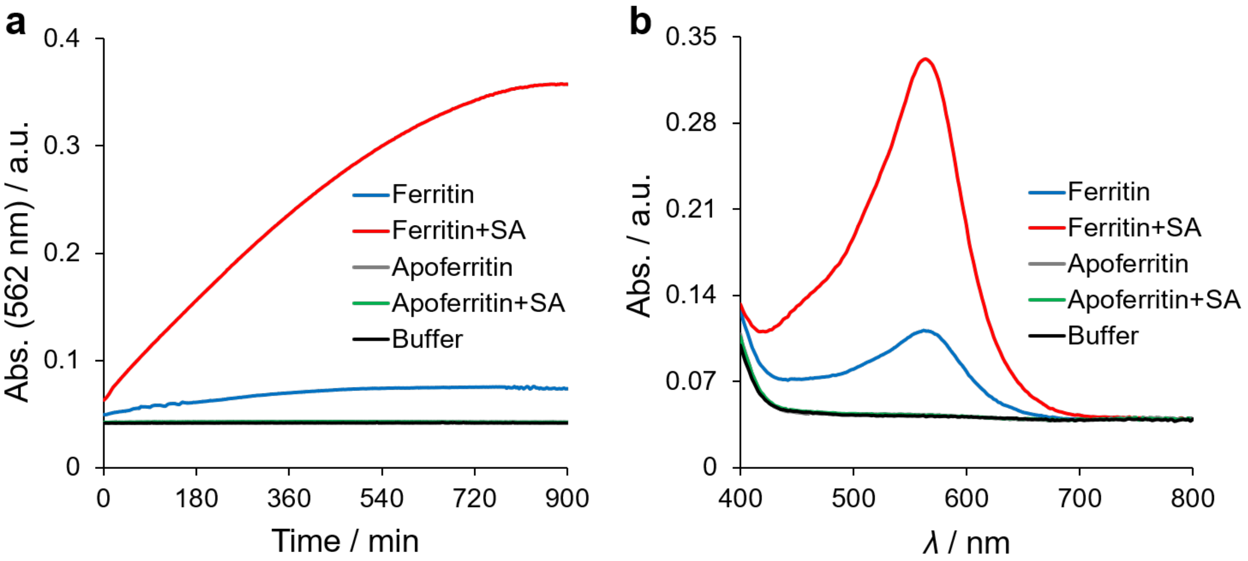

Supplementary Figure 40. a, Kinetic assays and b, absorption spectra of ferritin (50 nM) and apoferritin (50 nM) in the absence or presence of sodium ascorbate (SA, 2.5 mM). SA reduces the Fe^{3+} in ferritin and the released Fe^{2+} in solution forms a complex with ferrozine (2.5 mM), of which the Fe^{2+}-ferrozine complex shows an absorbance at 562 nm. The buffer solution contains 90% PBS buffer (pH 7.4) and 10 v/v% ammonium acetate solution (pH 5.5).

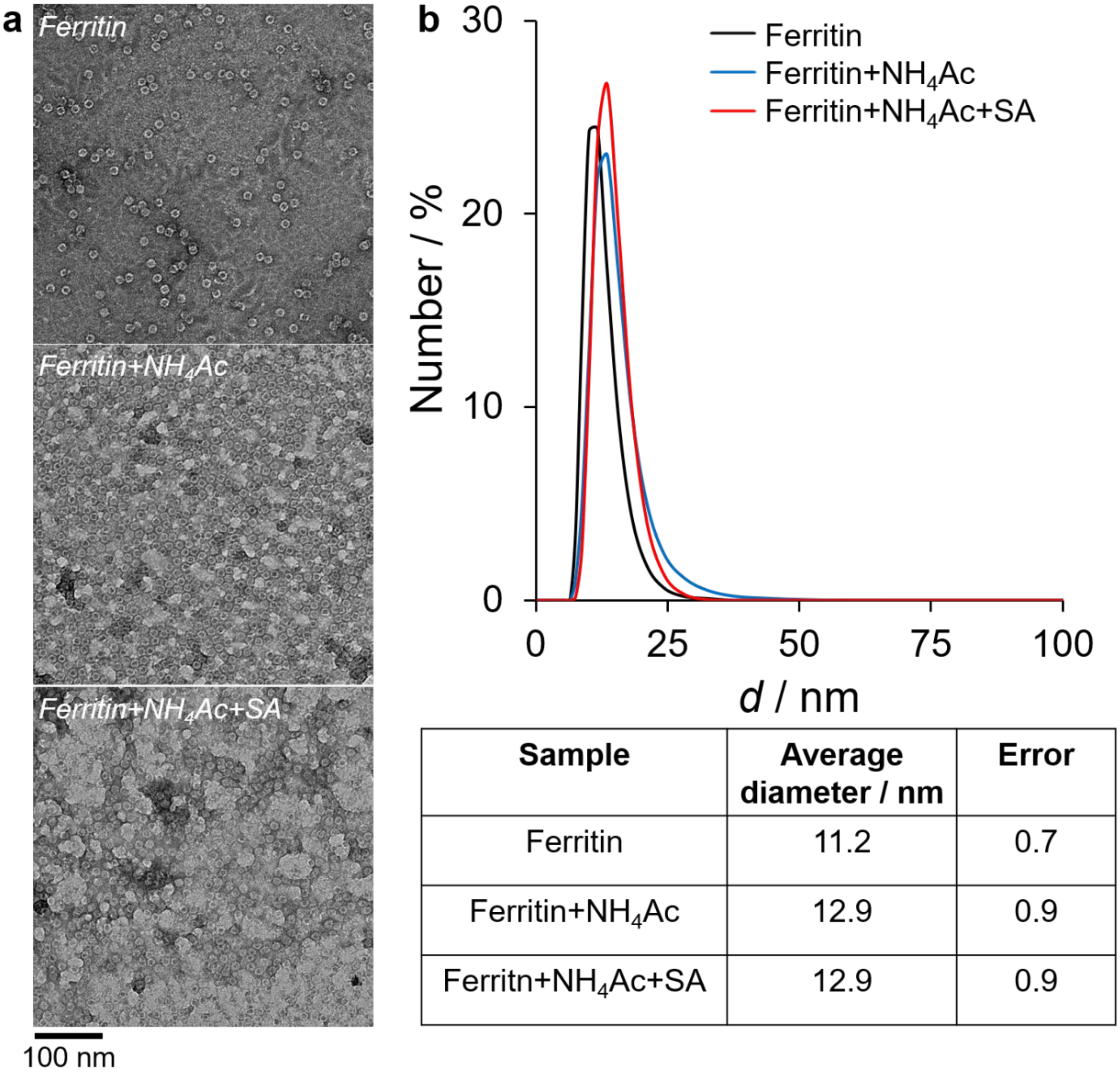

Sample	Average diameter / nm	Error
Ferritin	11.2	0.7
Ferritin+NH_4Ac	12.9	0.9
Ferritn+NH_4Ac+SA	12.9	0.9

Supplementary Figure 41. a, TEM images and b, DLS (n=3) of free ferritin (~2 μM) in PBS buffer (pH 7.4) and in the presence of sodium ascorbate (SA, 20 mM) and 10 v/v% of the ammonium acetate (NH_4Ac, pH 5.5) solution.

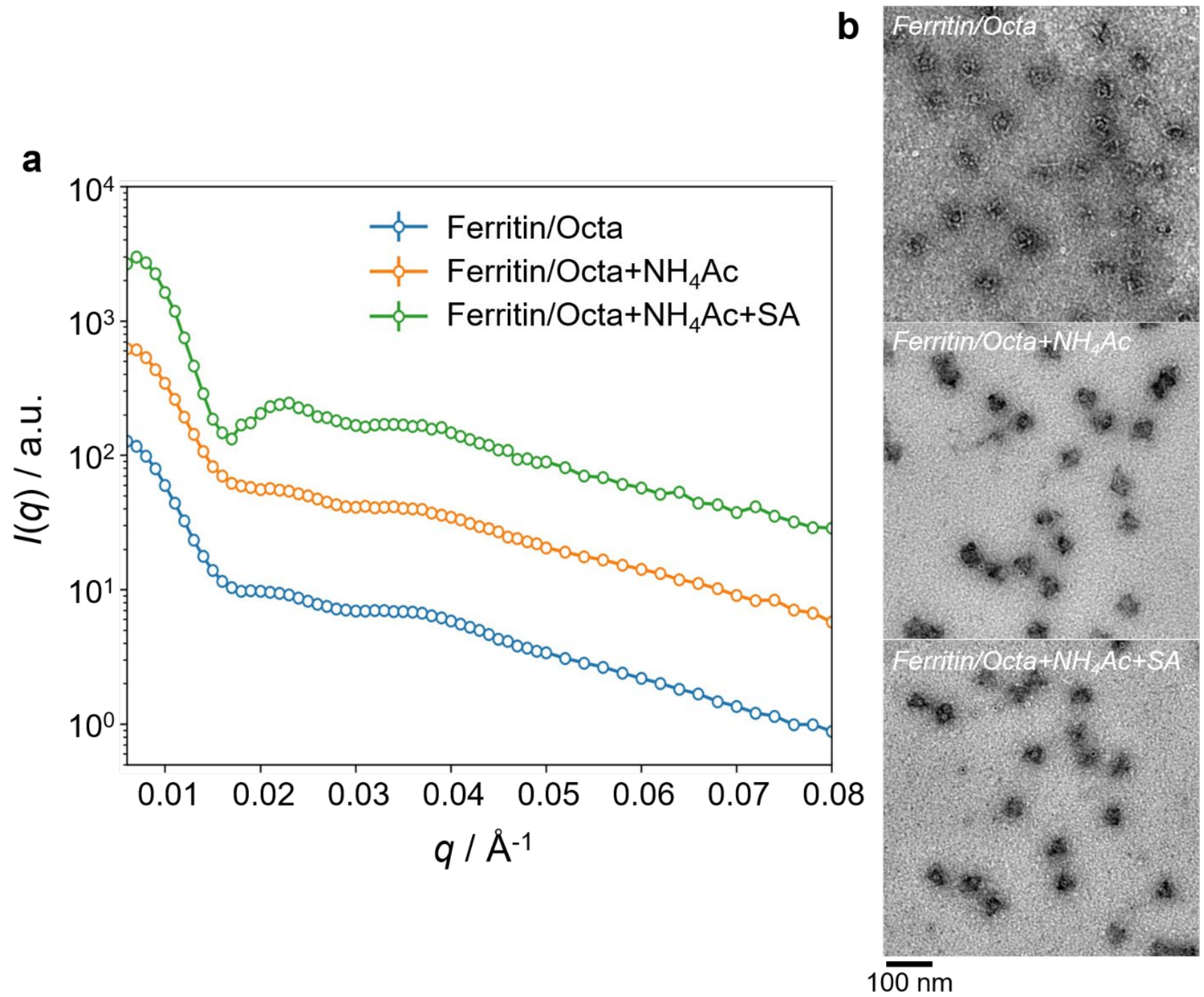

Supplementary Figure 42. a, SAXS analysis and b, TEM images of ferritin/Octa (30 nM) in TAE buffer containing 12.5 mM $MgCl_2$, and in the presence of sodium ascorbate (SA, 20 mM) and 10 v/v% of the ammonium acetate (NH_4Ac, pH 5.5) solution.

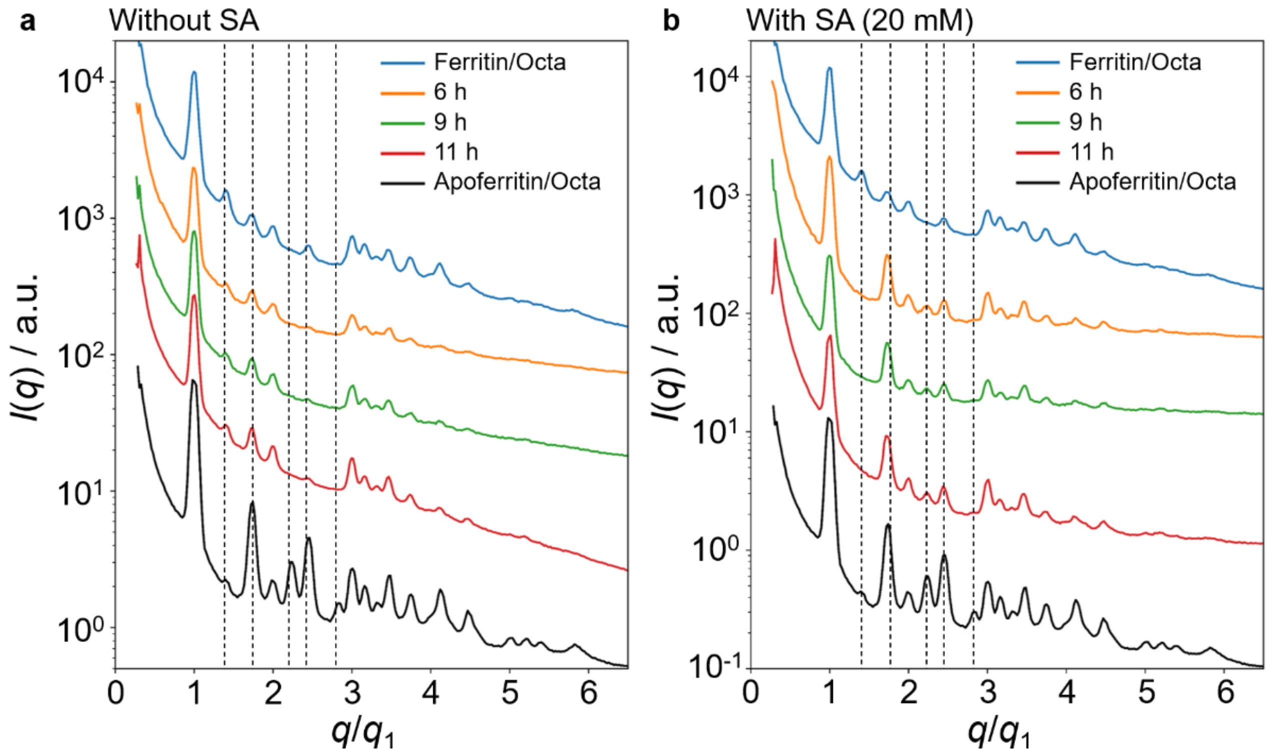

Supplementary Figure 43. Time-dependent SAXS study of the 3D ferritin/Octa lattice in the a, absence or b, presence of sodium ascorbate (SA, 20 mM) and 10 v/v% of the ammonium acetate (NH_4Ac, pH 5.5) solution. The black dashed lines indicate the scattering peaks that are expected to change in intensities during the conversion from a 3D ferritin/Octa lattice to a 3D apoferritin lattice. Peak positions are normalized by the center of the 1st peak.

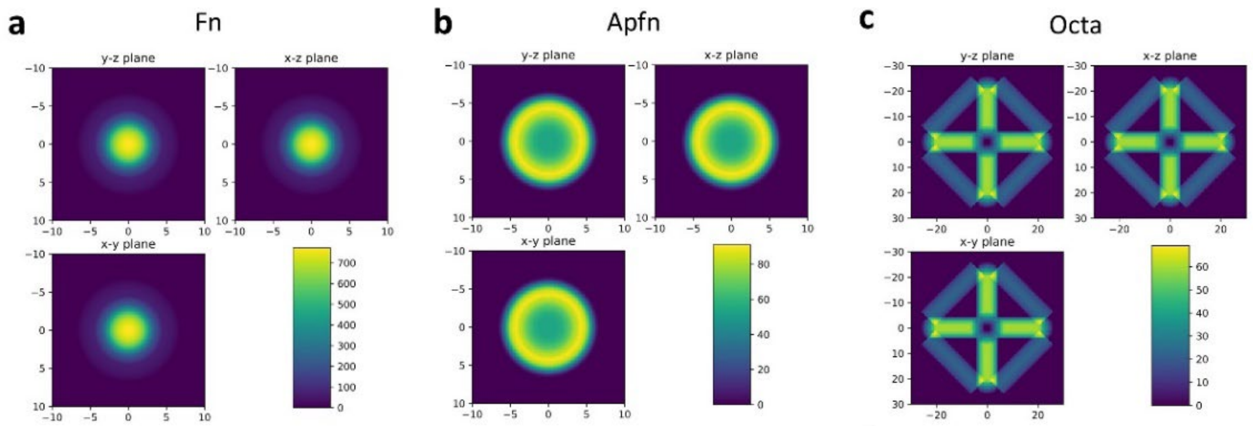

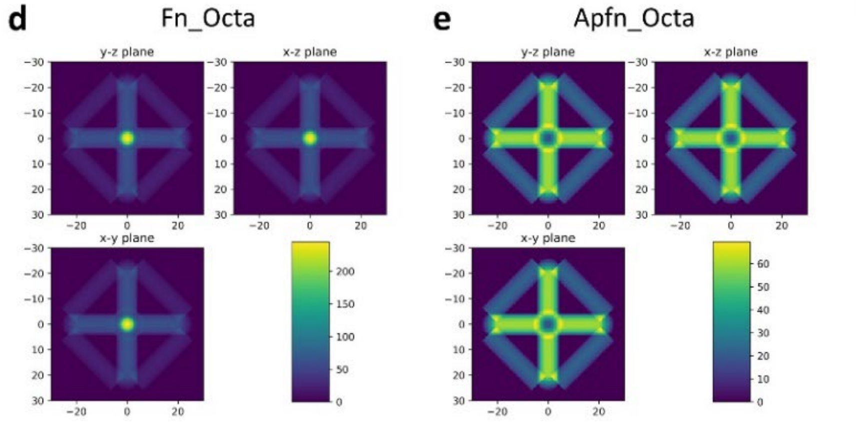

f

Model Parameters		
SLD_water	9.43	x 10^{-6} Å^{-2}
SLD_dsDNA	11	x 10^{-6} Å^{-2}
Cylinder_radius	4.156	nm
Cylinder_height	27.79	nm
Edgelength	38.34	nm
SLD_Fn_shell	10.8	x 10^{-6} Å^{-2}
SLD_Fn_core	31.9	x 10^{-6} Å^{-2}
SLD_Apfn_shell	11.8	x 10^{-6} Å^{-2}
Fn_radius_inner	3.28	nm
Fn_radius_outer	6.74	nm
Apfn_radius_inner	4.03	nm
Apfn_radius_outer	6.05	nm

Supplementary Figure 44. Projections of NanoObjects used in the ScatterSim python library. a, Projections of the CoreShellNanoObject with the model parameters for ferritin (Fn). b, Projections of the CoreShellNanoObject with the model parameters for apoferritin (Apfn). c, Projections of the OctahedronCylindersNanoObject with the model parameters for the octahedral origami (Octa). d, Projections of the CompositeNanoObject with the model parameters for the ferritin isolated inside the modelled Octa (Fn/Octa). e, Projections of the CompositeNanoObject with the model parameters for the apoferritin isolated inside the modelled Octa (Apfn/Octa). f, Parameters used in the SAXS Models. The units for the projections are in nm, and the colormap shows relative electron/scattering length density.

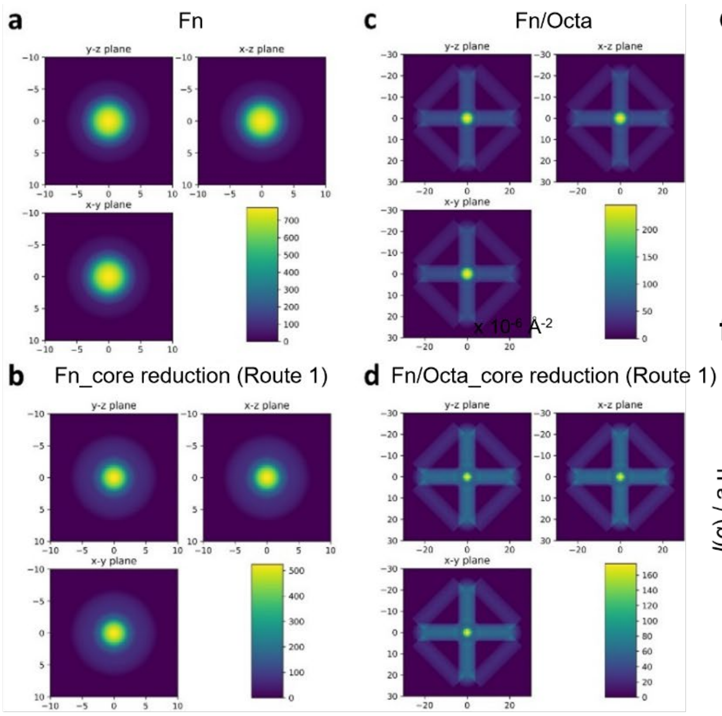

e

Model Parameters		
SLD_Fn_shell	10.8	x 10^{-6} Å^{-2}
SLD_Fn_core	31.9	x 10^{-6} Å^{-2}
Fn_radius_inner	2.296	nm
Fn_radius_outer	6.74	nm
Core reduction	66	%

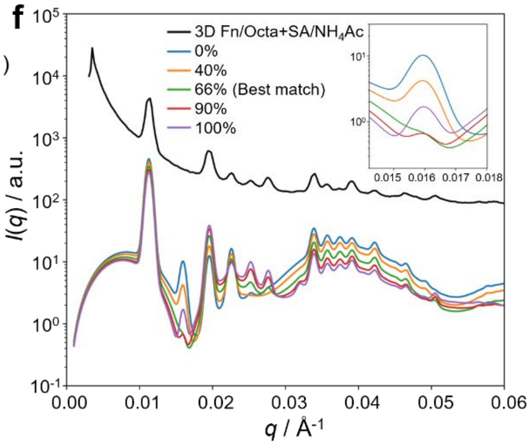

Supplementary Figure 45. Projections of NanoObjects before and after core dissolution based on decrease in size at the surface of the ferritin core (route 1). a-b, Projections of ferritin (Fn) a, before and b, after core reduction, c-d, Projections of ferritin isolated inside the modelled Octa c, before and d, after reduction at the ferritin core. e, Parameters used in the SAXS Models for this dissolution mechanism. The units for the projections are in nm, and the colormap shows relative electron/scattering length density. f, SAXS modelling (colored curves) that correlated the SAXS profiles with core reduction based on the route 1 mechanism was used to analyze the extent of conversion of the ferritin array, of which the experimental result (black curve) corresponded the best to an average of 66% of reduced cores. Ferritin reduction was performed using sodium ascorbate (SA, 20 mM) solution containing ammonium acetate (NH_4Ac, pH 5.5). Inset: zoomed-in region of SAXS models for the 2nd peak, or the (110) Bragg plane.

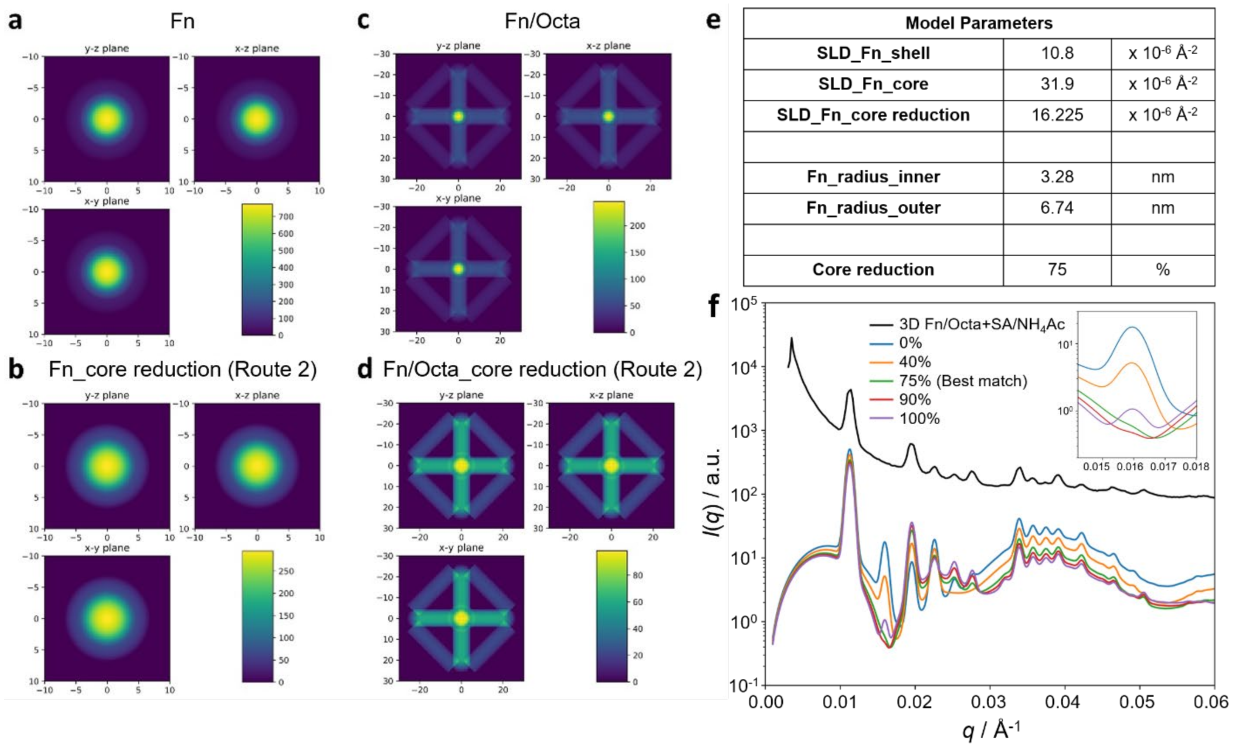

Supplementary Figure 46. Projections of NanoObjects before and after core dissolution based on reduction of the overall electron density of the ferritin core (route 2). a-b, Projections of ferritin (Fn) a, before and b, after core reduction. c-d, Projections of ferritin isolated inside the modelled Octa c, before and d, after reduction of the ferritin core. e, Parameters used in the SAXS models for this dissolution mechanism. The units for the projections are in nm, and the colormap shows relative electron/scattering length density. f, SAXS modelling (colored curves) that correlated the SAXS profiles with core reduction based on the route 2 mechanism was used to analyze the extent of conversion of the ferritin array, of which the experimental result (black curve) corresponded the best to an average of 75% of reduced cores. Ferritin reduction was performed using sodium ascorbate (SA, 20 mM) solution containing ammonium acetate (NH_4Ac, pH 5.5). Inset: zoomed-in region of SAXS models for the 2nd peak, or the (110) Bragg plane.

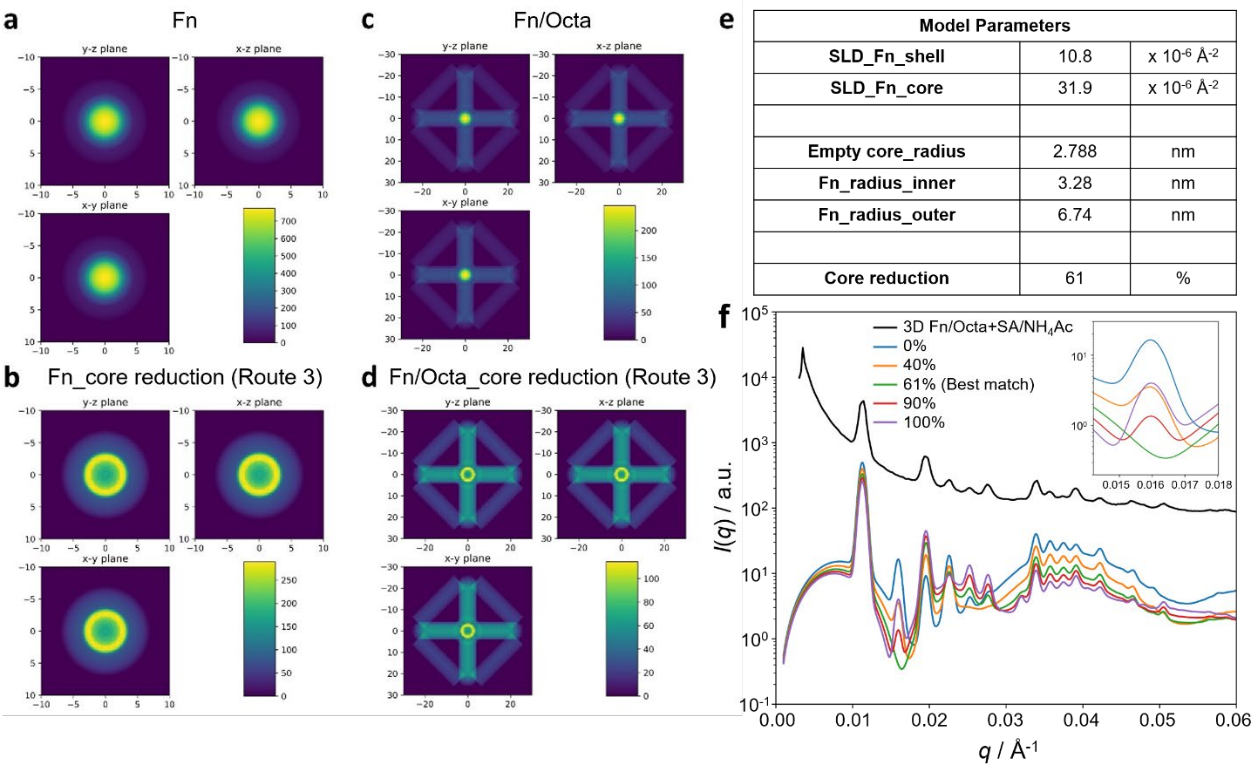

Model Parameters		
SLD_Fn_shell	10.8	x 10^{-6} Å^{-2}
SLD_Fn_core	31.9	x 10^{-6} Å^{-2}
Empty core_radius	2.788	nm
Fn_radius_inner	3.28	nm
Fn_radius_outer	6.74	nm
Core reduction	61	%

Supplementary Figure 47. Projections of NanoObjects before and after core dissolution based on decrease in size from the center of the ferritin core (route 3). a-b, Projections of ferritin (Fn) a, before and b, after core reduction. c-d, Projections of ferritin isolated inside the modelled Octa c, before and d, after reduction of the ferritin core. e, Parameters used in the SAXS models for this dissolution mechanism. The units for the projections are in nm, and the colormap shows relative electron/scattering length density. f, SAXS modelling (colored curves) that correlated the SAXS profiles with core reduction based on the route 3 mechanism was used to analyze the extent of conversion of the ferritin array, of which the experimental result (black curve) corresponded the best to an average of 61% of reduced cores. Ferritin reduction was performed using sodium ascorbate (SA, 20 mM) solution containing ammonium acetate (NH_4Ac, pH 5.5). Inset: zoomed-in region of SAXS models for the 2nd peak, or the (110) Bragg plane.

Supplementary Methods

Materials

Single-stranded DNA (ssDNA) sequences were purchased from Integrated DNA Technologies and the M13mp18 ssDNA scaffold was purchased from Bayou Biolabs. Ferritin and apoferritin from horse spleen, phosphate buffered saline (PBS), magnesium chloride ($MgCl_2$), azido-dPEG$_8$-NHS ester, agarose (medium EEO), ferrozine ($C_{20}H_{13}N_4NaO_6S_2$) ammonium hydroxide, acetic acid, and phosphate buffered saline were purchased from Sigma Aldrich. (+)-Sodium L-ascorbate was purchased from Fluka. The 10X tris-acetate-ethylenediaminetetraacetic acid (TAE) and 10X tris-borate- ethylenediaminetetraacetic acid (TBE) buffers, 10X BlueJuice gel loading buffer, SYBR Gold (10,000x) dye were purchased from ThermoFisher Scientific. Carbon grid (200 mesh copper) and uranyl acetate solution were purchased from Electron Microscopy Sciences. Milli-Q (18 MΩ · cm) was used for all the experiments.

Agarose gel electrophoresis (AGE)

For AGE of Octa structures, agarose (1.0 wt%) was prepared in TBE (1X) buffer containing 12.5 mM $MgCl_2$ and 1X SYBR Gold dye. Octa and protein/Octa (10 nM, 20 μL) were mixed with 1X BlueJuice gel loading buffer prior to loading into the gel. Electrophoresis was performed at 60 V on ice to prevent heating damage. Similarly, AGE of ssDNA modified proteins was performed by mixing protein samples (1 μM, 20 μL) with loading buffer. After loading, electrophoresis was performed at 70 V on ice.

Dynamic light scattering (DLS)

Octa (5 nM) was dispersed in TAE buffer containing 12.5 mM $MgCl_2$ and proteins (0.1–2 μM) were dispersed in PBS buffer. The samples were measured 3 times with Zetasizer Nano Z (Malvern Analytical) with an equilibrium time of 120 s.

Small-angle X-ray scattering (SAXS) experiment and analysis

1) Data acquisition of proteins, single Octa and single protein/Octa:

Solution scattering data was collected at the Life Sciences X-ray Scattering beamline (LiX, 16-ID) at the National Synchrotron Light Source II (NSLS-II), Brookhaven National Laboratory (BNL). LiX utilizes an undulator source and a Si(111) monochromator. KB mirrors focus the

beam on a secondary source and X-ray energy was 12 keV with a beam size of ~400 um. An in-house solution scattering box houses a movable 3 channel flow cell such that proteins in solution flow through the beam during collection. Data was collected on 3 Pilatus detectors (SAXS: Pilatus 1M, 2 offset WAXS detectors: Pilatus 300K).[1] The data was merged, averaged, subtracted and packed into HDF5 format using the in-house py4xs software[2], with data visualization in jupyter notebook. For each sample exposed to the X-ray beam, five frames, with an exposure time of 1 s was collected and processed using py4xs.

2) SAXS data acquisition of 2D and 3D Octa and protein/Octa lattices:

The scattering data of Octa and protein/Octa lattices were collected at the Complex Materials Scattering (CMS, 11-BM) beamline at the NSLS-II at BNL. Measurements were carried out in transmission mode with the X-ray energy E = 13.5 keV (λ = 0.9184 Å) using a 2D detector, Pilatus 1M, with a sample-to-detector distance of 5.05 m. The 2D scattering images were converted into 1D scattering intensity profiles through azimuthal integration. The intensity $I(q)$ is expressed as a function of the scattering vector magnitude q, where $q = \frac{4\pi \sin\theta}{\lambda}$ with 2θ being the full scattering angle.

3) SAXS modelling:

The SAXS modeling was primary performed using the ScatterSim software package[3,4], a python library for simulating 1D curves for the clusters and superlattices built from arbitrary anisotropic nanoscale objects.[3,4]

a. Modeling of single-particle proteins

The ferritin and apoferritin were modelled with a core-shell structure.

$$F(q, r_{core}, r_{total}) = 3\left[V_{core}(\rho_{core} - \rho_{shell})\frac{\sin(qr_{core}) - qr_{core}\cos(qr_{core})}{(qr_{core})^3} + V_{total}(\rho_{shell} - \rho_{solvent})\frac{\sin(qr_{total}) - qr_{total}\cos(qr_{total})}{(qr_{total})^3}\right] \quad (1)$$

where ρ_{core}, ρ_{shell} and $\rho_{solvent}$ are electron density of the core, shell and solvent, respectively; r_{core} and V_{core} are the radius and the volume of the core; r_{total} and V_{total} are the radius and the volume of the whole core-shell structures.

The scattering form factor $P(q)$ is expressed as,

$$P(q) = \frac{|F(q, r_{core}, r_{total})|^2}{V_{total}} \quad (2)$$

For the polydisperse core-shell structure,

$$P(q) = \int \frac{|F(q, r_{core}, r_{total})|^2}{V_{total}(r_{core}, r_{total})} D(r_{core}) D(r_{total}) dr_{core} dr_{total} \quad (3)$$

where $D(r)$ is the size distribution function, and the Gaussian distribution is used in this model. We note that $\int D(r_{core}) D(r_{total}) dr_{core} dr_{total} = 1$.

The fitting results of free ferritin (Fn) and apoferritin (Apfn) in the aqueous solutions are shown in Fig. 2. The apoferritin was modeled as a hollow protein with a spherical shell. The electron density (ED) of the shell was estimated be 0.42 e/Å^3, corresponding to a scattering length density (SLD) of $SLD_{shell\text{-}Apfn} = 11.8 \times 10^{-6}$ Å^{-2}. The SLD of the aqueous solution inside and outside of the protein shell was $SLD_{water} = 9.43 \times 10^{-6}$ Å^{-2}. Based on the fit, the radii of the apoferritin core and shell were $r_{core\text{-}Apfn} = 4.03 \pm 0.81$ nm and $r_{total\text{-}Apfn} = 6.05 \pm 0.43$ nm, respectively.

The ferritin was modeled as an iron-capsulated apoferritin, *i.e.*, a spherical core-shell structure with a protein shell and an iron-concentrated core. The ferritin shell was slightly expanded compared to apoferritin, where the SLD of the ferritin shell was $SLD_{shell\text{-}Fn} = 10.8 \times 10^{-6}$ Å^{-2}. Based on the fitting result shown in Fig. 2, we obtained $r_{core\text{-}Fn} = 3.28 \pm 0.61$ nm, $r_{total\text{-}Fn} = 6.74 \pm 0.26$ nm and $SLD_{core\text{-}Fn} = 31.9 \times 10^{-6}$ Å^{-2}.

b. Modeling of single-particle Octa

The Octa model was built using "OctahedronCylindersNanoObject" in the ScatterSim package. The octahedral DNA frame was made up of 12 edges, and each edge of the Octa was a six-helix bundle (6HB) and was modelled as a cylinder with a radius of $r_{cylinder}$ and a length of $h_{cylinder}$. The overall length of the edge of the octahedron is L_{octa}. The SLD of the 6HB was approximately 11×10^{-6} Å^{-2}. Based on the best fit of the SAXS profile, we obtained $r_{cylinder} = 4.16$ nm, $h_{cylinder} = 27.8$ nm, and $L_{octa} = 38.3$ nm, which agreed with the Octa design.

The protein encapsulated Octa (protein/Octa) was considered as a non-overlapping "composite" object of the Octa frame and a protein inside. Such composite structure was modelled using the "CompositeNanoObject" in ScatterSim, which took into account the interference between the sub-components. We note that in our SAXS modelling we have refrained from introducing fixed distortion of octahedron shapes and polydispersity of its shape. While this effect might be present to some degree, as suggested by the cryo-EM single particle reconstruction, it also revealed that distortion was relatively small. Adding such effects in SAXS scattering would introduce several additional petameters and will increase a complexity of modeling significantly. Such studies will require a dedicated investigation, which will be addressed in the future work. We assumed that protein encapsulation in the Octa did not change the overall geometry of both

the protein and the Octa, as confirmed by our electrophoresis, EM and DLS results (Fig. 2, Supplementary Figs. 10–12).

Thus, the SAXS modeling parameters, such as geometry and SLD of the sub-components, were obtained from the fitting results of the free-dispersed individual sub-components. As shown in Supplementary Fig. 13, apoferritin/Octa showed a similar scattering profile to the Octa only; however, the 1st local minimum shifted to the high q while the 2nd shifted to the low q after the apoferritin encapsulation. These features were consistent with simulated results. In terms of ferritin/Octa, the scattering intensities of first two local maxima (at 0.022 Å^{-1} and 0.038 Å^{-1}) changed dramatically as the electron density of the ferritin core was much higher than the protein shell and the Octa frame. Particularly, the 1st maximum was suppressed, and the 2nd maximum was enhanced, which were confirmed by the modeling.

The relative position of the protein to the Octa structures were tuned in our designs (see designs of Off1 and Off2 in Supplementary Fig. 7). In the SAXS model, the position of protein inside Octa could be controlled in the "composite" model. By shifting the position of the protein from the center of the Octa towards one of the vertices by a specific distance d_{shift}, center-shifted protein/Octa model were built. Specifically, we assumed that $d_{\text{shift-Off1}}$ = 3 nm and $d_{\text{shift-Off2}}$ = 5 nm. The modeling results showed that the increase of the d_{shift} led to the increase of the 1st local maxima of the scattering profiles for ferritin/Octa while apoferritin/Octa did not show any clear change. All these trends and features shared similarity with the experimental observations.

c. Modeling of protein and DNA origami arrays

To model the 2D and 3D protein arrays, the protein/Octa composite was used as a simple object to build unit cells of the lattice using ScatterSim. More details of the 3D SAXS analysis can be found in our recent work[5]. The scattering intensity profiles $I(q)$ for the periodic lattices were simulated by:

$$I(q) = cZ_0(q)G(q) + P(q)\big(1 - \beta(q)G(q)\big) \qquad (4)$$

where c is an overall scaling factor, $P(q)$ is the form factor intensity of the composite object comprised of all objects in the lattice (assuming their relative positions and orientations were preserved), and

$$Z_0(q) = \frac{1}{q^2}\sum_{\{hkl\}}^{m_{hkl}} \left|\sum_{j=1}^{N}\langle F_j(\boldsymbol{q}_{hkl})\rangle_\epsilon exp\big[2\pi i\big(x_j h + y_j k + z_j l\big)\big]\right|^2 L(q - q_{hkl}) \qquad (5)$$

is known as the lattice factor, where $L(q - q_{hkl})$ is a peak shape function. The structure and symmetry of the lattice was taken into account by properly sampling over the correspondent Miller indices q_{hkl}. *G(q)* is the Debye-Waller factor arise from thermal vibrations in the lattice and is defined as:

$$G(q) = e^{-q^2 \sigma_{D,rms}^2} \qquad (6)$$

where $\sigma_{D,rms}$ is the rms displacement of the elements in the lattice. For a simple cubic lattice of unit cell size a, the equation can be re-written as a fractional displacement of the lattice length as $\sigma_{DW} = \frac{a}{\sigma_{D,rms}}$. Finally, $\beta(q)$ is defined as:

$$\beta(q) = \frac{|\langle F(\boldsymbol{q})\rangle_\epsilon|^2}{\langle |F(\boldsymbol{q})|^2\rangle_\epsilon} \qquad (7)$$

and arises from any polydispersity in parameters ϵ. The effect of *G(q)* under increasing thermal vibrations leads to a reduced ordered scattering from $Z_0(q)$ and an increased the diffuse scattering from unordered elements. In our case, the effect of $\beta(q)$ was only considered for polydisperse protein due to the more complex trend that depended on the length scales of the parameters ϵ.

The procedure for SAXS modeling of the lattice is briefly described below. First, for the lattice components, we used the same morphological and scattering parameters as those obtained from the single-particle form factor analysis, including size, shape and SLD. Based on these parameters, we built the lattice components with *P*(*q*) and $\beta(q)$ being calculated. Next, a lattice geometry (*i.e.*, 2D square, SC, FCC, *etc.*) was introduced and the lattice constants were extracted by fitting the primary peak of the experimental intensity profile *I*(*q*) using the built lattice model. Finally, we manually adjusted the Debye-Waller factor, peak shape parameters, scaling factors and the background to match the experimental measurements with the simulated results, based on the empirically the influences of each parameter on lattice properties and the as-simulated intensity profiles, as studied previously[3]. We did not apply direct non-linear least squares fitting of the intensity profiles because (i) such fitting is extremely time-consuming due to the complex architectures with different types of components in our lattice models and (ii) a fit convergence is limited due to the contribution of diffuse scattering.

Cryogenic electron microscopy (cryo-EM) image processing and 3D reconstruction:

All data processing and reconstruction were done in RELION3[6,7], including motion correction, CTF estimation, particle picking. 2D classification, 3D initial model, 3D classification and

refinement. Dark gain corrected Movies were imported into RELION3, motion correction was done with RELION's own implementation and CTF estimation was done with CTFFIND4[8]. Particle picking was first done by LoG-based auto-picking (reference-free) in RELION3, and all particle images were then manually inspected in order to remove "bad" particles (partially assembled, of low contrast, or contacting other particles and/or edges of carbon supporting film) and to add missed good particles. The CTF corrected data were then subject to several rounds of unsupervised 2D image classification in an iterative manner in order to get rid of images assigned to bad classes. Particle images assigned to the same class were mutually aligned and averaged to generate a set of high-contrast class averages (good 2D classes) that represented the single apoferritin/Octa structure. A low-resolution reference-free 3D initial model was generated by using particle images assigned to representative 2D classes and imposing no symmetry (C1 symmetry). The starting model was low-pass filtered to 80Å and used for 3D refinement against the CTF corrected particle image dataset without imposing any symmetry (C1 symmetry) throughout the 3D refinement in RELION3. The resolution of the final 3D map was estimated by the so-called "Gold standard" Fourier shell correlation method at the threshold of 0.143.[9] All image processing and 3D reconstruction were done on a 4-GPU Exxact Linux workstation or a 2-GPU Dell Linux workstation. 3D density map was displayed and manipulated in the UCSF Chimera package[10].

Cryo-electron tomography (cryo-ET) image processing and 3D reconstruction:

Anisotropic image motion of each frame of image stack in super-resolution mode (0.73 Å/pixel) was corrected by MotionCor2[11]. All tilt series were binned by two times (1.46 Å/pixel) and aligned by IMOD[12]. Defocus values were measured by Gctf[13] and the contrast transfer function (CTF) were corrected by TomoCTF[14]. 3D density maps of the whole micrograph were aligned and reconstructed by IMOD, after tilt series were binned eight times (11.68 Å/pixel). For high-resolution 3D structure, a tilt series of a focused area with ~336 × 336 nm^2, *i.e.* 288 pixel × 11.68 Å/pixel, was windowed and extracted from the whole micrographic tilt series following the IPET reconstruction protocol[15]. In this process, an *ab initio* 3D map back-projected from the raw tilt series was used as an initial model for iterative refinement. During the iteration, a set of Gaussian low-pass filter, and circular and particle-shaped soft-boundary masks were automatically generated and sequentially applied to the tilt images to increase their SNRs during alignment.

To reduce artifact of missing wedge, the 3D map back-projected from the aligned tilt series was submitted for a post process for missing wedge correction[16]. The resolution was estimated by calculating the Fourier shell correlation (FSC) curve between two-halves of the reconstructed 3D maps that were generated from odd and even aligned tilt series[17]. The frequency at which the FSC curve first fell to 0.5 was used to estimate the resolution of the IPET 3D density map. All 3D maps in the figures were low-pass filtered to 80 Å using ENAN software[17] and displayed in the UCSF Chimera package[10].

Ferrozine-protein assays:

Ferrozine stock solution (10 mM) was prepared by dissolving ferrozine in deionized water. For pH adjustment of the ferrozine solution, an ammonium acetate buffer stock solution was prepared by mixing 19.5 mL of ammonium hydroxide (28% v/v) solution and 15 mL of acetic acid (80%) solution, adjusting the pH to 5.5 with HCl (1 N) solution and making up to 50 mL with deionized water. The ascorbate stock solution (50 mM) was prepared by dissolving sodium ascorbate in deionized water. The ferrozine working solution was prepared by mixing the ferrozine stock solution (5 mL) with the ammonium acetate buffer (3.34 mL) and 1.66 mL of deionized water.

To prepare the ferrozine assays for free ferritin and apoferritin, ascorbate (1 mM and 2.5 mM) and the ferrozine working solution (1 mM) were added to the proteins (50 nM) in PBS. The absorbance kinetics and spectra were measured in a 96 well plate (Corning) using the Spark microplate reader (Tecan). The kinetic assay for tracking the ferrozine-Fe^{2+} complexation was performed by measuring the absorbance at 562 nm every 3 min. The full spectra were scanned from 400 nm-800 nm with a step size of 2 nm.

SAXS sample preparation for iron core reduction in 3D lattice:

The 3D ferritin/Octa lattices (30 nM, 20 μL) were loaded in quartz capillaries and left to precipitate overnight at 4 °C. A sodium ascorbate stock solution (200 mM) was prepared freshly in deionized water prior to experiment. The working solution was prepared by mixing 2 μL of the ascorbate stock solution with 2 μL of the ammonium acetate stock solution (as described above) and added to the lattice solution in the capillary.

Supplementary References

1 DiFabio, J. *et al.* The life science x-ray scattering beamline at NSLS-II. *AIP Conference Proceedings* **1741**, 030049, doi:10.1063/1.4952872 (2016).

2 Yang, L. Using an in-vacuum CCD detector for simultaneous small- and wide-angle scattering at beamline X9. *Journal of Synchrotron Radiation* **20**, 211-218, doi:doi:10.1107/S0909049512048984 (2013).

3 Yager, K. G., Zhang, Y., Lu, F. & Gang, O. Periodic lattices of arbitrary nano-objects: modeling and applications for self-assembled systems. *Journal of Applied Crystallography* **47**, 118-129, doi:10.1107/S160057671302832X (2014).

4 Yager, K. G. ScatterSim. *https://github.com/CFN-softbio/ScatterSim* (2017).

5 Tian, Y. *et al.* Ordered three-dimensional nanomaterials using DNA-prescribed and valence-controlled material voxels. *Nature Materials* **19**, 789-796, doi:10.1038/s41563-019-0550-x (2020).

6 Scheres, S. H. W. RELION: Implementation of a Bayesian approach to cryo-EM structure determination. *Journal of Structural Biology* **180**, 519-530, doi:https://doi.org/10.1016/j.jsb.2012.09.006 (2012).

7 Zivanov, J. *et al.* New tools for automated high-resolution cryo-EM structure determination in RELION-3. *Elife* **7**, doi:10.7554/eLife.42166 (2018).

8 Rohou, A. & Grigorieff, N. CTFFIND4: Fast and accurate defocus estimation from electron micrographs. *Journal of Structural Biology* **192**, 216-221, doi:https://doi.org/10.1016/j.jsb.2015.08.008 (2015).

9 Scheres, S. H. W. & Chen, S. Prevention of overfitting in cryo-EM structure determination. *Nature Methods* **9**, 853-854, doi:10.1038/nmeth.2115 (2012).

10 Pettersen, E. F. *et al.* UCSF Chimera—A visualization system for exploratory research and analysis. *Journal of Computational Chemistry* **25**, 1605-1612, doi:https://doi.org/10.1002/jcc.20084 (2004).

11 Zheng, S. Q. *et al.* MotionCor2: anisotropic correction of beam-induced motion for improved cryo-electron microscopy. *Nature Methods* **14**, 331-332, doi:10.1038/nmeth.4193 (2017).

12 Kremer, J. R., Mastronarde, D. N. & McIntosh, J. R. Computer Visualization of Three-Dimensional Image Data Using IMOD. *Journal of Structural Biology* **116**, 71-76, doi:https://doi.org/10.1006/jsbi.1996.0013 (1996).

13 Zhang, K. Gctf: Real-time CTF determination and correction. *Journal of Structural Biology* **193**, 1-12, doi:https://doi.org/10.1016/j.jsb.2015.11.003 (2016).

14 Fernández, J. J., Li, S. & Crowther, R. A. CTF determination and correction in electron cryotomography. *Ultramicroscopy* **106**, 587-596, doi:https://doi.org/10.1016/j.ultramic.2006.02.004 (2006).

15 Zhang, L. & Ren, G. IPET and FETR: Experimental Approach for Studying Molecular Structure Dynamics by Cryo-Electron Tomography of a Single-Molecule Structure. *PLOS ONE* **7**, 1-19, doi:10.1371/journal.pone.0030249 (2012).

16 Zhai, X. *et al.* LoTToR: An Algorithm for Missing-Wedge Correction of the Low-Tilt Tomographic 3D Reconstruction of a Single-Molecule Structure. *Scientific Reports* **10**, 10489, doi:10.1038/s41598-020-66793-1 (2020).

17 Ludtke, S. J., Baldwin, P. R. & Chiu, W. EMAN: Semiautomated Software for High-Resolution Single-Particle Reconstructions. *Journal of Structural Biology* **128**, 82-97, doi:https://doi.org/10.1006/jsbi.1999.4174 (1999).
